# Supplementary material for: Thermo-responsive chiral micelles as recyclable organocatalyst for asymmetric Rauhut-Currier reaction in water
Source: Nat Commun. 2023 Nov 10;14:7287. doi: 10.1038/s41467-023-43092-7 (PMC10638429; doi:10.1038/s41467-023-43092-7)
Supplement: Supplementary file 1 — Supplementary Information [file 41467_2023_43092_MOESM1_ESM.pdf]

Supplementary Information

Thermo-Responsive Chiral Micelles as Recyclable Organocatalyst for Asymmetric Rauhut-Currier Reaction in Water

Lei Xu,[1,4,#] Li Zhou,[2,#] Yan-Xiang Li,[2,#] Run-Tan Gao,[1] Zheng Chen,[1] Na Liu,[3] and Zong-Quan Wu[1,*]

[1]State Key Laboratory of Supramolecular Structure and Materials, College of Chemistry, Jilin University, Changchun 130012, China.

[2]Department of Polymer Science and Engineering, Hefei University of Technology, Hefei 230009, China.

[3]The School of Pharmaceutical Sciences, Jilin University, 1266 Fujin Road, Changchun, Jilin 130021, China.

[4]Key Laboratory of Green and Precise Synthetic Chemistry and Applications, Ministry of Education, Huaibei Normal University, Huaibei, Anhui 235000, China.

Methods and materials ········· S3-S4

Polymers synthesis and characterization ········· S4-S5

Preparation of micelles ········· S5-S6

Critical aggregation concentration measurement ········· S6

Dynamic light scattering analysis ········· S6

AFM and TEM measurements ········· S6 -S7

General procedure for R-C reaction and recycling process ········· S7

Synthesis and characterization of **5** ········· S7-S16

Kinetic studies ········· S16

Supplementary Table 1. Characterization Data for the Polymers ········· S17

Supplementary Figs 1-9. ^{1}H, ^{13}P NMR and FT-IR spectra ········· S17-S21

Supplementary Figs 10-12. SEC, CD and UV-vis spectra ········· S22-S23

Supplementary Fig 13. The critical aggregation concentration data ········· S23

Supplementary Fig 14-16. TEM, DLS and AFM of polymer assemblies ········· S24

Supplementary Fig 17. Time-dependent HPLC for R-C reaction ········· S25

Supplementary Fig 18. Yield and *ee* values of **5aa** ········· S25

Supplementary Fig 19-20. ^{31}P NMR and Raman data ········· S26

Supplementary Figs 21-46. ^{1}H and ^{13}C NMR spectra of **5** ········· S27-S39

Supplementary Figs 47-65. ^{1}H NMR of **3** and **4** ········· S40-S49

Supplementary Figs 66-104. HPLC curves for **5** ········· S49-S68

Supplementary Figs 105-109. HRMS (ESI-FT) spectrum of **5** ········· S69-S71

Supplementary references ········· S72-S73

Methods and Materials

Methods

Nuclear magnetic resonance (NMR) spectra were recorded using a Bruker 600 MHz or 400 MHz spectrometer {H} operated in the Fourier Transform mode. Chemical shifts are reported in delta (δ) units and expressed in parts per million (ppm) downfield from tetramethylsilane (TMS) using the residual solvent proton as an internal standard. Size exclusion chromatography (SEC) was performed on Waters 1515 pump and Waters 2414 differential refractive index (RI) detector (set at 40 °C) using a series of two linear TSK gel GMH_{HR}-H columns. Molecular weight (M_n) and its dispersity (M_w/M_n) data are reported relative to polystyrene standards. The eluent was tetrahydrofuran (THF) at a flow rate of 0.8 mL/min. FT-IR spectra were recorded on Perkin-Elmer Spectrum BX FT-IR system using KBr pellets. Circular dichroism (CD) spectra were obtained in a 1.0 mm quartz cell at 25 °C using a JASCO J1500 spectropolarimeter. Absorption spectra were recorded on UNIC 4802 UV/vis double beam spectrophotometer in a 1.0 cm quartz cell at 25 °C. The optical rotations were measured in $CHCl_3$ at room temperature using a 10.0 cm quartz cell on a WZZ-2S polarimeter. Atomic force microscope (AFM) was performed on a Cypher S microscope (Oxford Instruments, Asylum Research). Transmission electron microscopy (TEM) was performed on a JEM1400FLASH operating at 120.0 kV accelerating voltage. Dynamic light scattering (DLS) was recorded using a Nano-ZS 90 Zetasizer of Malvern (UK) instrument. High performance liquid chromatography (HPLC) was carried out on SHIMADZU LC-20AT equipment with UV-vis detector or on a JASCO Pu-418 pump with JASCO UV-4070 and CD-4095 detectors, using *n*-hexane/i-PrOH as fluent on chiral column. UV-vis absorption spectra were carried on UNIC 4802 UV/vis double beam spectrophotometer

instrument using transmission model. HRMS analyses were performed on a Thermo Scientific Vanquish Q Exactive Plus system. Raman spectra were recorded on a Thermo Scientific Nicolet NXR FT-Raman Spectrometer with the 532 nm laser (100% power, 50 mW; exposure time, 5 s) as a light source.

Materials.

All solvents were obtained from Sinopharm. Co. Ltd., and were purified by the standard procedures before use. In experiments that required dry solvents, tetrahydrofuran (THF) and chloroform ($CHCl_3$) were dried using standard methods and distilled before use. All chemicals were purchased from Aladdin, Sinopharm, and Sigma-Aldrich Chemical Co. Ltd., and were used as received otherwise denoted. The alkyne-Pd(II) catalyst, monomer **1r**, **1s**, and **2** were prepared following the reported procedures, and the structures were confirmed by ^{1}H NMR[1-3]. The compounds **3** and **4** for Rauhut-Currier (R-C) reaction were synthesized following the reported literatures with modifications[4,5]. The deuteration-labeled **3a-**d^1 and **4e-**d^2 was synthesized according to the literature.[4-6]

Polymers synthesis and characterization.

The synthetic procedure for the polymers were described in main text.

Characterization data for poly-**1r**$_{50}$. SEC: M_n = 23.1 kDa, M_w/M_n = 1.22; ^{1}H NMR (600 MHz, $CDCl_3$, 25 °C): δ 8.40–5.80 (br, NH and ArH, 24H), 5.65–5.31 (br, CH, 1H), 2.68–2.09 (br, CH_2, 2H); FT-IR (KBr, cm^{-1}): ν 3057 ($\nu_{C–H}$), 2921 ($\nu_{C–H}$), 1648 ($\nu_{NHC=O}$), 1600 ($\nu_{C=N}$); $[\alpha]^{25}_D$ = –893 (0.1, $CHCl_3$).

The poly-**1r**$_{50}$ was prepared following the same procedure to poly-**1r**$_{50}$ by using **1r** in 90% yield. SEC: M_n = 22.8 kDa, M_w/M_n = 1.19; ^{1}H NMR (600 MHz, $CDCl_3$, 25 °C): δ 8.16–5.68 (br, NH and ArH, 24H), 5.60–5.24 (br, CH, 1H), 2.70–1.92 (br, CH_2, 2H); FT-IR (KBr, cm^{-1}): ν 3059 ($\nu_{C–H}$), 2925 ($\nu_{C–H}$), 1644 ($\nu_{NHC=O}$), 1601 ($\nu_{C=N}$); $[\alpha]^{25}_D$ = +835 (0.1, $CHCl_3$).

Characterization data for poly(**1s**$_{50}$-*b*-**2**$_{100}$). SEC: M_n = 84.2 kDa, M_w/M_n = 1.25; ^{1}H NMR (600 MHz, $CDCl_3$, 25 °C): δ 8.52–5.96 (br, NH and ArH, 32H), 5.92–5.47 (br, CH, 1H), 5.40–5.32 (br, CH_2, 4H), 4.32–2.84 (br, CH_2 and CH_3, 90H), 2.72–1.84 (br, CH_2, 2H); FT-IR (KBr, cm^{-1}): ν 3056 ($\nu_{C–H}$), 2876 ($\nu_{C–H}$), 1716 ($\nu_{NHC=O}$), 1646 ($\nu_{OC=O}$), 1600 ($\nu_{C=N}$), 1095 ($\nu_{C–O–C}$); $[\alpha]^{25}_D$ = –632 (0.1, $CHCl_3$).

The poly(**1r**$_{50}$-*b*-**2**$_{100}$) was prepared following the same procedure to that of poly(**1s**$_{50}$-*b*-**2**$_{100}$). SEC: M_n = 85.6 kDa, M_w/M_n = 1.29; ^{1}H NMR (600 MHz, $CDCl_3$, 25 °C): δ 8.43–5.94 (br, NH and ArH, 32H), 5.89–5.44 (br, CH, 1H), 5.41–5.28 (br, CH_2, 4H), 4.30–2.86 (br, CH_2 and CH_3, 90H), 2.68–1.86 (br, CH_2, 2H); FT-IR (KBr, cm^{-1}): ν 3057 ($\nu_{C–H}$), 2872 ($\nu_{C–H}$), 1713 ($\nu_{NHC=O}$), 1642 ($\nu_{OC=O}$), 1600 ($\nu_{C=N}$), 1094 ($\nu_{C–O–C}$); $[\alpha]^{25}_D$ = +629 (0.1, $CHCl_3$).

Preparation of micelle. Taking poly(**1s**$_{50}$-*b*-**2**$_{100}$) as an example. Typically, a THF (6.0 mL) solution of poly(**1s**$_{50}$-*b*-**2**$_{100}$) (100 mg) was prepared in advance. Under vigorous stirring, deionized water (100 mL) was added via a syringe pump at a flow rate of 0.05 mL/min. After the addition, the dispersion was left stirring for another 12 h. THF was then removed by dialysis (MWCO 3.5 kDa) against pure water for 24 h. Fresh water was replaced approximately every 5 h. The obtained dispersion did not

exhibit any macroscopic phase separation upon standing at room temperature for more than one week, suggesting the formation of a stable micelle.

Critical aggregation concentration (CAC) measurements. Taking poly(**1s**$_{50}$-*b*-**2**$_{100}$) as an example. The CAC of poly(**1s**$_{50}$-*b*-**2**$_{100}$) in aqueous solutions were measured using pyrene as a fluorescent probe, and the CAC measurement was performed as follows. Pyrene (2.0 mg) was dissolved in 10 mL of acetone and then 10 μL of the solution was added into each cuvette. The acetone was allowed to evaporate. Then 2.0 mL of poly(**1s**$_{50}$-*b*-**2**$_{100}$) aqueous solutions with concentrations ranging from 1.95 to 1000 mg/L were added into the pyrene-containing cuvette separately. Upon sonication for 10 min, the solutions were kept at room temperature and equilibrated for 24 h before fluorescent emission measurements with an excitation wavelength of 335 nm. The emission spectra were recorded in the 340-600 nm wavelength range. For each spectrum obtained, the intensity ratio of the first and third peaks, I_1/I_3, was calculated. The CAC was estimated as the concentration at which I_1/I_3 began to drop, indicating that polymer aggregation occurred.

Dynamic light scattering (DLS) analysis. DLS measurements were carried on a Nano-ZS90 Zetasizer of Malvern (UK) instrument, and all data were averaged over three-time measurements.

AFM measurements. Stock solutions of poly(**1s**$_{50}$-*b*-**2**$_{100}$) in aqueous solutions (0.02 mg/mL) were prepared. Samples for AFM measurements were prepared by casting 20 μL aliquots of the stock solutions of the polymers on freshly cleaved silicon substrate. The samples on silicon were dried in air and then subjected to AFM measurements.

TEM measurement. TEM observations were collected on a JEM1400FLASH electron microscope at an acceleration voltage of 120.0 kV, and the samples were prepared by casting the aqueous solutions of poly(**1s**$_{50}$-*b*-**2**$_{100}$) onto copper mesh grids and drying in air under ambient temperature.

General procedure for R-C reaction and recycling process of the polymer catalyst. A stirred solution of **3** (0.1 mmol) and polymer catalyst micelle (4 mol%, determined based on the elemental analysis) in the solution of H_2O/THF (v/v = 100/5) was cooled to 0 °C. Subsequently, **4** (0.3 mmol) was added in one portion through a syringe. The mixture was stirred at this temperature until completion of **3** as indicated by TLC, after completion of the reaction, the aqueous solution was heated to 55 °C, higher than the cloud point of polymer catalyst in water. The transparent solution immediately turned turbid due to the precipitation of polymer catalyst. The precipitated solid was separated by high-speed centrifugation and filtrated by medium flow qualitative filter paper with bore diameter of 30-50 μm, then washed completely using *n*-hexane to remove the residues of the product and unreacted substrates. The filtrate containing the R-C reaction product was purified and subjected to further analyses. The precipitated solid of the polymer catalyst was reused in the R-C reaction.

The characterization data for compounds **5** were showed below.

Characterization data for 5:

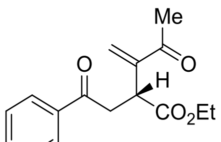

R-**5aa**

(*R*)-Ethyl 3-methylene-4-oxo-2-(2-oxo-2-phenylethyl) pentanoate (*R*-5aa): Reaction time: 2 days. White solid; 81% yield; 96% *ee*. ^{1}H NMR (600 MHz, $CDCl_3$, 25 °C): δ 7.95 (d, J = 7.2 Hz, 2H), 7.59–7.51 (m, 1H), 7.45 (m, 2H), 6.19 (s, 1H), 6.05 (s, 1H), 4.23–4.21 (m, 1H), 4.16–4.12 (q, J = 6.8, 2H), 3.71 (dd, J = 18.0, 8.0 Hz, 1H), 3.17 (dd, J = 18.0, 5.8 Hz, 1H), 2.37 (s, 3H), 1.20 (t, J = 7.2 Hz, 3H).

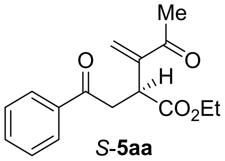

S-**5aa**

(*S*)-Ethyl 3-methylene-4-oxo-2-(2-oxo-2-phenylethyl) pentanoate (*S*-5aa): Reaction time: 2 days. White solid; 82% yield; 95% *ee*. ^{1}H NMR (600 MHz, $CDCl_3$, 25 °C): δ 7.94 (d, J = 7.2 Hz, 2H), 7.58–7.51 (m, 1H), 7.48–7.40 (m, 2H), 6.19 (s, 1H), 6.05 (s, 1H), 4.26–4.18 (m, 1H), 4.18–4.08 (q, J = 7.2, 2H), 3.71 (dd, J = 17.8, 7.8 Hz, 1H), 3.18 (dd, J = 17.8, 5.8 Hz, 1H), 2.36 (s, 3H), 1.20 (t, J = 7.2 Hz, 3H).

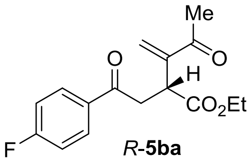

R-**5ba**

(*R*)-Ethyl 2-(2-(4-fluorophenyl)-2-oxoethyl)-3-methylene-4-oxopentanoate (*R*-5ba): Reaction time: 2 days; White solid; 87% yield; 93% *ee*. ^{1}H NMR (600 MHz, $CDCl_3$, 25 °C): δ 8.01–7.94 (m, 2H), 7.16–7.07 (m, 2H), 6.19 (s, 1H), 6.05 (s, 1H), 4.26–4.18 (m, 1H), 4.17–4.08 (m, 2H), 3.69 (dd, J = 17.8, 8.0 Hz, 1H), 3.11 (dd, J = 17.8, 5.6 Hz, 1H), 2.37 (s, 3H), 1.20 (t, J = 7.2 Hz, 3H).

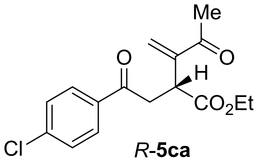

R-**5ca**

(*R*)-Ethyl 2-(2-(4-chlorophenyl)-2-oxoethyl)-3-methylene-4-oxopentanoate (*R*-5ca): Reaction time: 2 days; White solid; 90% yield; 94% *ee*. ^{1}H NMR (600 MHz, $CDCl_3$, 25 °C): δ 7.89 (d, *J* = 8.6 Hz, 2H), 7.42 (d, *J* = 8.6 Hz, 2H), 6.20 (s, 1H), 6.05 (s, 1H), 4.20 (dd, *J* = 4.0, 5.6 Hz, 1H), 4.13 (q, *J* = 7.2 Hz, 2H), 3.68 (dd, *J* = 18.0, 8.0 Hz, 1H), 3.09 (dd, *J* = 18.0, 5.6 Hz, 2H), 2.37 (s, 1H), 1.20 (t, *J* = 8.2 Hz, 3H).

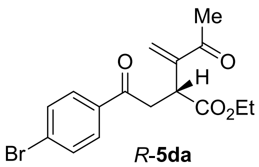

R-**5da**

(*R*)-Ethyl 2-(2-(4-bromophenyl)-2-oxoethyl)-3-methylene-4-oxopentanoate (*R*-5da): Reaction time: 2 days; white solid; 86% yield; 95% *ee*. ^{1}H NMR (600 MHz, $CDCl_3$, 25 °C): δ 7.81 (d, *J* = 7.8 Hz, 2H), 7.58 (d, *J* = 8.0 Hz, 2H), 6.19 (s, 1H), 6.05 (s, 1H), 4.20 (t, *J* = 6.0 Hz, 1H), 4.16–4.08 (m, 2H), 3.68 (dd, *J* = 17.8, 8.0 Hz, 1H), 3.09 (dd, *J* = 18.0, 6.2 Hz, 1H), 2.37 (s, 1H), 1.30–1.10 (m, 3H).

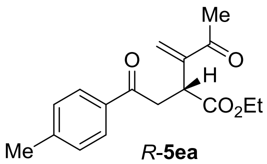

R-**5ea**

(*R*)-Ethyl 3-methylene-4-oxo-2-(2-oxo-2-(p-tolyl)ethyl)pentanoate (*R*-5ea): Reaction time: 2 days; white solid; 80% yield; 96% *ee*. ^{1}H NMR (600 MHz, $CDCl_3$, 25 °C): δ 7.85 (d, *J* = 8.2 Hz, 2H), 7.24 (d, *J* = 7.8 Hz, 2H), 6.19 (s, 1H), 6.05 (s, 1H), 4.21 (dd, *J* = 7.6, 5.8 Hz, 1H), 4.14 (q, *J* = 7.2 Hz, 2H), 3.68 (dd, *J* = 17.8, 7.6 Hz, 1H), 3.16 (dd, *J* = 17.8, 5.8 Hz, 1H), 2.40 (s, 3H), 2.37 (s, 3H), 1.20 (t, *J* = 7.2 Hz, 3H).

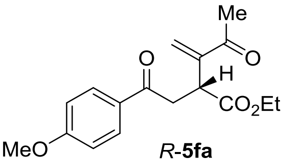

R-**5fa**

(*R*)-Ethyl 2-(2-(4-methoxyphenyl)-2-oxoethyl)-3-methylene-4-oxopentanoate (*R*-5fa): Reaction time: 2 days; white solid; 84% yield; 95% *ee*. ^{1}H NMR (600 MHz, $CDCl_3$, 25 °C): δ 7.93 (d, *J* = 9.0 Hz, 2H), 6.91 (d, *J* = 9.0 Hz, 2H), 6.18 (s, 1H), 6.05 (s, 1H), 4.20 (dd, *J* = 7.8, 5.8 Hz, 1H), 4.14 (q, *J* = 7.2 Hz, 2H), 3.86 (s, 3H), 3.65 (dd, *J* = 17.6, 7.6 Hz, 1H), 3.14 (dd, *J* = 17.8, 5.8 Hz, 1H), 2.37 (s, 3H), 1.20 (t, *J* = 7.2 Hz, 3H).

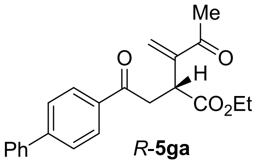

R-**5ga**

(*R*)-Ethyl 2-(2-([1,1'-biphenyl]-4-yl)-2-oxoethyl)-3-methylene-4-oxopentanoate (*R*-5ga): Reaction time: 2 days; white solid; 81% yield; 96% *ee*. ^{1}H NMR (600 MHz, $CDCl_3$, 25 °C): δ 8.03 (d, *J* = 8.6 Hz, 2H), 7.68 (d, *J* = 8.6 Hz, 2H), 7.64–7.60 (m, 2H), 7.50–7.44 (m, 2H), 7.42–7.35 (m, 1H), 6.21 (s, 1H), 6.08 (s, 1H), 4.25 (dd, *J* = 7.8, 5.6 Hz, 1H), 4.16 (q, *J* = 7.2 Hz, 2H), 3.75 (dd, *J* = 17.8, 7.8 Hz, 1H), 3.20 (dd, *J* = 18.0, 5.8 Hz, 1H), 2.39 (s, 3H), 1.22 (t, *J* = 7.2 Hz, 3H).

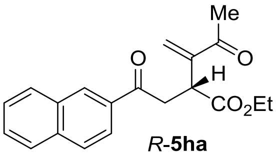

R-**5ha**

(*R*)-Ethyl 3-methylene-2-(2-(naphthalen-2-yl)-2-oxoethyl)-4-oxopentanoate (*R*-5ha): Reaction time: 2 days; white solid; 83% yield; 96% *ee*. ^{1}H NMR (600 MHz, $CDCl_3$, 25 °C): δ 8.48 (s, 1H), 8.04–7.98 (m, 1H), 7.95 (d, *J* = 8.2 Hz, 1H), 7.87 (t, *J* =

9.0 Hz, 2H), 7.63–7.57 (m, 1H), 7.56–7.51 (m, 1H), 6.21 (s, 1H), 6.10 (s, 1H), 4.33–4.24 (m, 1H), 4.16 (q, *J* = 7.2 Hz, 2H), 3.86 (dd, *J* = 17.8, 7.6 Hz, 1H), 3.32 (dd, *J* = 17.8, 5.8 Hz, 1H), 2.39 (s, 3H), 1.22 (t, *J* = 7.2 Hz, 1H).

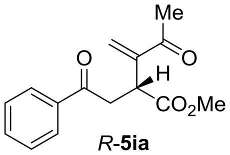

R-**5ia**

(*R*)-Methyl 3-methylene-4-oxo-2-(2-oxo-2-phenylethyl) pentanoate (*R*-5ia): Reaction time: 2 days; white solid; 85% yield; 95% *ee*. ^{1}H NMR (600 MHz, $CDCl_3$, 25 °C): δ 7.95 (m, 2H), 7.56 (m, 1H), 7.45 (t, *J* = 7.6 Hz, 2H), 6.20 (s, 1H), 6.07 (s, 1H), 4.25 (dd, *J* = 7.6, 5.6 Hz, 1H), 3.72 (dd, *J* = 17.8, 7.8 Hz, 1H), 3.68 (s, 3H), 3.18 (dd, *J* = 17.6, 5.4 Hz, 1H), 2.37 (s, 3H).

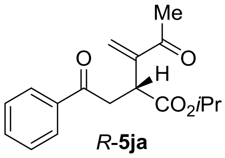

R-**5ja**

(*R*)-Isopropyl 3-methylene-4-oxo-2-(2-oxo-2-phenylethyl)pentanoate (*R*-5ja): Reaction time: 2 days; white solid; 83% yield; ^{1}H NMR (600 MHz, $CDCl_3$, 25 °C): 95% *ee*. ^{1}H NMR (600 MHz, $CDCl_3$, 25 °C): δ 7.95 (d, *J* = 7.4 Hz, 2H), 7.55 (t, *J* = 7.4 Hz, 1H), 7.44 (t, *J* = 7.8 Hz, 2H), 6.17 (s, 1H), 6.04 (s, 1H), 5.05–4.93 (m, 1H), 4.19 (dd, *J* = 7.6, 6.0 Hz, 1H), 3.70 (dd, *J* = 18.0, 7.8 Hz, 1H), 3.15 (dd, *J* = 17.8, 5.6 Hz, 1H), 2.36 (s, 3H), 1.22 (d, *J* = 6.2 Hz, 3H), 1.14 (d, *J* = 6.4 Hz, 3H).

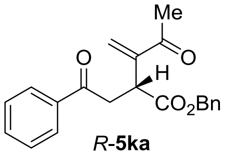

R-**5ka**

(*R*)-Benzyl 3-methylene-4-oxo-2-(2-oxo-2-phenylethyl) pentanoate (*R*-5ka): Reaction time: 2 days; white solid; 82% yield; 96% *ee*. ^{1}H NMR (600 MHz, $CDCl_3$,

25 °C): δ 7.94 (d, *J* = 8.4 Hz, 2H), 7.60–7.52 (m, 1H), 7.44 (t, *J* = 7.4 Hz, 2H), 7.37–7.26 (m, 5H), 6.16 (s, 1H), 6.01 (s, 1H), 5.13 (q, *J* = 12.4 Hz, 2H), 4.33–4.27 (m, 1H), 3.74 (dd, *J* = 17.4, 7.0 Hz, 1H), 3.21 (dd, *J* = 18.4, 6.4 Hz, 1H), 2.34 (s, 3H).

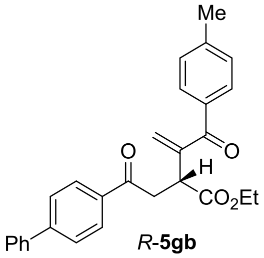

R-**5gb**

(*R*)-Ethyl-2-(2-([1,1'-biphenyl]-4-yl)-2-oxoethyl)-3-(4-methylbenzoyl)but-3-enoate (*R*-5gb): Reaction time: 2 days; white solid; M.p. = 94–95 °C; $[\alpha]_D^{20}$ = + 103.7 (*c* = 0.33, $CHCl_3$); 70% yield; 92% *ee*. ^{1}H NMR (600 MHz, $CDCl_3$, 25 °C): δ 8.10–8.05 (m, 2H), 7.72–7.65 (m, 4H), 7.65–7.60 (m, 2H), 7.50–7.45 (m, 2H), 7.42–7.38 (m, 1H), 7.26–7.21 (m, 2H), 6.06 (s, 1H), 5.78 (s, 1H), 4.40 (dd, *J* = 7.8, 5.4 Hz, 1H), 4.23–4.10 (m, 2H), 3.88 (dd, *J* = 17.8, 7.8 Hz, 1H), 3.44 (dd, *J* = 17.8, 5.8 Hz, 1H), 2.42 (s, 3H), 1.18 (t, *J* = 7.2 Hz, 3H); ^{13}C NMR (150 MHz, $CDCl_3$, 25 °C): δ 197.3, 196.8, 172.6, 150.0, 145.2, 143.4, 139.9, 135.3, 134.7, 129.9, 129.0, 128.8, 128.3, 127.8, 127.4, 127.3, 61.3, 43.8, 40.4, 21.7, 14.1; FT-IR (KBr, cm^{-1}): ν 3027, 3001, 1733, 1673, 1649, 1605, 1174; MS (ESI): *m/z* calcd for $C_{28}H_{27}O_4$ ([M + H]$^+$): 427.18; Found: 427.18; Anal. Calcd (%) for $C_{28}H_{26}O_4$: C, 78.85; H, 6.14. Found (%): C, 78.87; H, 6.13. HRMS (ESI-FT): Calcd. For [M + H]$^+$, $C_{28}H_{27}O_4$: 427.1907. Found: 427.1908.

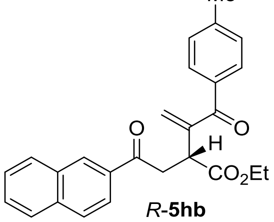

R-**5hb**

(*R*)-Ethyl-3-(4-methylbenzoyl)-2-(2-(naphthalen-2-yl)-2-oxoethyl)but-3-enoate (*R*-5hb): Reaction time: 2 days; white solid; 72% yield; M.p. = 97–98 °C; $[\alpha]_D^{20}$ = + 101.8 (*c* = 0.33, $CHCl_3$); 90% *ee*. ^{1}H NMR (600 MHz, $CDCl_3$, 25 °C): *δ* 8.54 (s, 1H), 8.08–8.03 (m, 1H), 7.97 (d, *J* = 8.0 Hz, 1H), 7.88 (dd, *J* = 13.0, 8.4 Hz, 2H), 7.71 (d, *J* = 8.0 Hz, 2H), 7.60 (t, *J* = 7.4 Hz, 1H), 7.55 (t, *J* = 7.4 Hz, 1H), 7.28–7.23 (m, 2H) ,6.09 (s, 1H), 5.80 (s, 1H), 4.44 (dd, *J* = 7.8, 5.6 Hz, 1H), 4.23–4.12 (m, 2H), 4.00 (dd, *J* = 17.8, 7.8 Hz, 1H), 3.56 (dd, *J* = 17.8, 5.8 Hz, 1H), 2.42 (s, 3H), 1.19 (t, *J* = 7.2 Hz, 3H); ^{13}C NMR (150 MHz, $CDCl_3$, 25 °C): *δ* 197.6, 196.9, 172.6, 145.3, 143.4, 135.7, 134.7, 134.0, 132.6, 130.0, 129.7, 129.0, 128.6, 128.5, 127.9, 127.8, 126.9, 123.9, 61.3, 43.9, 40.4, 21.7, 14.1; FT-IR (KBr, cm^{-1}): *ν* 3058, 2980, 1729, 1679, 1651, 1627, 1605, 1171; MS (ESI): *m/z* calcd for $C_{26}H_{25}O_4$ ($[M + H]^+$): 401.17; Found: 401.20; Anal. Calcd (%) for $C_{26}H_{24}O_4$: C, 77.98; H, 6.04. Found (%): C, 77.96; H, 6.02. HRMS (ESI-FT): Calcd. For $[M + H]^+$, $C_{26}H_{25}O_4$: 401.1747. Found: 401.1747.

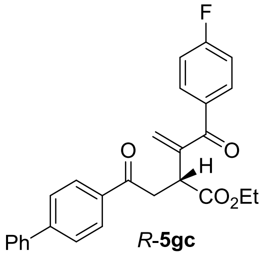

R-**5gc**

(*R*)-Ethyl-2-(2-([1,1'-biphenyl]-4-yl)-2-oxoethyl)-3-(4-fluorobenzoyl)but-3-enoate (*R*-5gc): Reaction time: 2 days; white solid; M.p. = 73–74 °C; $[\alpha]_D^{20}$ = + 92.6 (*c* = 0.33, $CHCl_3$); 69% yield; 91% *ee*. ^{1}H NMR (600 MHz, $CDCl_3$, 25 °C): *δ* 8.10–8.05 (m, 2H), 7.86–7.78 (m, 2H), 7.72–7.66 (m, 2H), 7.65–7.60 (m, 2H), 7.51–7.45 (m, 2H), 7.44–7.38 (m, 1H), 7.16–7.09 (m, 1H), 6.08 (s, 1H), 5.76 (s, 1H), 4.40 (t, *J* = 6.6

Hz, 1H), 4.22–4.10 (m, 2H), 3.87 (dd, *J* = 17.8, 7.2 Hz, 1H), 3.48 (dd, *J* = 17.8, 6.2 Hz, 1H) 1.18 (t, *J* = 7.2 Hz, 3H); ^{13}C NMR (150 MHz, $CDCl_3$, 25 °C): *δ* 197.2, 195.7, 172.5, 164.7, 146.1, 145.2, 139.9, 135.3, 132.4, 132.3, 129.1, 128.8, 128.4, 128.0, 127.4, 115.5, 61.4, 43.8, 40.3, 14.1; FT-IR (KBr, cm^{-1}): *ν* 3060, 2983, 1733, 1677, 1657, 1597, 1505, 1229; MS (ESI): *m/z* calcd for $C_{27}H_{24}FO_4$ ([M + H]$^+$): 431.16; Found: 431.20; Anal. Calcd (%) for $C_{27}H_{23}FO_4$: C, 77.33; H, 5.39. Found (%): C, 77.32; H, 5.40. HRMS (ESI-FT): Calcd. For [M + H]$^+$, $C_{27}H_{24}FO_4$: 431.1653. Found: 431.1655.

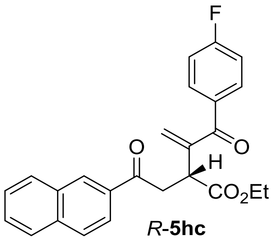

R-**5hc**

(*R*)-Ethyl-3-(4-fluorobenzoyl)-2-(2-(naphthalen-2-yl)-2-oxoethyl)but-3-enoate

(*R*-5hc): Reaction time: 2 days; white solid; M.p. = 75–76 °C; $[\alpha]_D^{20}$ = + 91.8 (*c* = 0.33, $CHCl_3$); 70% yield; 90% *ee*. ^{1}H NMR (600 MHz, $CDCl_3$, 25 °C): *δ* 8.54 (s, 1H), 8.05 (d, *J* = 8.4, 1H), 7.86–7.78 (m, 1H), 7.69 (d, *J* = 4.3 Hz, 2H), 7.64–7.58 (m, 1H), 7.58–7.53 (m, 1H), 7.17–7.09 (m, 2H), 6.10 (s, 1H), 5.78 (s, 1H), 4.44 (t, *J* = 6.8 Hz, 1H), 4.23–4.11 (m, 2H), 3.87 (dd, *J* = 17.8, 7.2 Hz, 1H), 3.48 (dd, *J* = 17.8, 6.4 Hz, 1H), 1.18 (t, *J* = 7.2 Hz, 3H); ^{13}C NMR (150 MHz, $CDCl_3$, 25 °C): *δ* 197.5, 195.7, 172.5, 165.5, 145.2, 135.8, 133.9, 133.6, 132.6, 132.4, 130.1, 129.7, 128.6, 128.1, 127.9, 126.9, 123.8, 115.6, 115.5, 61.4, 43.9, 40.3, 14.1; FT-IR (KBr, cm^{-1}): *ν* 3060, 2981, 1732, 1678, 1656, 1627, 1598, 1227; MS (ESI): *m/z* calcd for $C_{25}H_{22}FO_4$ ([M + H]$^+$): 405.14; Found: 405.18; Anal. Calcd (%) for $C_{25}H_{21}FO_4$: C, 74.25; H, 5.23. Found (%): C, 74.26; H, 5.22. HRMS (ESI-FT): Calcd. For [M + H]$^+$, $C_{25}H_{22}FO_4$: 405.1497. Found: 405.1497.

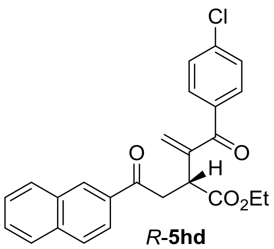

R-**5hd**

(*R*)-Ethyl-3-(4-chlorobenzoyl)-2-(2-(naphthalen-2-yl)-2-oxoethyl)but-3-enoate (*R*-5hd): Reaction time: 2 days; white solid; M.p. = 67–68 °C; $[\alpha]_D^{20}$ = + 86.7 (*c* = 0.33, $CHCl_3$); 71% yield; 93% *ee*. ^{1}H NMR (600 MHz, $CDCl_3$, 25 °C): *δ* 8.53 (s, 1H), 8.05 (dd, *J* = 8.6, 1.8 Hz, 1H), 7.97 (d, *J* = 8.2 Hz, 1H), 7.89 (dd, *J* = 14.0, 8.4 Hz, 2H), 7.77–7.70 (m, 2H), 7.64–7.58 (m, 1H), 7.58–7.53 (m, 1H), 7.47–7.37 (m, 2H), 6.12 (s, 1H), 5.78 (s, 1H), 4.44 (t, *J* = 6.6 Hz, 1H), 4.20–4.15 (m, 2H), 3.99 (dd, *J* = 17.8, 7.0 Hz, 1H), 3.59 (dd, *J* = 17.8, 6.4 Hz, 1H), 1.18 (t, *J* = 7.2 Hz, 3H); ^{13}C NMR (150 MHz, $CDCl_3$, 25 °C): *δ* 197.5, 196.0, 172.5, 145.1, 139.0, 135.8, 135.7, 133.9, 132.6, 131.2, 130.1, 129.7, 128.7, 128.6, 128.5, 127.9, 127.0, 123.8, 61.5, 43.8, 40.3, 14.1; FT-IR (KBr, cm^{-1}): *ν* 3057, 2985, 1730, 1677, 1656, 1626, 1587, 1171; MS (ESI): *m/z* calcd for $C_{25}H_{22}ClO_4$ ($[M + H]^+$): 421.11; Found: 421.16; Anal. Calcd (%) for $C_{25}H_{21}ClO_4$: C, 71.34; H, 5.03. Found (%): C, 71.32; H, 5.04. HRMS (ESI-FT): Calcd. For $[M + H]^+$, $C_{25}H_{22}FO_4$: 405.1497. Found: 405.1497.

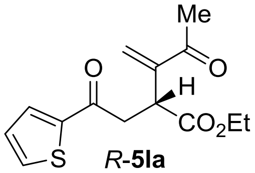

R-**5la**

(*R*)-ethyl 3-methylene-4-oxo-2-(2-oxo-2-(thiophen-2-yl)ethyl)pentanoate (*R*-5la) Reaction time: 2 days; white solid; 74% yield; 88% *ee*. ^{1}H NMR (600 MHz, $CDCl_3$) *δ* 7.73 (d, *J* = 3.8 Hz, 1H), 7.63 (d, *J* = 4.9 Hz, 1H), 7.12 (dd, *J* = 4.8, 4.0 Hz, 1H), 6.19

(s, 1H), 6.07 (s, 1H), 4.11–4.18 (m, 3H), 3.65 (dd, J = 17.2, 7.4 Hz, 1H), 3.14 (dd, J = 17.4, 6.2 Hz, 1H), 2.37 (s, 3H), 1.20 (t, J = 7.2 Hz, 3H).

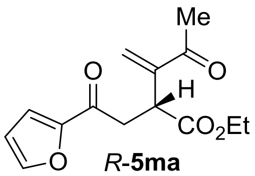

R-**5ma**

(*R*)-ethyl 2-(2-(furan-2-yl)-2-oxoethyl)-3-methylene-4-oxopentanoate (*R*-5ma) Reaction time: 2 days; white solid; 76% yield; 84% *ee*. ^{1}H NMR (600 MHz, $CDCl_3$) δ 7.57 (s, 1H), 7.19 (d, J = 3.6 Hz, 1H), 6.52 (dd, J = 3.6, 1.7 Hz, 1H), 6.18 (s, 1H), 6.04 (s, 1H), 4.23–4.03 (m, 3H), 3.57 (dd, J = 17.6, 7.6 Hz, 1H), 3.03 (dd, J = 17.6, 6.2 Hz, 1H), 2.36 (s, 3H), 1.19 (t, J = 7.2 Hz, 3H).

Kinetic studies. Compound **3a** (20.0 mg, 0.10 mmol), internal standard 1,4-dimethoxybenzene (13.8 mg, 0.10 mmol), and **4a** (21.0 mg, 0.30 mmol) were added to a solution of polymer catalyst in different volume ratios of H_2O and THF with 4 mol% catalyst loading. The reaction solution was stirred at 25 °C for 48 hours. A series of aliquots were taking out from the reaction solution at appropriate time intervals for further analyses. The conversion of monomer **3a** was calculated based on the peak area of unreacted **3a** relative to that of the internal standard 1,4-dimethoxybenzene on the basis of the linear calibration curve.

Supplementary Table 1. Characterization Data for the Synthetic Polymers.

run	Polymers	$M_n{}^a$ (kDa)	$M_w/M_n{}^a$	Yield[b]	$\Delta\varepsilon_{374}{}^b$	$[\alpha]^{25}_D{}^d$
1	poly-**1s**$_{50}$	23.1	1.22	88%	–9.82	–893
2	poly(**1s**$_{50}$-*b*-**2**$_{50}$)	53.3	1.26	87%	–8.05	–797
3	poly(**1s**$_{50}$-*b*-**2**$_{100}$)	84.2	1.25	88%	–6.11	–632
4	poly(**1s**$_{50}$-*b*-**2**$_{150}$)	109.7	1.35	89%	–5.13	–426
5	poly-**1r**$_{50}$	22.8	1.19	90%	+9.34	+835
6	poly(**1r**$_{50}$-*b*-**2**$_{50}$)	53.7	1.27	85%	+7.97	+741
7	poly(**1r**$_{50}$-*b*-**2**$_{100}$)	85.6	1.29	86%	+5.86	+629
8	poly(**1r**$_{50}$-*b*-**2**$_{150}$)	112.3	1.38	84%	+4.82	+403

[a]The M_n and M_w/M_n were determined by SEC with equivalent to polystyrene standards. [b]The isolated yields of the polymerizations. [c]The CD intensity (M^{-1} cm^{-1}) at 374 nm of the polymers measured in THF at 25 °C. [d]The optical rotation was measured in $CHCl_3$ at room temperature (*c* = 0.1).

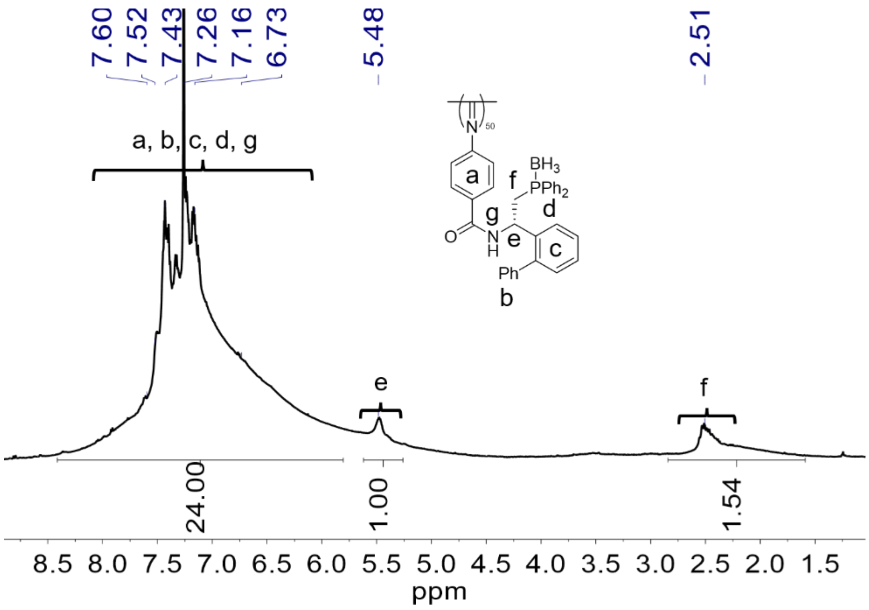

Supplementary Fig. 1. ^{1}H NMR (600 MHz) spectrum of poly-**1s**$_{50}$ measured in $CDCl_3$ at 25 °C.

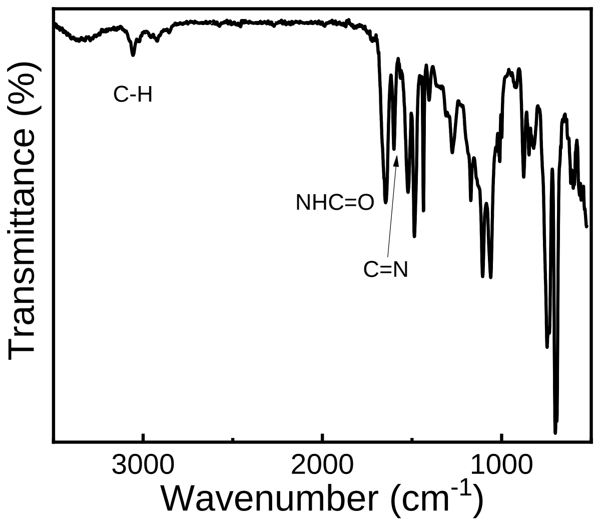

Supplementary Fig. 2. FT-IR spectrum of poly-**1s**$_{50}$ measured at 25 °C using KBr pellets.

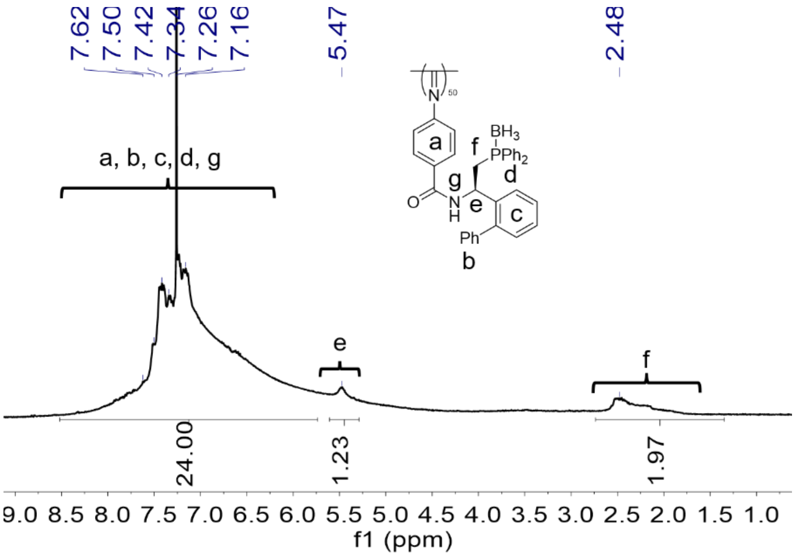

Supplementary Fig. 3. ^{1}H NMR (600 MHz) spectrum of poly-**1r**$_{50}$ measured in $CDCl_3$ at 25 °C.

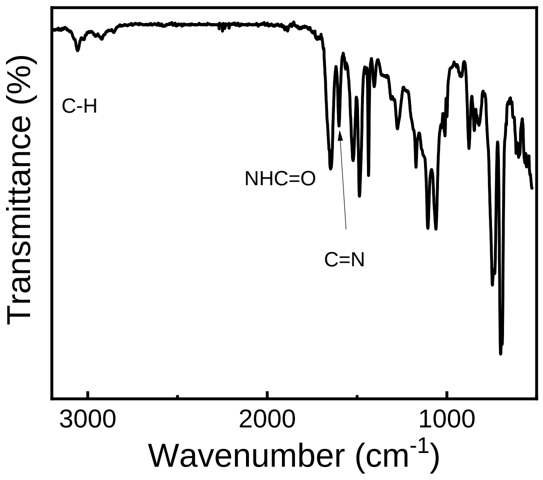

Supplementary Fig. 4. FT-IR spectrum of poly-**1r**$_{50}$ measured at 25 °C using KBr pellets.

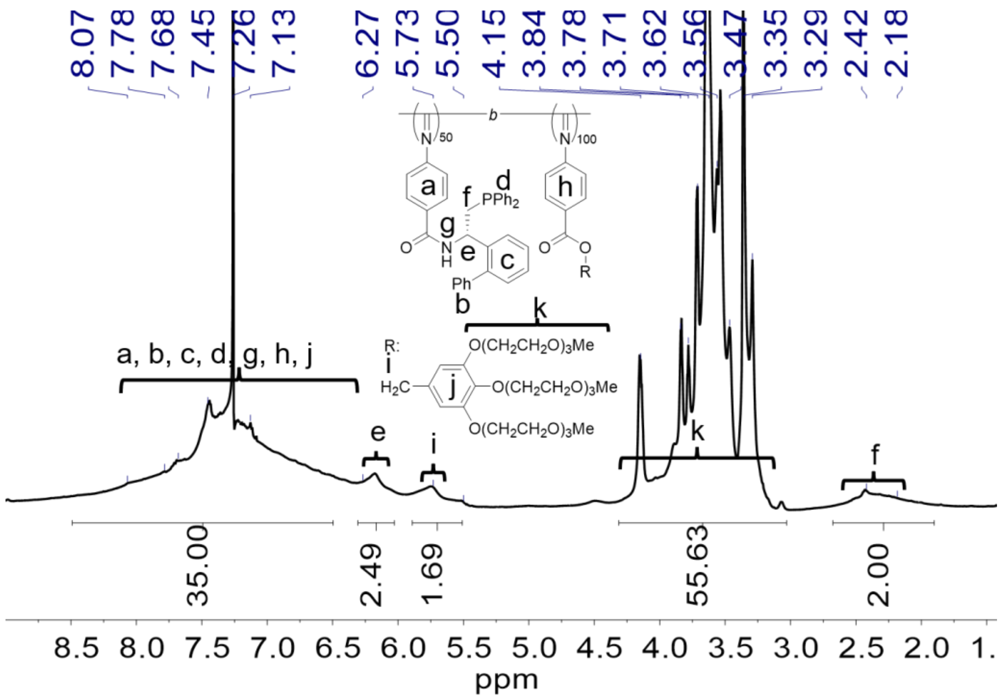

Supplementary Fig. 5. ^{1}H NMR (600 MHz) spectrum of poly(**1s**$_{50}$-*b*-**2**$_{100}$) measured in $CDCl_3$ at 25 °C.

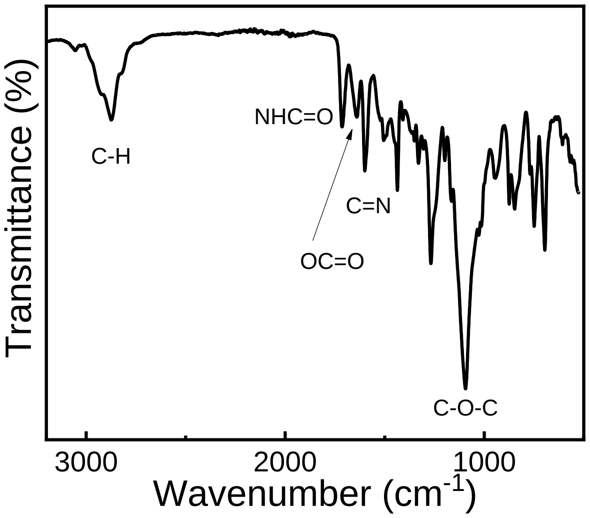

Supplementary Fig. 6. FT-IR spectrum of poly(**1s**$_{50}$-*b*-**2**$_{100}$) measured at 25 °C using KBr pellets.

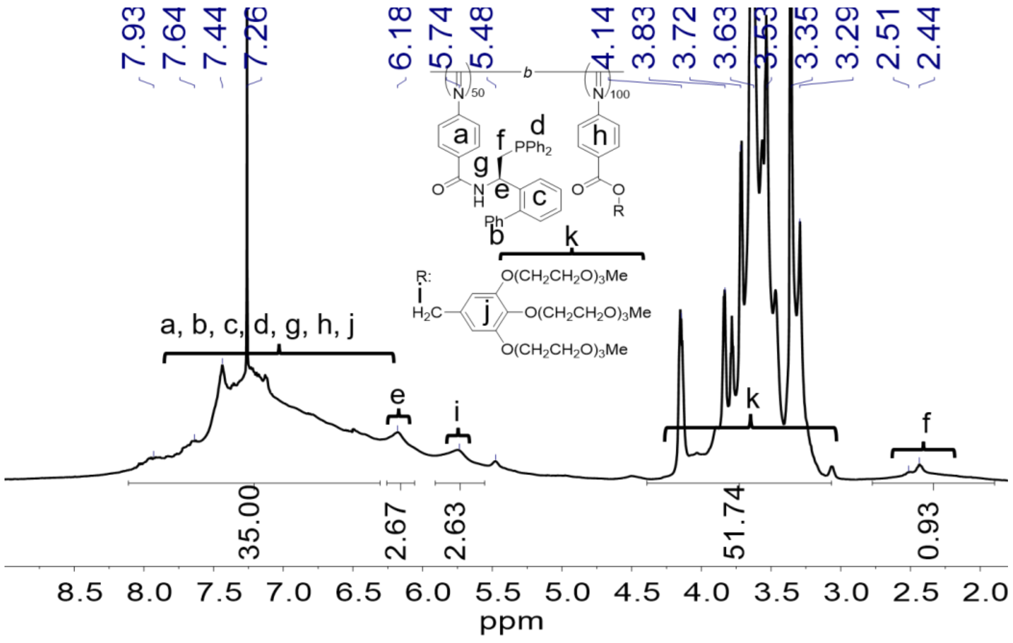

Supplementary Fig. 7. ^{1}H NMR (600 MHz) spectrum of poly(**1r**$_{50}$-*b*-**2**$_{100}$) measured in $CDCl_3$ at 25 °C.

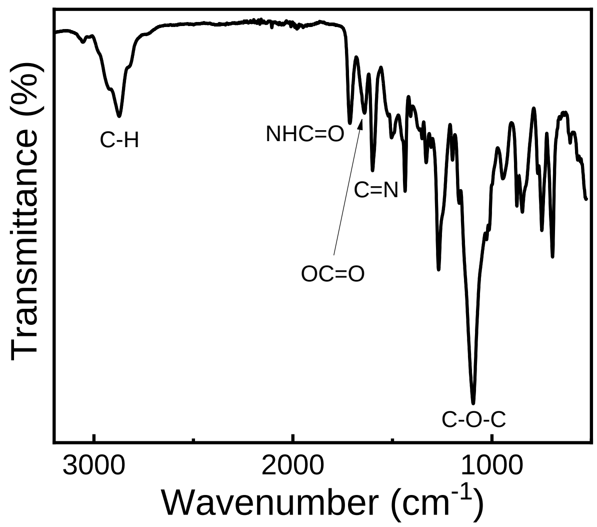

Supplementary Fig. 8. FT-IR spectrum of poly(**1r**$_{50}$-*b*-**2**$_{100}$) measured at 25 °C using KBr pellets.

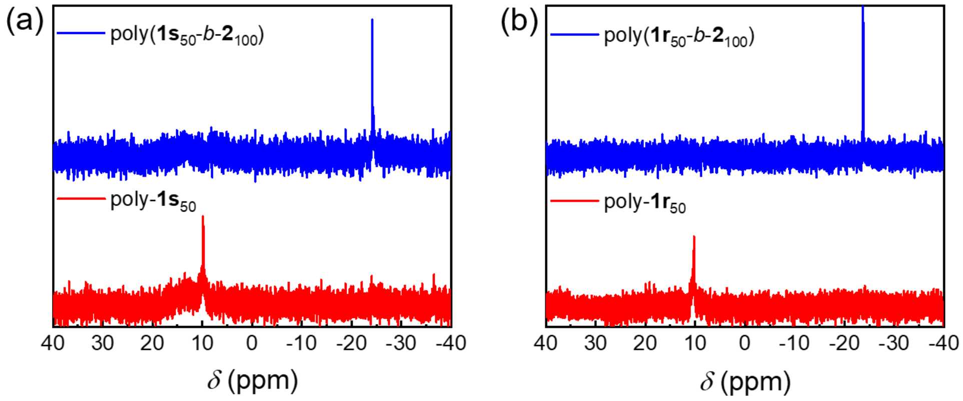

Supplementary Fig. 9. ^{31}P NMR (162 MHz) spectra of (a) poly-**1s**$_{50}$ and poly(**1s**$_{50}$-*b*-**2**$_{100}$), (b) poly-**1r**$_{50}$ and poly(**1r**$_{50}$-*b*-**2**$_{100}$) measured in $CDCl_3$ at 25 °C.

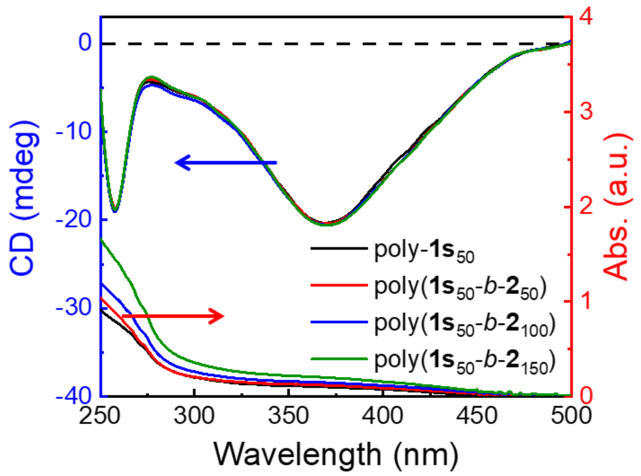

Supplementary Fig. 10. CD and UV-vis spectra of poly-**1s**$_{50}$, poly(**1s**$_{50}$-*b*-**2**$_{50}$), poly(**1s**$_{50}$-*b*-**2**$_{100}$), and poly(**1s**$_{50}$-*b*-**2**$_{150}$) measured in the same concentration of poly-**1s**$_{50}$ block (c = 0.15 mg/mL) at room temperature in THF.

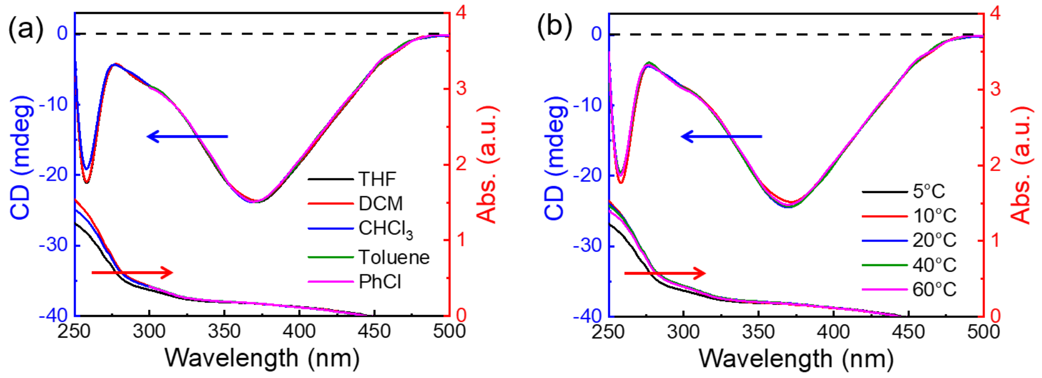

Supplementary Fig. 11. CD and UV-vis spectra of poly(**1s**$_{50}$-*b*-**2**$_{100}$) measured in different solvents at room temperature (a), and in THF at different temperatures (c = 0.2 mg/mL) (b).

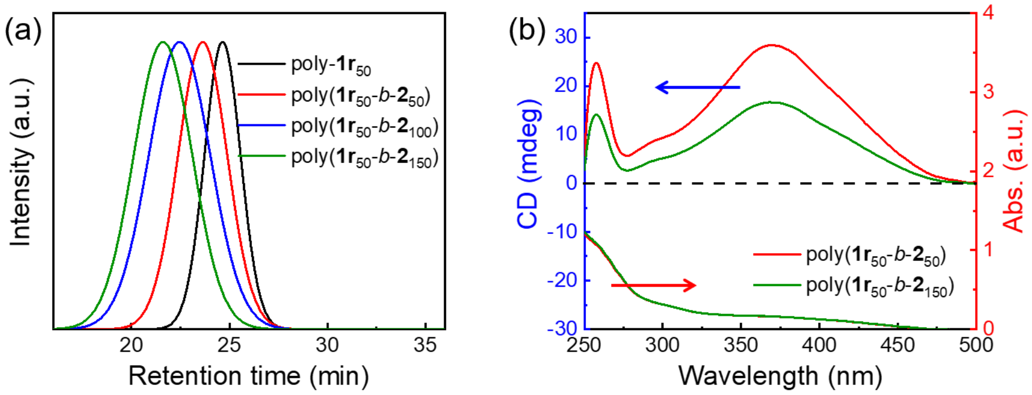

Supplementary Fig. 12. (a) Size exclusion chromatograms of poly-**1r**$_{50}$, poly(**1r**$_{50}$-*b*-**2**$_{50}$), poly(**1r**$_{50}$-*b*-**2**$_{100}$), and poly(**1r**$_{50}$-*b*-**2**$_{150}$) (eluent = THF, temperature = 40 °C). (b) CD and UV-vis spectra of poly(**1r**$_{50}$-*b*-**2**$_{50}$) and poly(**1r**$_{50}$-*b*-**2**$_{150}$) measured in THF at 25 °C (c = 0.2 mg/mL).

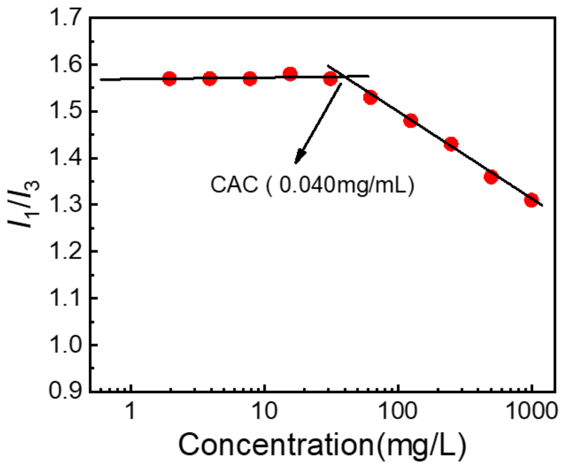

Supplementary Fig. 13. The plots of the emission ratio of I_1/I_3 (I_{373}/I_{384}) as a function of the concentration of poly(**1s**$_{50}$-*b*-**2**$_{100}$) in the presence of pyrene (1.0 μg/mL) in aqueous solutions.

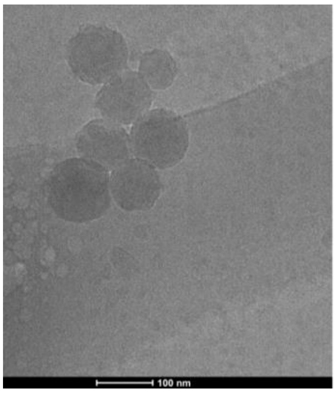

Supplementary Fig. 14. Cryo-TEM images of poly(**1s**$_{50}$-*b*-**2**$_{100}$) casted from the aqueous solution at room temperature.

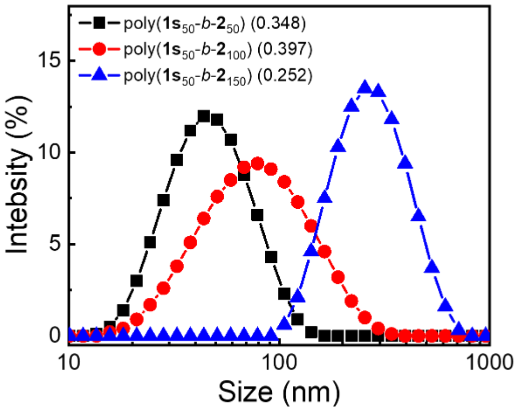

Supplementary Fig. 15. DLS traces for poly(**1s**$_{50}$-*b*-**2**$_{50}$), poly(**1s**$_{50}$-*b*-**2**$_{100}$) and poly(**1s**$_{50}$-*b*-**2**$_{150}$) in H_2O (0.2 mg/mL). The data in the brackets are the polydispersity.

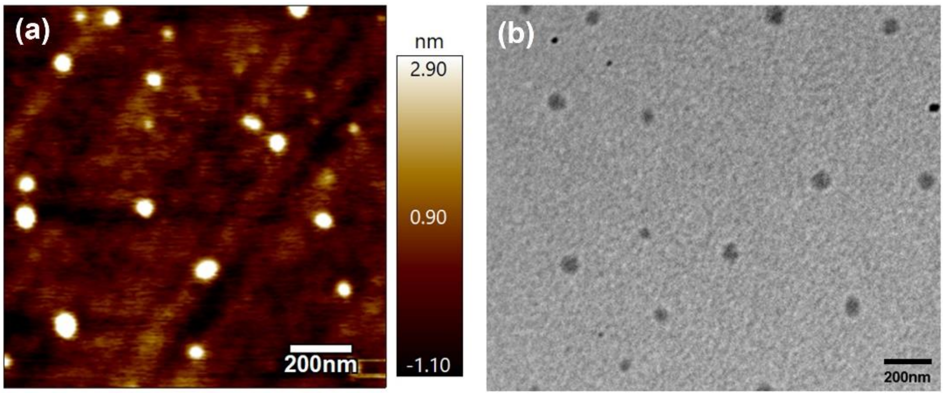

Supplementary Fig. 16. (a) AFM and (b) TEM images of poly(**1r**$_{50}$-*b*-**2**$_{100}$) casted from the aqueous solution at room temperature.

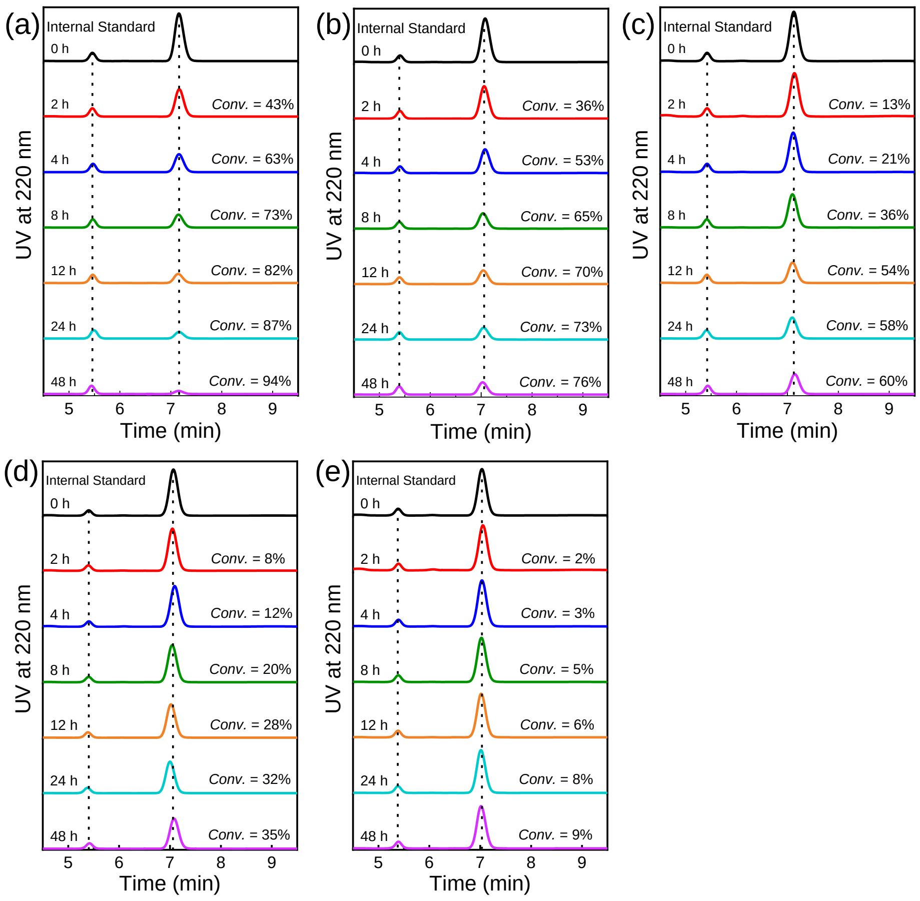

Supplementary Fig. 17. Time-dependent HPLC for the reaction of **3a** and **4a** using 1,4-dimethoxybenzene as internal standard in H_2O/THF with volume ratio of 100/0 (a), 75/25 (b), 50/50 (c), 25/75 (d), and 0/100 (e). HPLC conditions: column: AD-H; eluent: *n*-hexane/isopropanol = 9/1 (v/v); eluent rate: 0.50 mL/min.

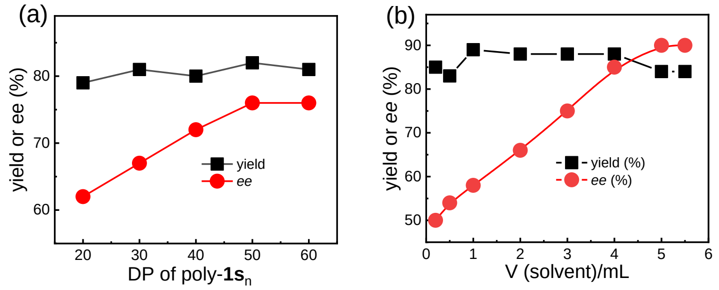

Supplementary Fig. 18. Plots of yield and *ee* values of **5aa** catalyzed by (a) poly-**1s**$_n$ with different degrees of polymerization in $CHCl_3$ at 25 °C, and (b) by poly(**1s**$_{50}$-*b*-**2**$_{100}$) using **3a** (0.1 mmol) and of **4a** (0.3 mmol) in different volumes of water at 25 °C.

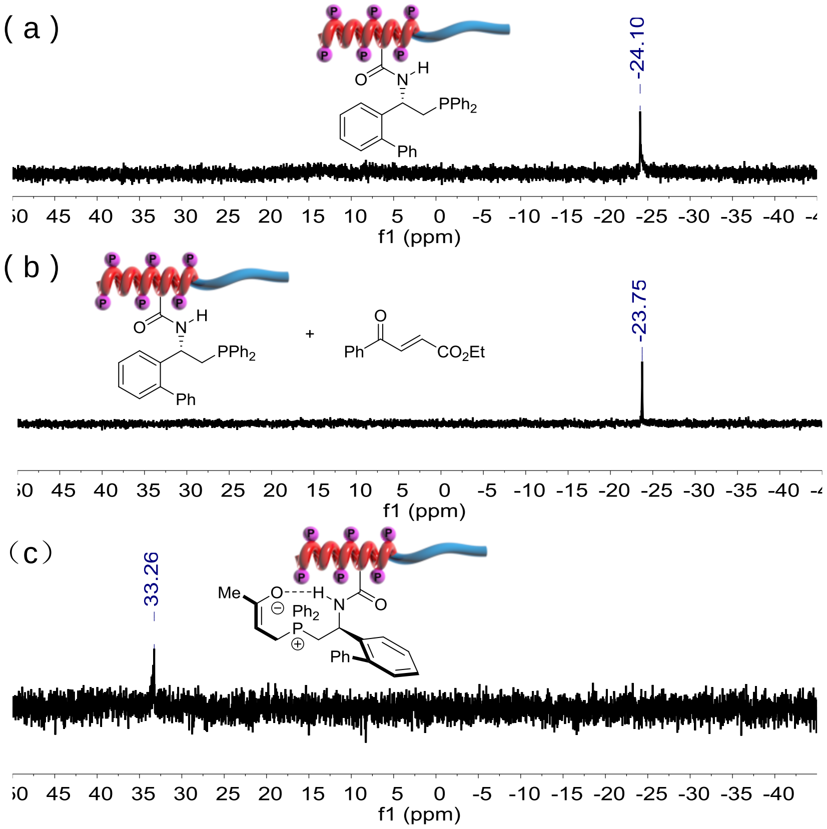

Supplementary Fig. 19. ^{31}P NMR (162 MHz) spectra of (a) poly(**1s**$_{50}$-*b*-**2**$_{50}$); (b) poly(**1s**$_{50}$-*b*-**2**$_{50}$) containing **3a**; (c) poly(**1s**$_{50}$-*b*-**2**$_{50}$) containing **4a** in $CDCl_3$ at 25 °C.

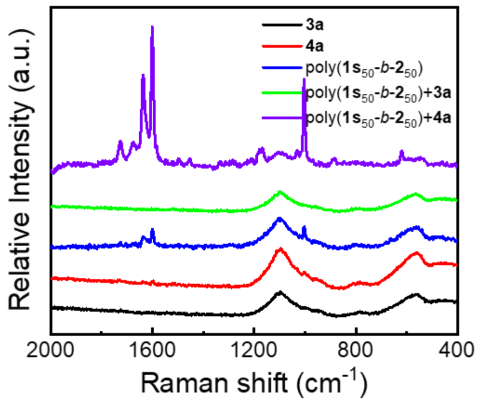

Supplementary Fig. 20. Raman spectra of **3a**, **4a**, poly(**1s**$_{50}$-*b*-**2**$_{50}$), and poly(**1s**$_{50}$-*b*-**2**$_{50}$) with **3a**, and **4a** performed at room temperature excited at 532 nm.

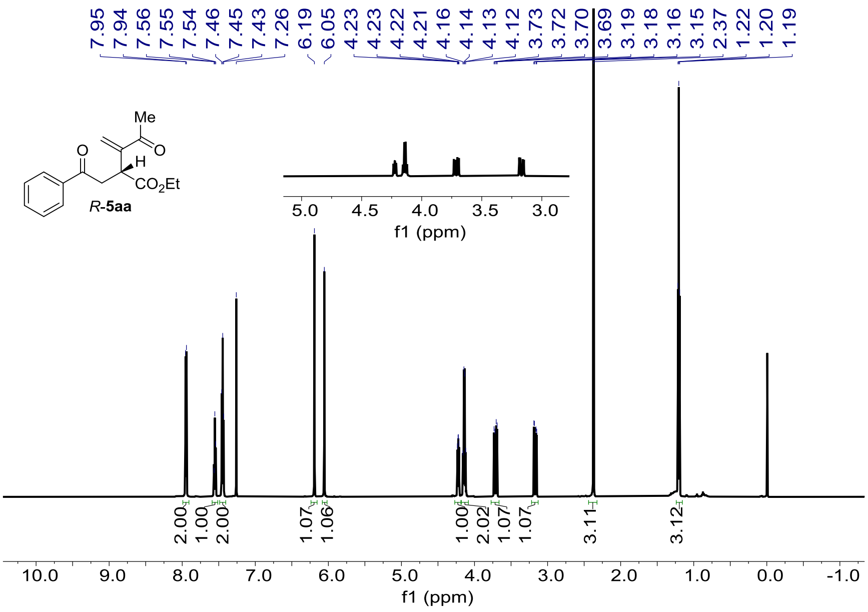

Supplementary Fig. 21. ^{1}H NMR (600 MHz) spectrum of *R*-**5aa** produced by poly(**1s**$_{50}$-*b*-**2**$_{100}$) measured in $CDCl_3$ at 25 °C.

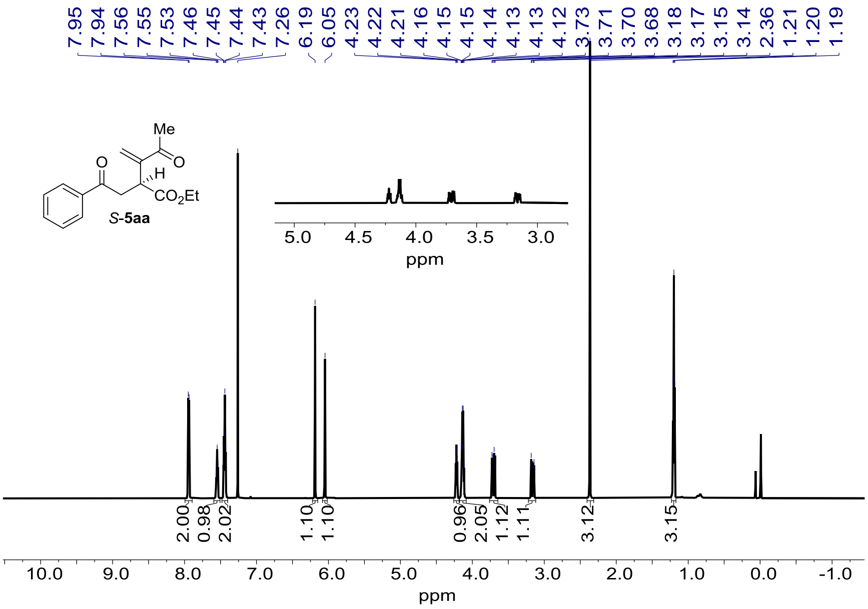

Supplementary Fig. 22. ^{1}H NMR (600 MHz) spectrum of *S*-**5aa** produced by poly(**1r**$_{50}$-*b*-**2**$_{100}$) measured in $CDCl_3$ at 25 °C.

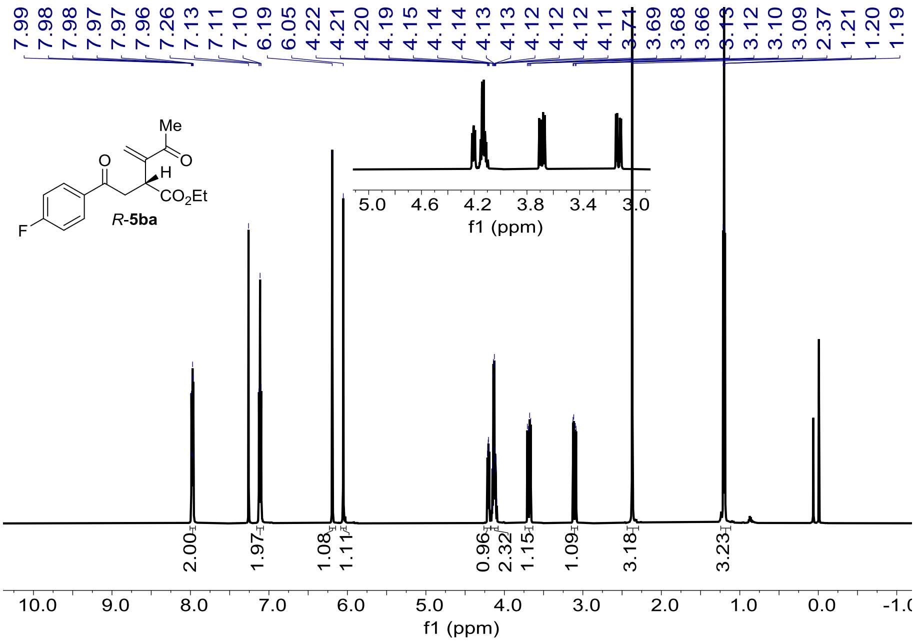

Supplementary Fig. 23. ^{1}H NMR (600 MHz) spectrum of *R*-**5ba** produced by poly(**1s**$_{50}$-*b*-**2**$_{100}$) measured in $CDCl_3$ at 25 °C.

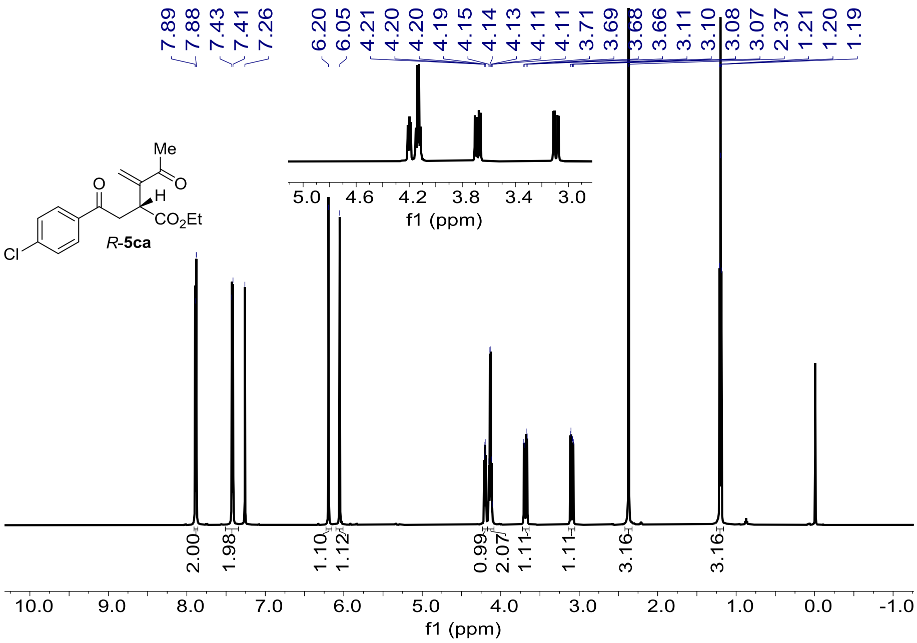

Supplementary Fig. 24. ^{1}H NMR (600 MHz) spectrum of *R*-**5ca** produced by poly(**1s**$_{50}$-*b*-**2**$_{100}$) measured in $CDCl_3$ at 25 °C.

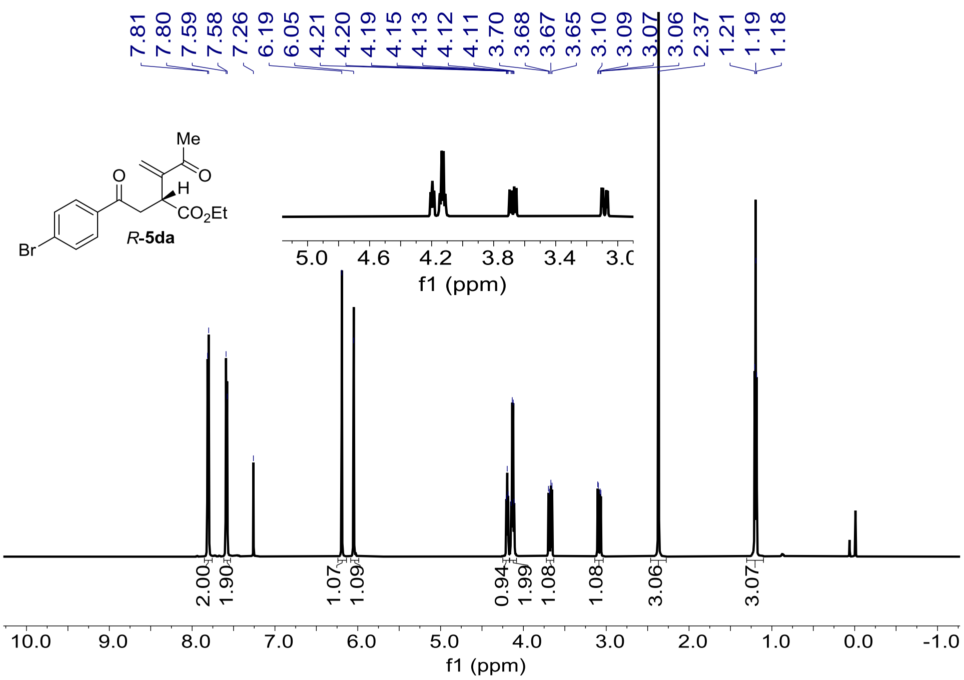

Supplementary Fig. 25. ^{1}H NMR (600 MHz) spectrum of *R*-**5da** produced by poly(**1s**$_{50}$-*b*-**2**$_{100}$) measured in $CDCl_3$ at 25 °C.

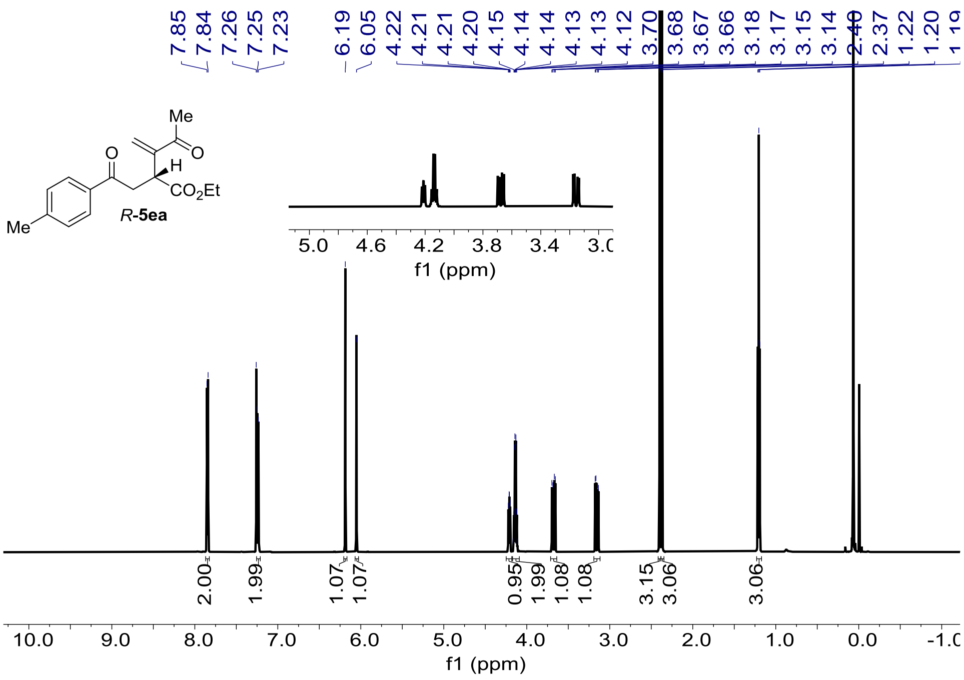

Supplementary Fig. 26. ^{1}H NMR (600 MHz) spectrum of *R*-**5ea** produced by poly(**1s**$_{50}$-*b*-**2**$_{100}$) measured in $CDCl_3$ at 25 °C.

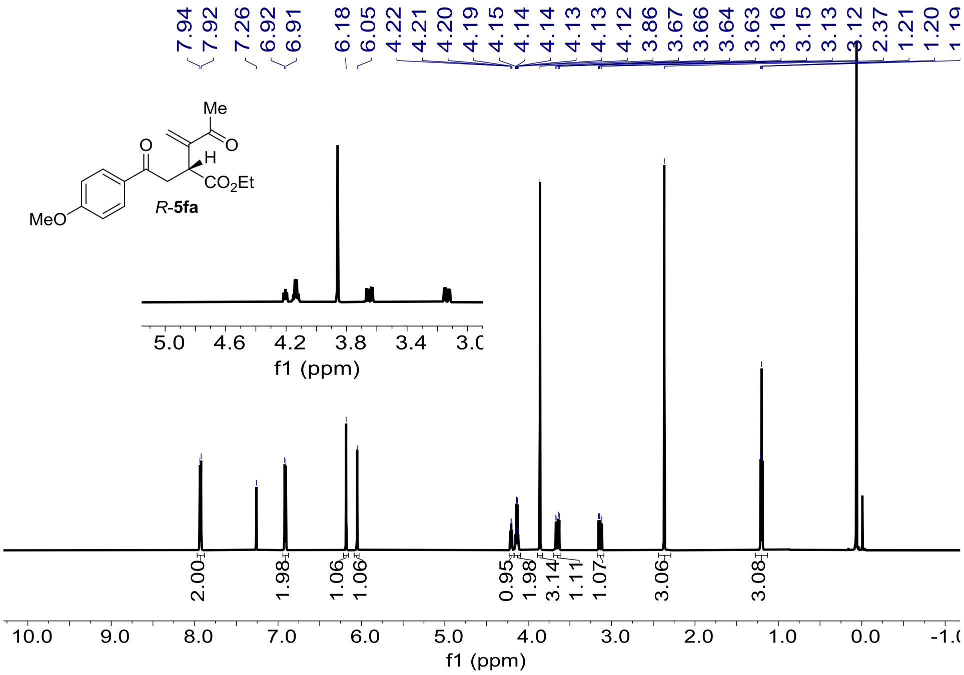

Supplementary Fig. 27. ^{1}H NMR (600 MHz) spectrum of *R*-**5fa** produced by poly(**1s**$_{50}$-*b*-**2**$_{100}$) measured in $CDCl_3$ at 25 °C.

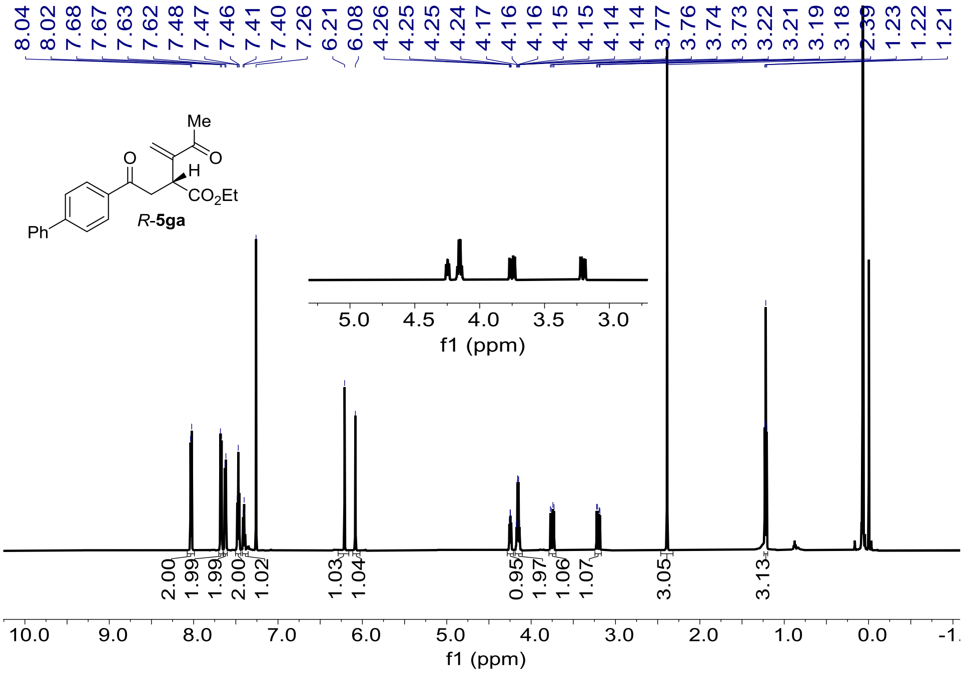

Supplementary Fig. 28. ^{1}H NMR (600 MHz) spectrum of *R*-**5ga** produced by poly(**1s**$_{50}$-*b*-**2**$_{100}$) measured in $CDCl_3$ at 25 °C.

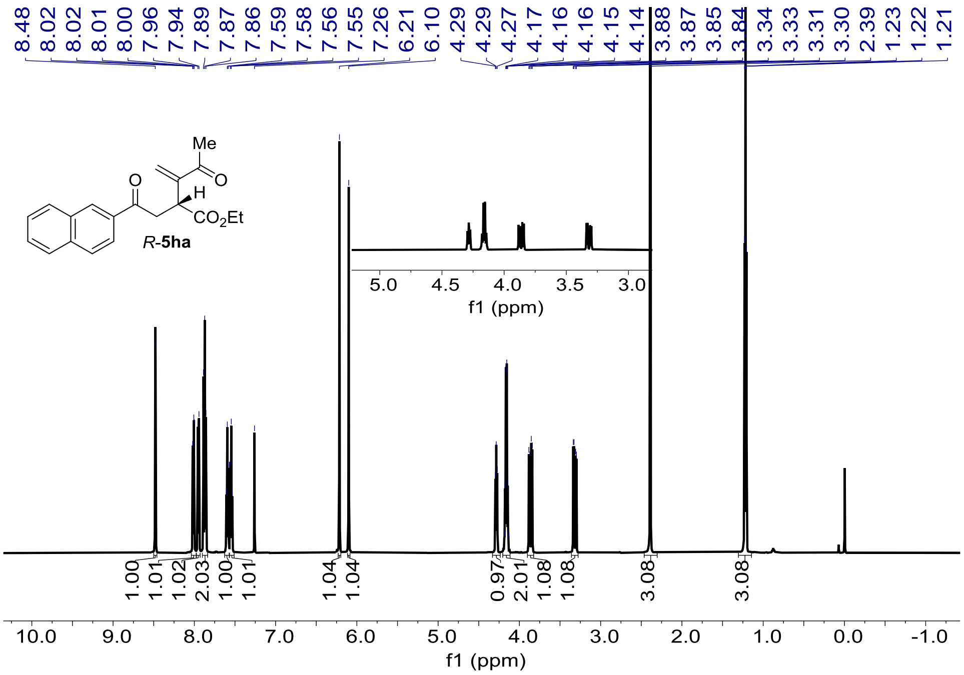

Supplementary Fig. 29. ^{1}H NMR (600 MHz) spectrum of *R*-**5ha** produced by poly(**1s**$_{50}$-*b*-**2**$_{100}$) measured in $CDCl_3$ at 25 °C.

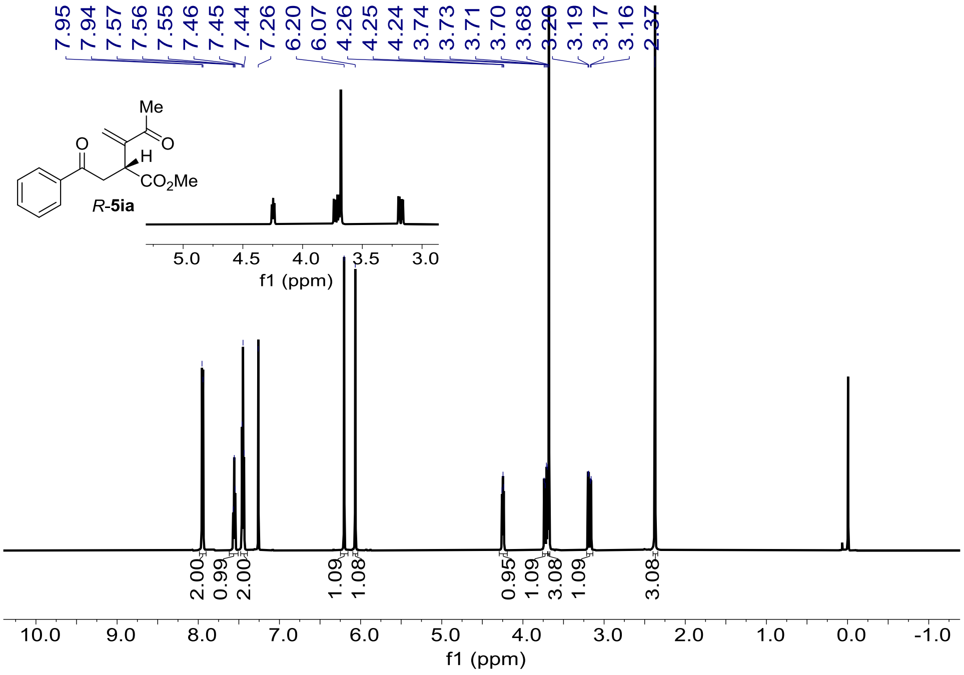

Supplementary Fig. 30. ^{1}H NMR (600 MHz) spectrum of *R*-**5ia** produced by poly(**1s**$_{50}$-*b*-**2**$_{100}$) measured in $CDCl_3$ at 25 °C.

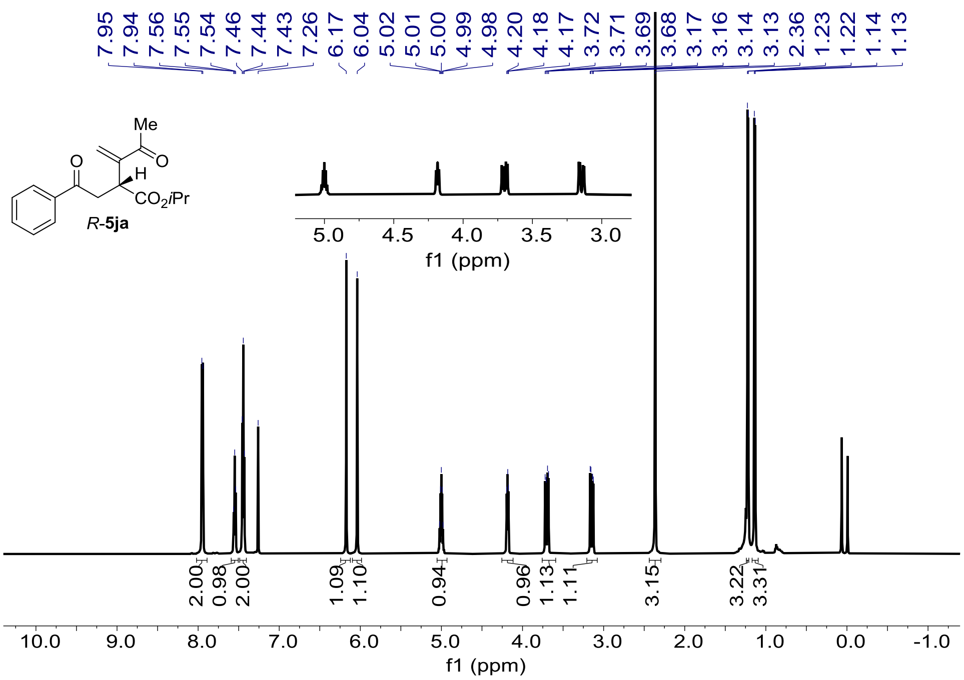

Supplementary Fig. 31. ^{1}H NMR (600 MHz) spectrum of *R*-**5ja** produced by poly(**1s**$_{50}$-*b*-**2**$_{100}$) measured in $CDCl_3$ at 25 °C.

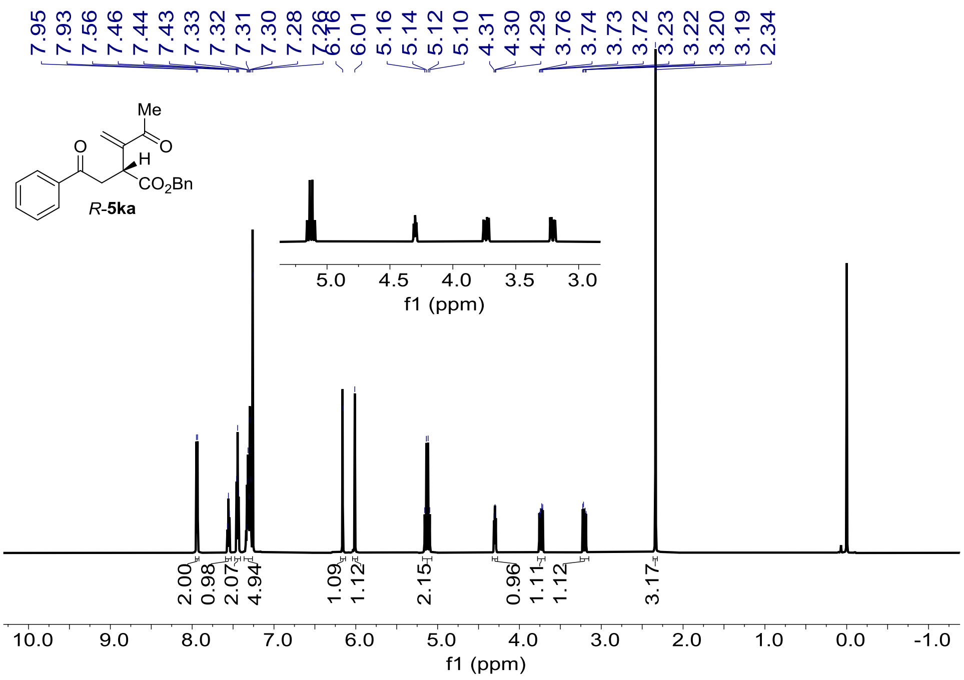

Supplementary Fig. 32. ^{1}H NMR (600 MHz) spectrum of *R*-**5ka** produced by poly(**1s**$_{50}$-*b*-**2**$_{100}$) measured in $CDCl_3$ at 25 °C.

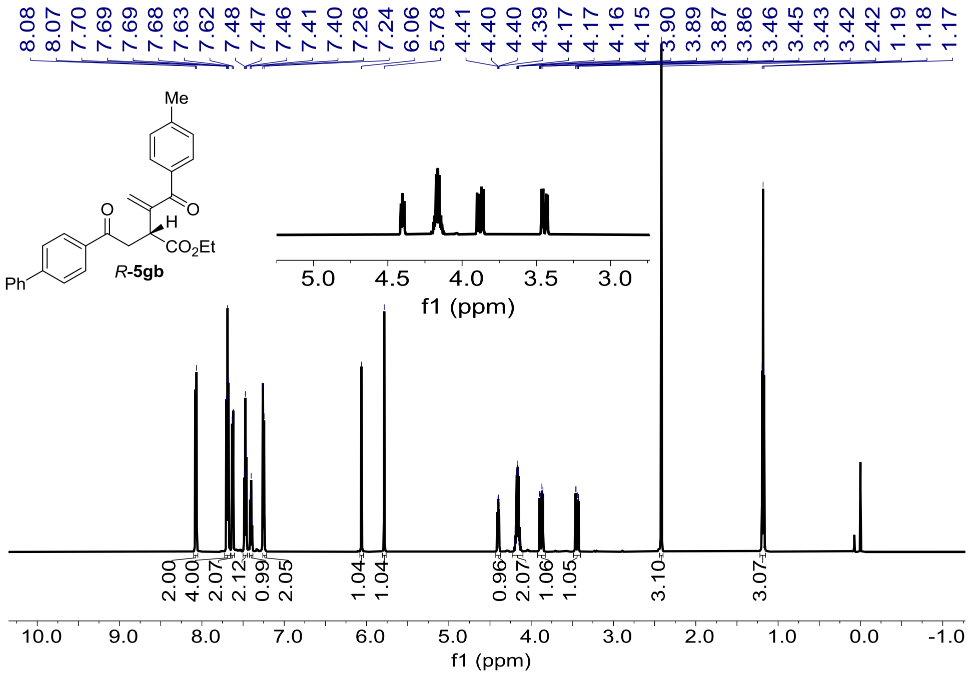

Supplementary Fig. 33. ^{1}H NMR (600 MHz) spectrum of *R*-**5gb** produced by poly(**1s**$_{50}$-*b*-**2**$_{100}$) measured in $CDCl_3$ at 25 °C.

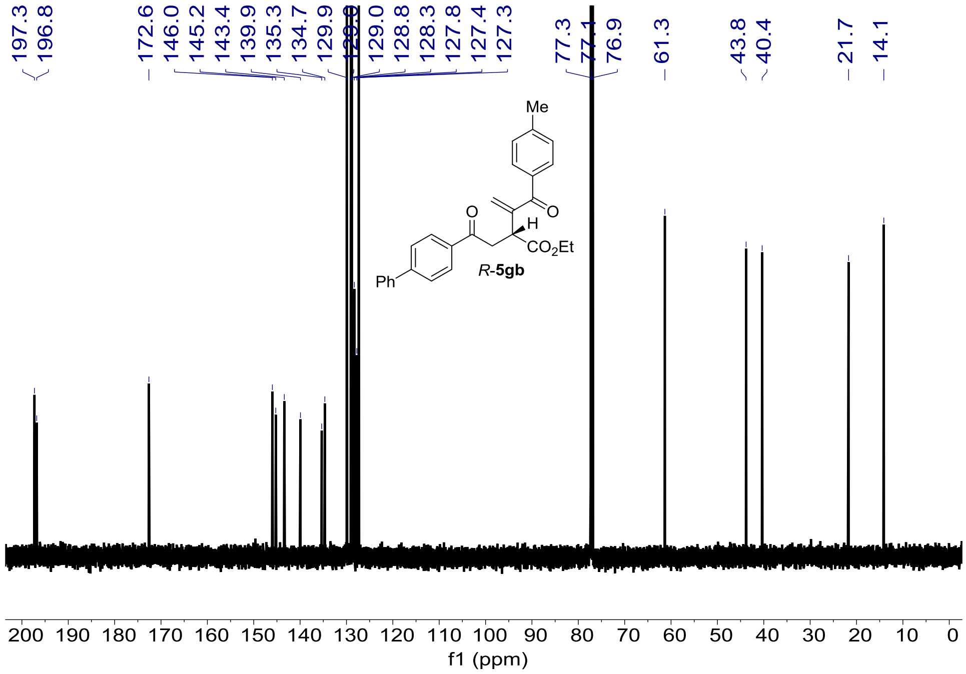

Supplementary Fig. 34. ^{13}C NMR (150 MHz) spectrum of *R*-**5gb** produced by poly(**1s**$_{50}$-*b*-**2**$_{100}$) measured in $CDCl_3$ at 25 °C.

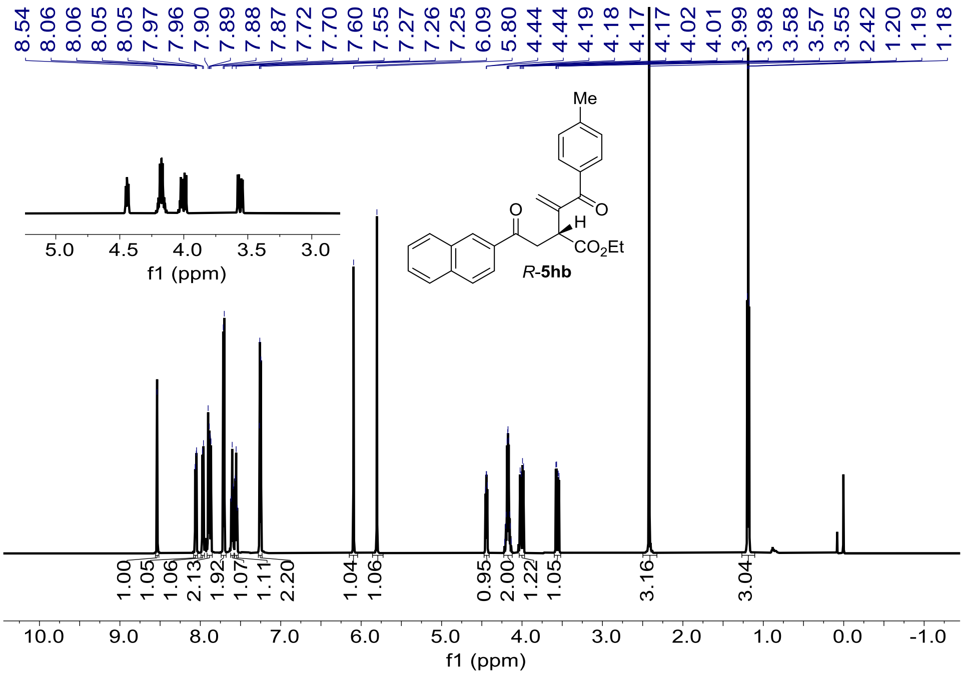

Supplementary Fig. 35. ^{1}H NMR (600 MHz) spectrum of ***R*-5hb** produced by poly(**1s**$_{50}$-*b*-**2**$_{100}$) measured in $CDCl_3$ at 25 °C.

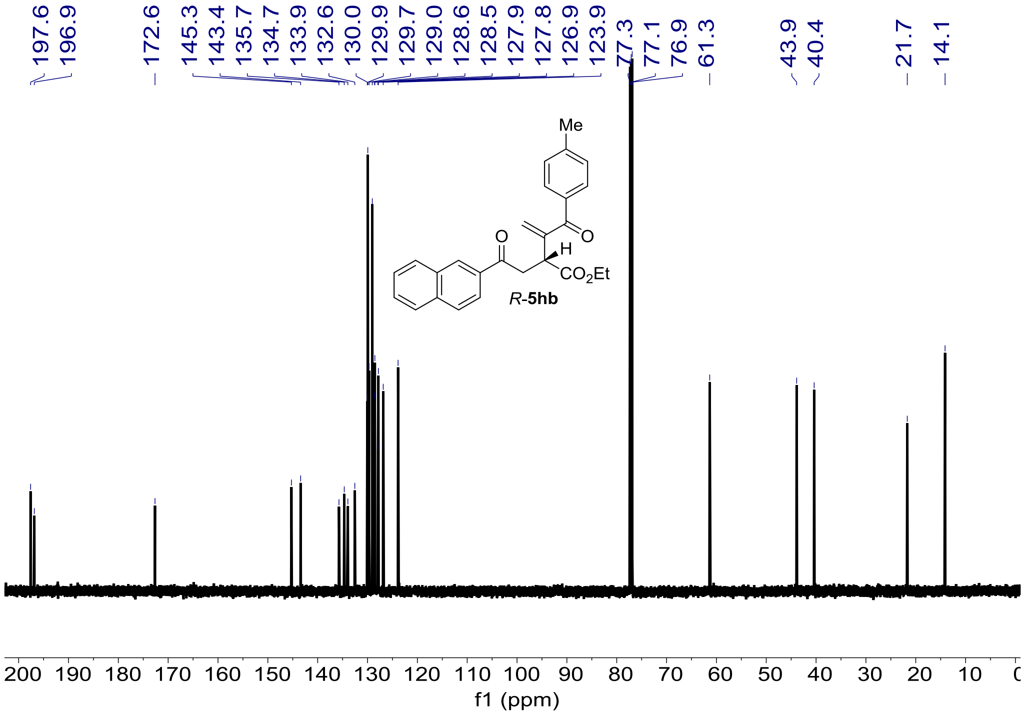

Supplementary Fig. 36. ^{13}C NMR (150 MHz) spectrum of ***R*-5hb** produced by poly(**1s**$_{50}$-*b*-**2**$_{100}$) measured in $CDCl_3$ at 25 °C.

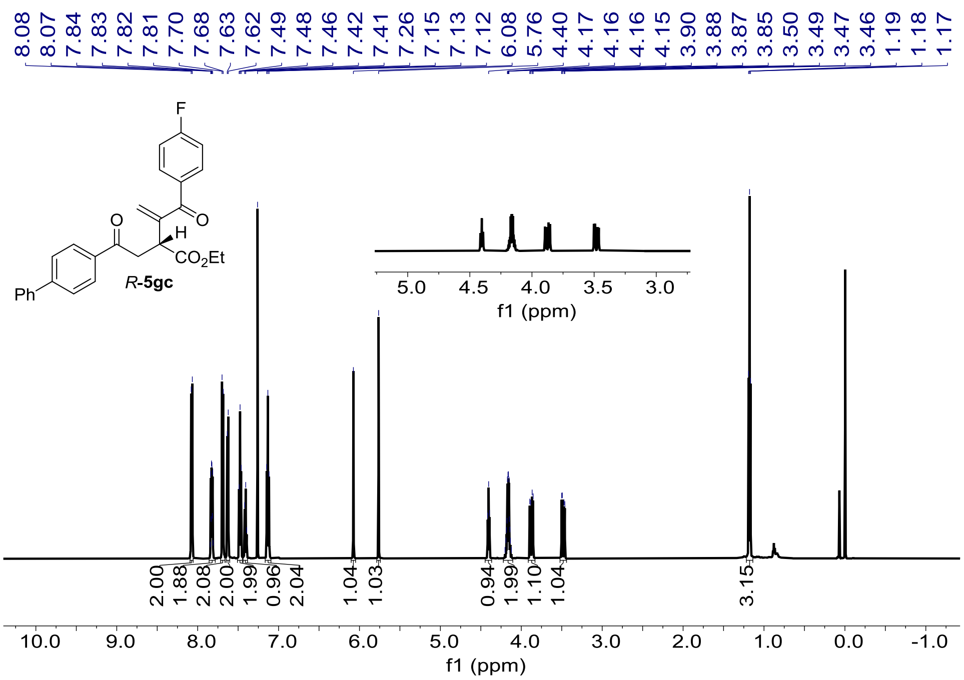

Supplementary Fig. 37. ^{1}H NMR (600 MHz) spectrum of *R*-**5gc** produced by poly(**1s**$_{50}$-*b*-**2**$_{100}$) measured in $CDCl_3$ at 25 °C.

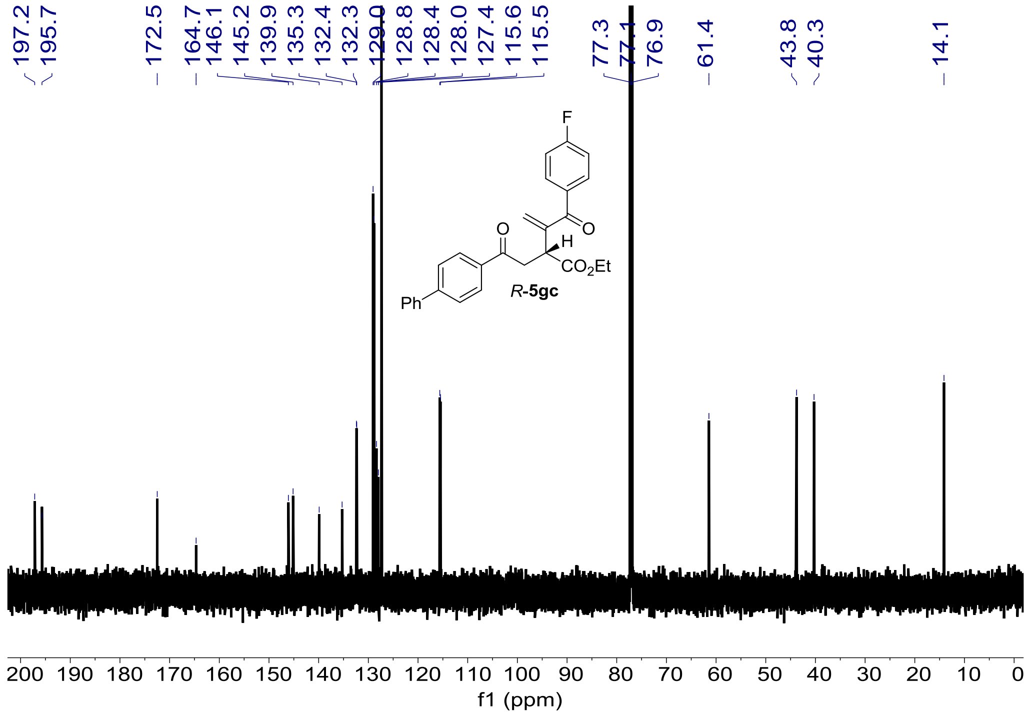

Supplementary Fig. 38. ^{13}C NMR (150 MHz) spectrum of *R*-**5gc** produced by poly(**1s**$_{50}$-*b*-**2**$_{100}$) measured in $CDCl_3$ at 25 °C.

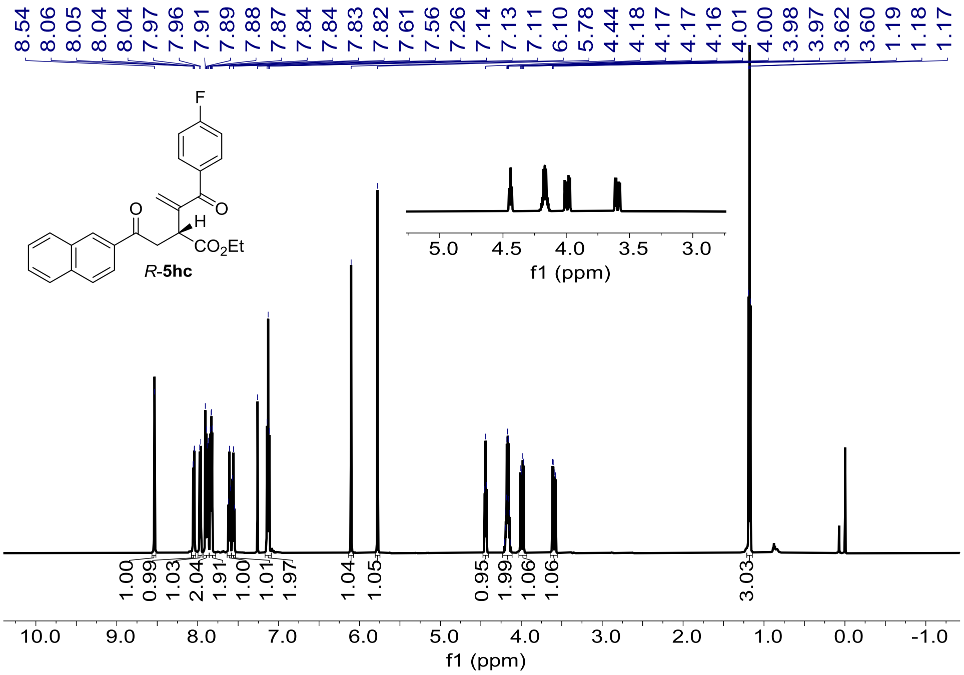

Supplementary Fig. 39. ^{1}H NMR (600 MHz) spectrum of *R*-**5hc** produced by poly(**1s**$_{50}$-*b*-**2**$_{100}$) measured in $CDCl_3$ at 25 °C.

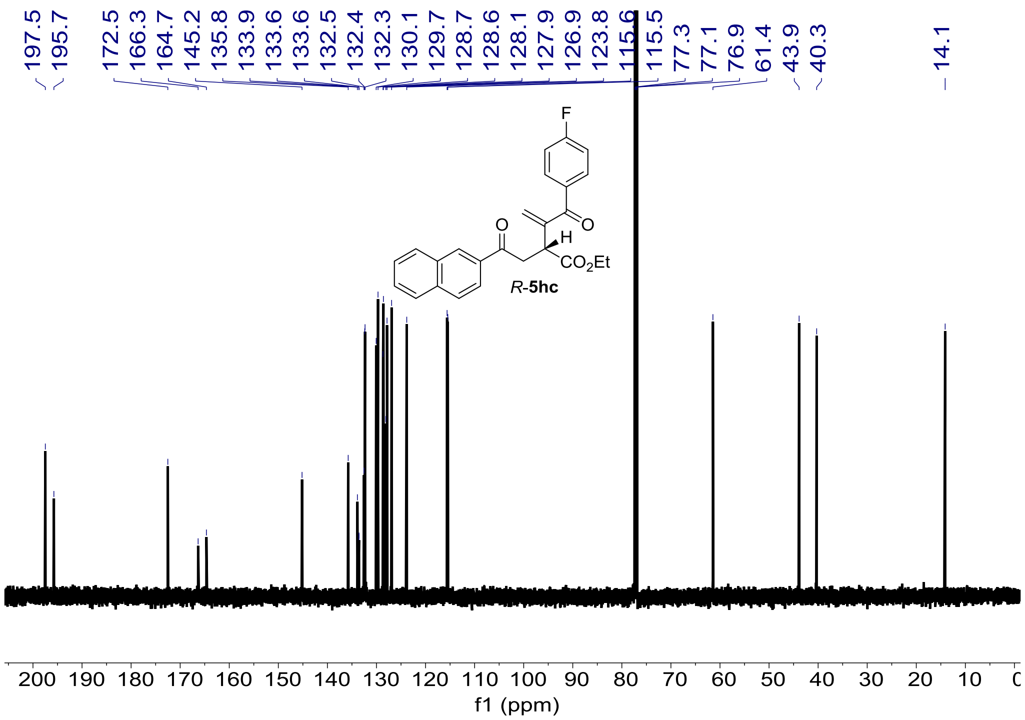

Supplementary Fig. 40. ^{13}C NMR (150 MHz) spectrum of *R*-**5hc** produced by poly(**1s**$_{50}$-*b*-**2**$_{100}$) measured in $CDCl_3$ at 25 °C.

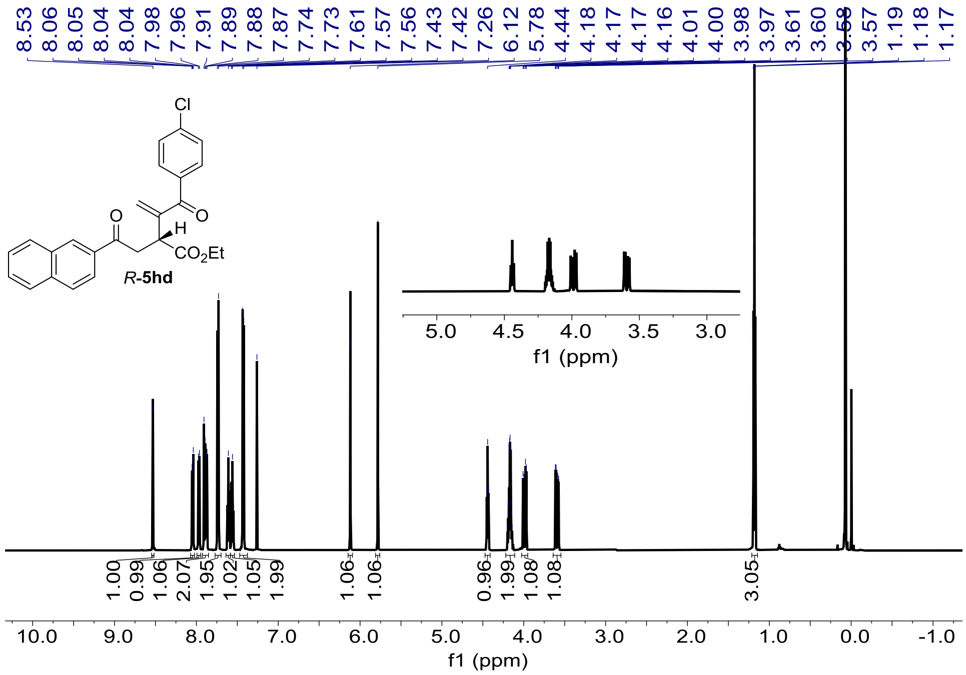

Supplementary Fig. 41. ^{1}H NMR (600 MHz) spectrum of ***R*-5hd** produced by poly(**1s**$_{50}$-*b*-**2**$_{100}$) measured in $CDCl_3$ at 25 °C.

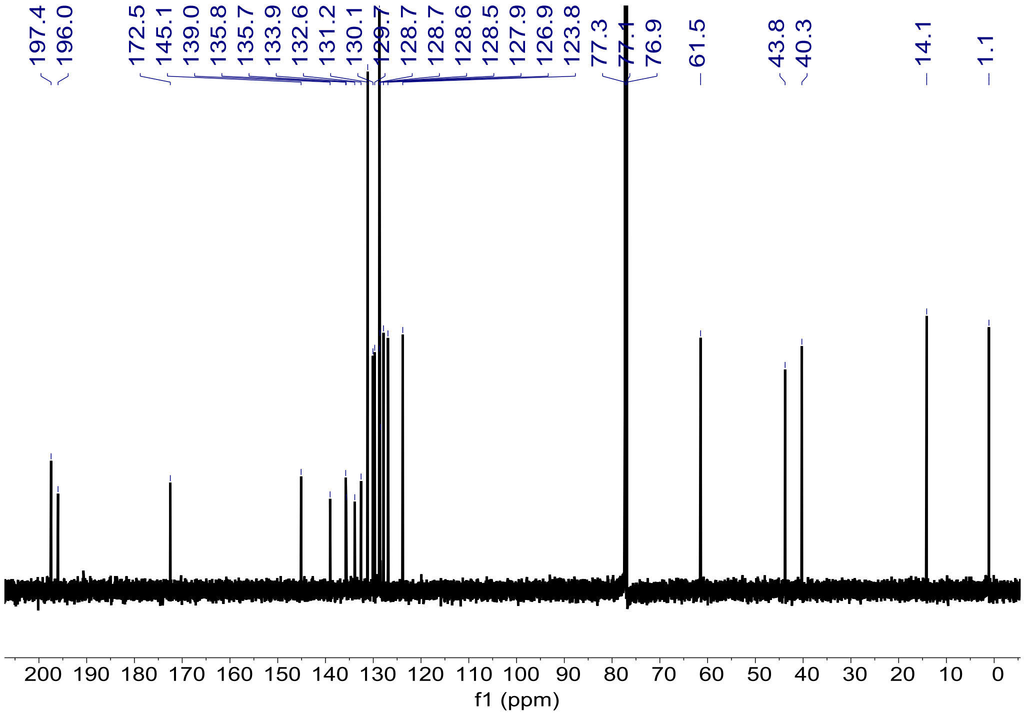

Supplementary Fig. 42. ^{13}C NMR (150 MHz) spectrum of ***R*-5hd** produced by poly(**1s**$_{50}$-*b*-**2**$_{100}$) measured in $CDCl_3$ at 25 °C.

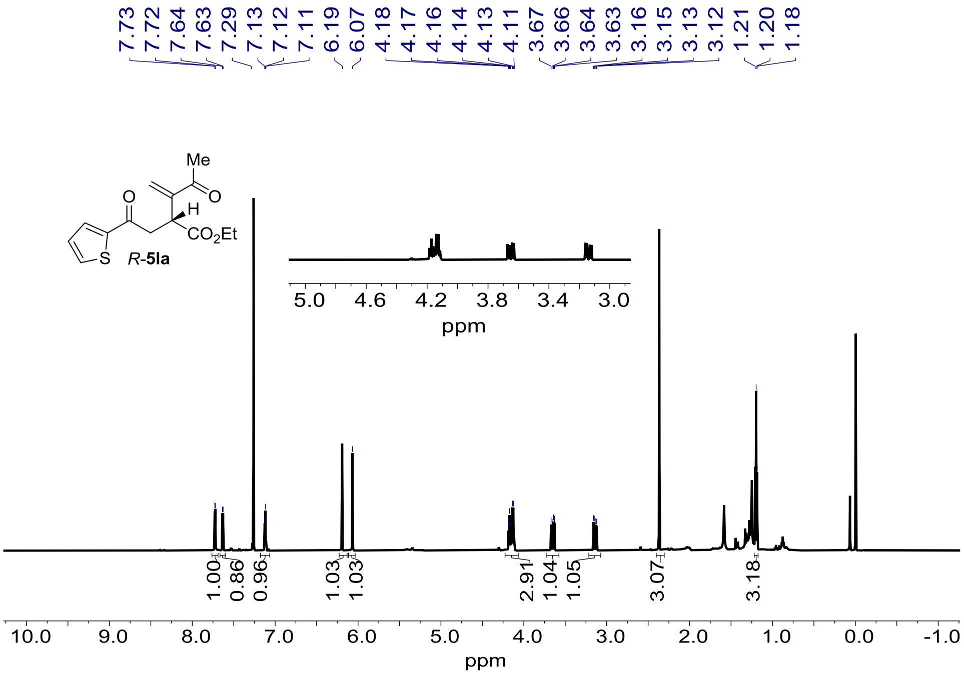

Supplementary Fig. 43. ^{1}H NMR (600 MHz) spectrum of *R*-**5la** produced by poly(**1s**$_{50}$-*b*-**2**$_{100}$) measured in $CDCl_3$ at 25 °C.

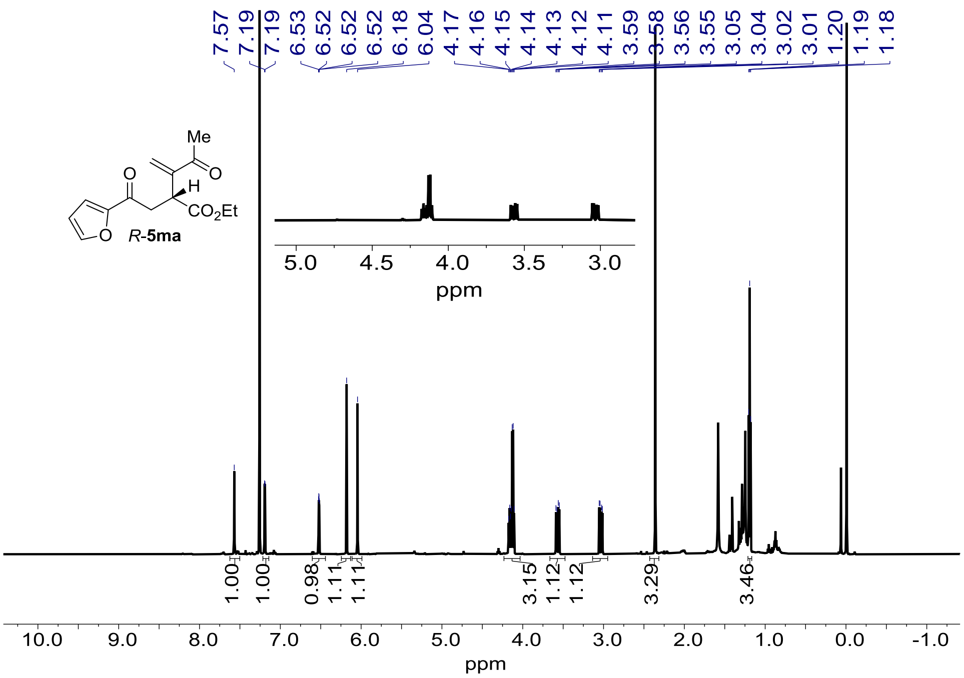

Supplementary Fig. 44. ^{1}H NMR (600 MHz) spectrum of *R*-**5ma** produced by poly(**1s**$_{50}$-*b*-**2**$_{100}$) measured in $CDCl_3$ at 25 °C.

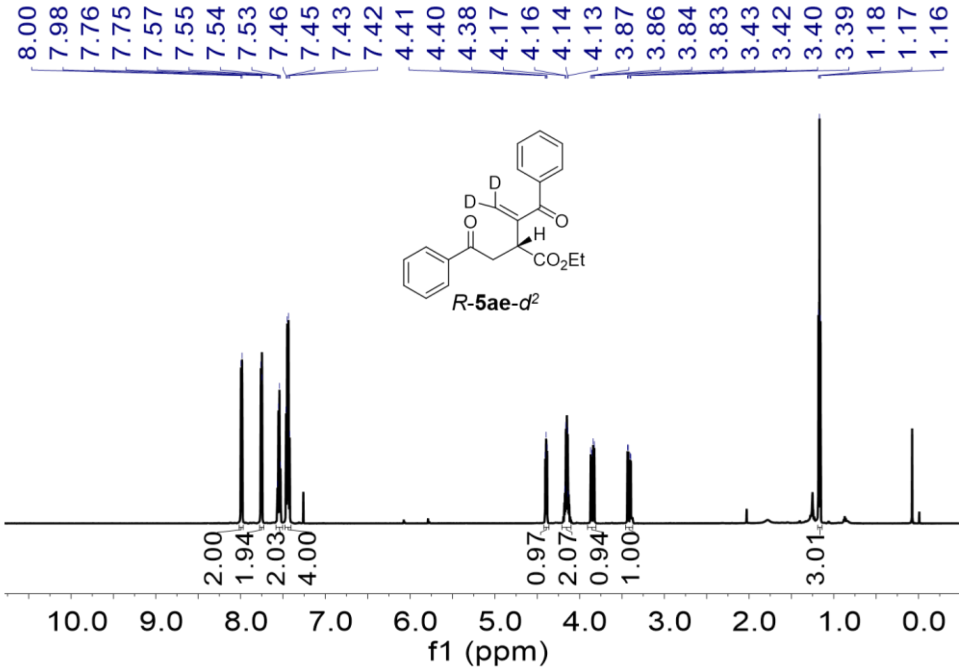

Supplementary Fig. 45. ^{1}H NMR (600 MHz) spectrum of *R*-**5ae**-d^2 produced by poly(**1s**$_{50}$-*b*-**2**$_{100}$) measured in $CDCl_3$ at 25 °C.

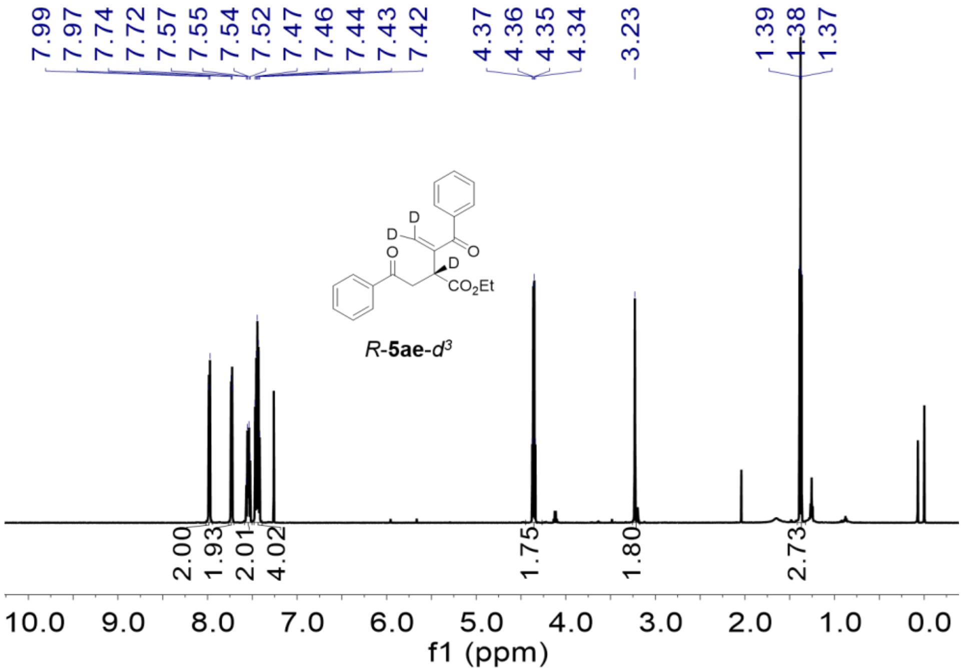

Supplementary Fig. 46. ^{1}H NMR (600 MHz) spectrum of *R*-**5ae**-d^3 produced by poly(**1s**$_{50}$-*b*-**2**$_{100}$) measured in $CDCl_3$ at 25 °C.

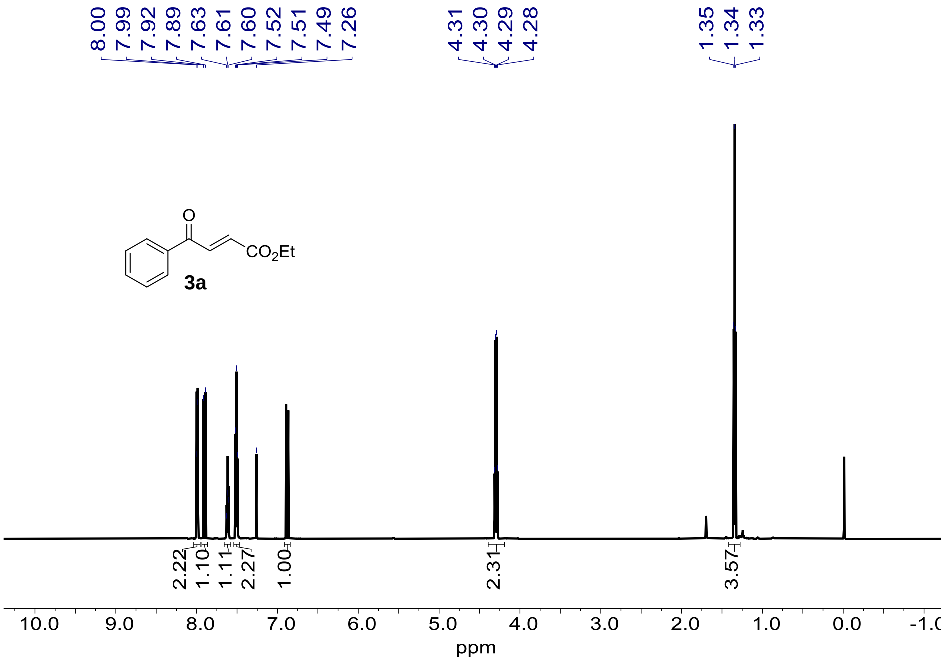

Supplementary Fig. 47. ^{1}H NMR (600 MHz) spectrum of **3a** measured in $CDCl_3$ at 25 °C.

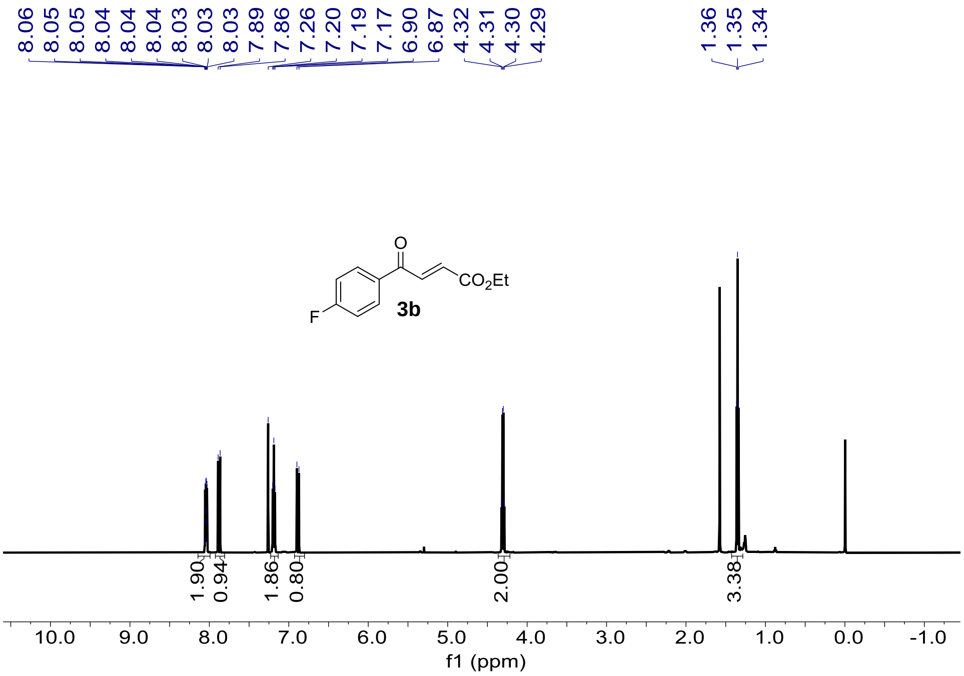

Supplementary Fig. 48. ^{1}H NMR (600 MHz) spectrum of **3b** measured in $CDCl_3$ at 25 °C.

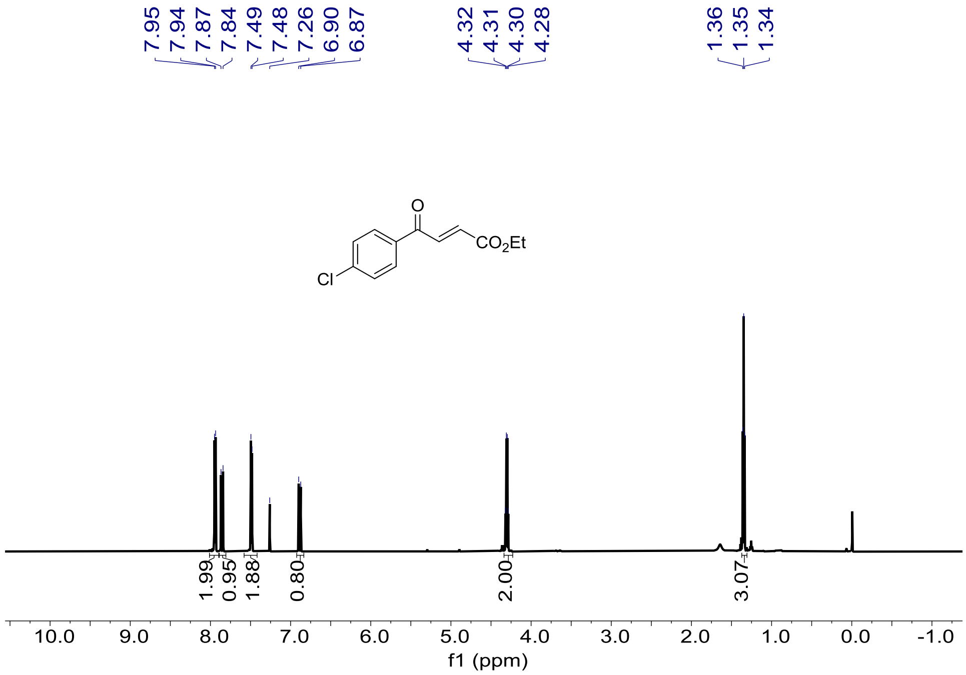

Supplementary Fig. 49. ^{1}H NMR (600 MHz) spectrum of **3c** measured in $CDCl_3$ at 25 °C.

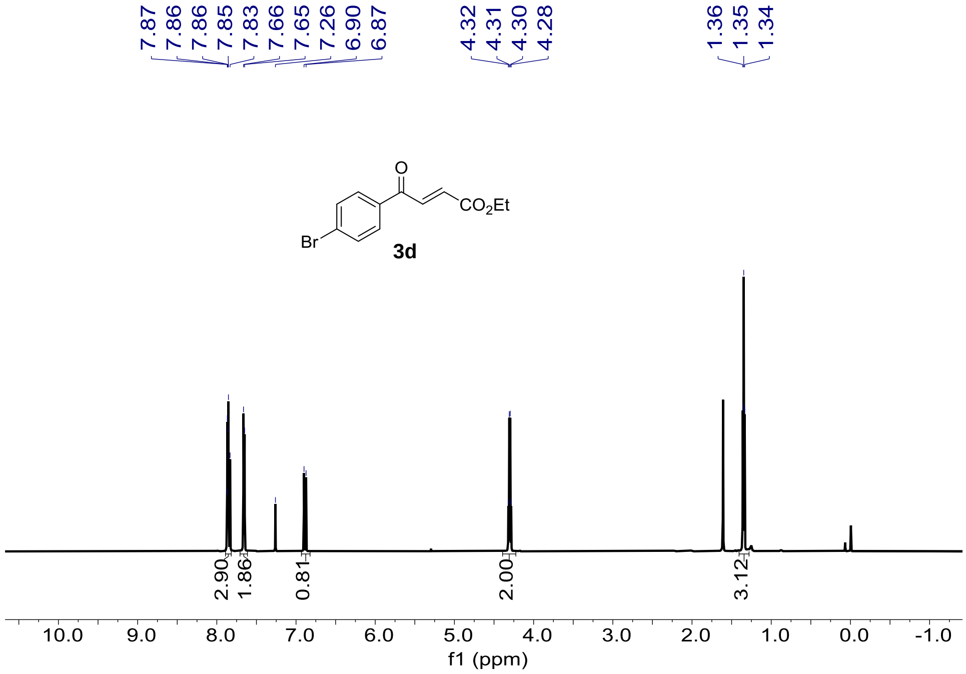

Supplementary Fig. 50. ^{1}H NMR (600 MHz) spectrum of **3d** measured in $CDCl_3$ at 25 °C.

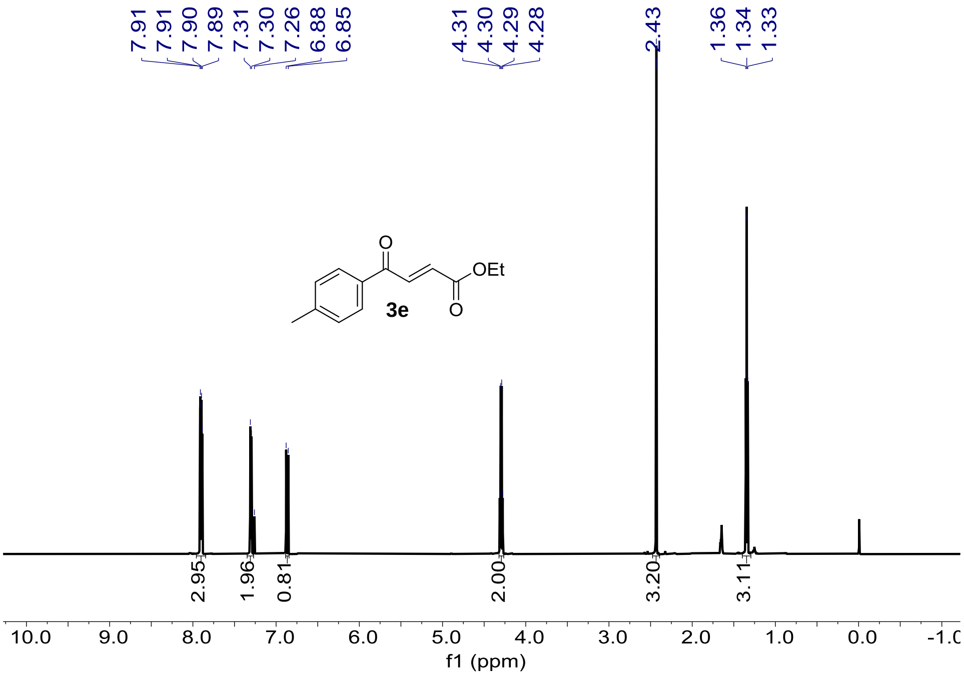

Supplementary Fig. 51. ^{1}H NMR (600 MHz) spectrum of **3e** measured in $CDCl_3$ at 25 °C.

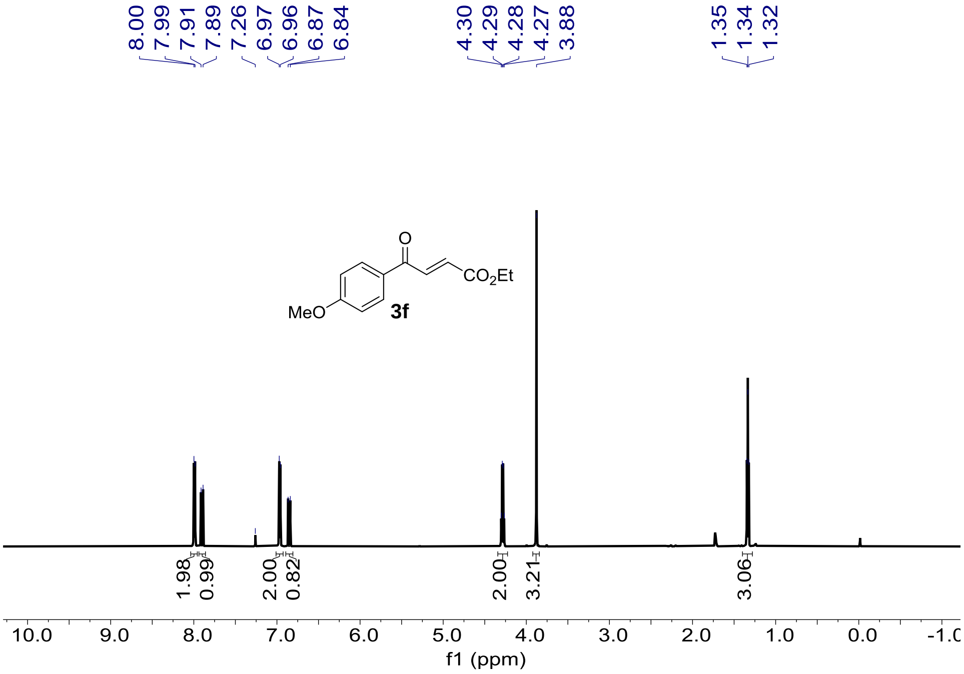

Supplementary Fig. 52. ^{1}H NMR (600 MHz) spectrum of **3f** measured in $CDCl_3$ at 25 °C.

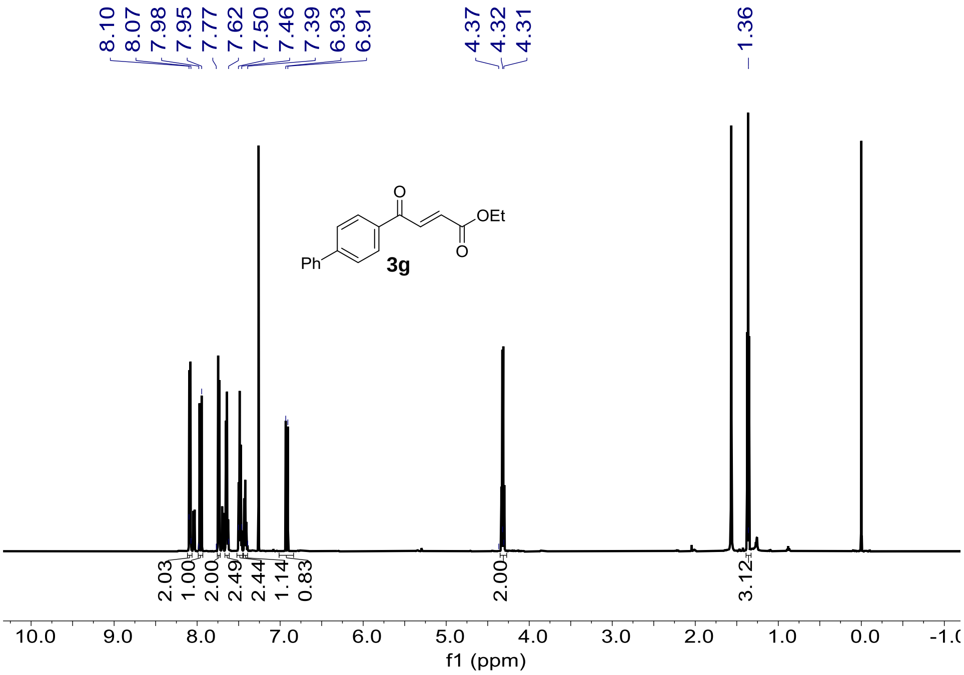

Supplementary Fig. 53. ^{1}H NMR (600 MHz) spectrum of **3g** measured in $CDCl_3$ at 25 °C.

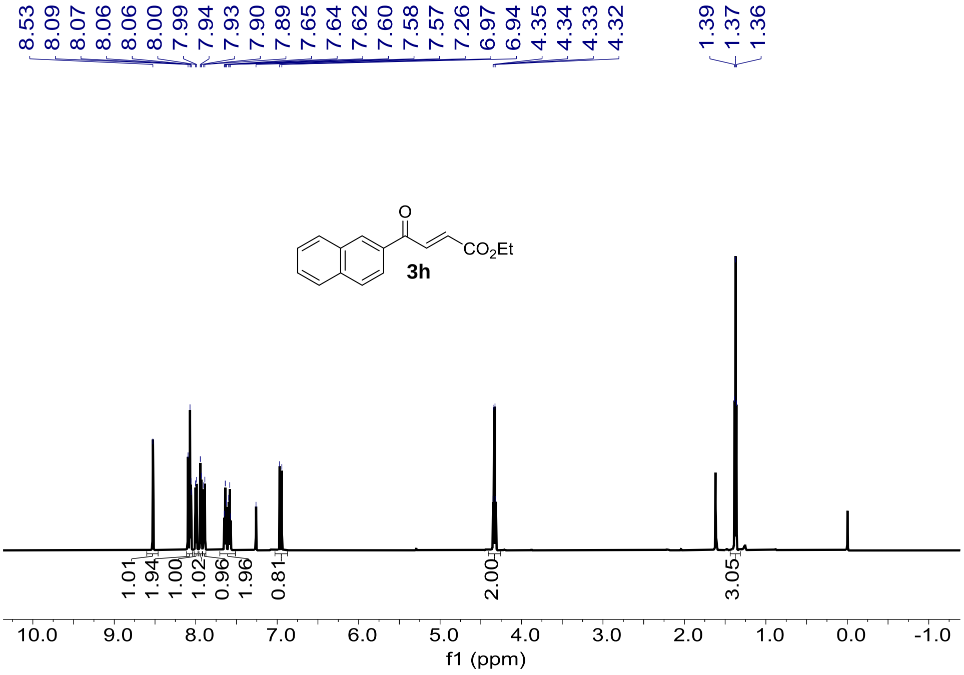

Supplementary Fig. 54. ^{1}H NMR (600 MHz) spectrum of **3h** measured in $CDCl_3$ at 25 °C.

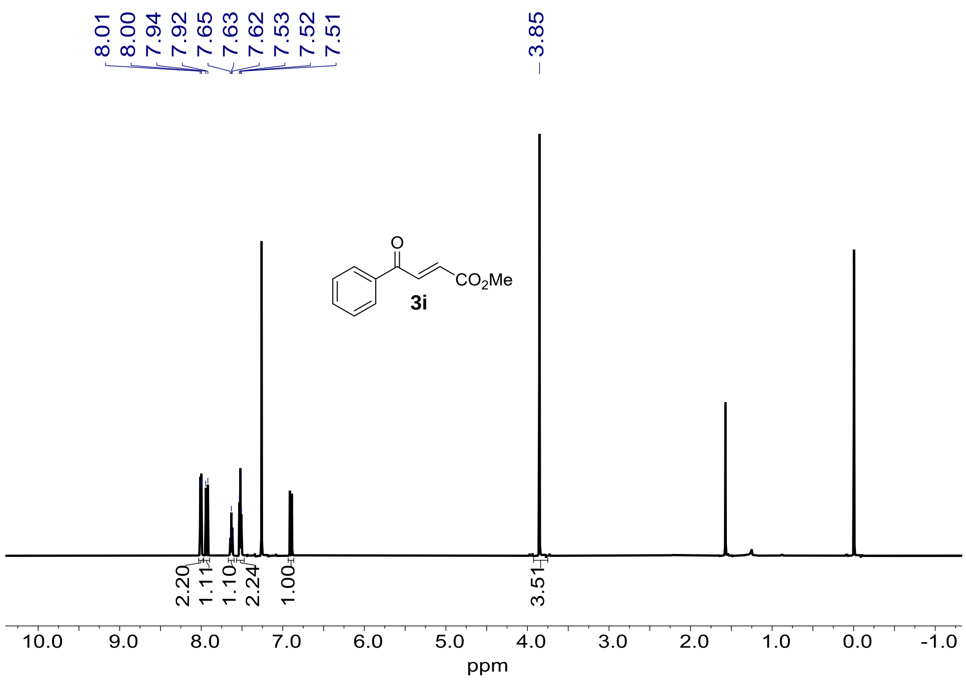

Supplementary Fig. 55. ^{1}H NMR (600 MHz) spectrum of **3i** measured in $CDCl_3$ at 25 °C.

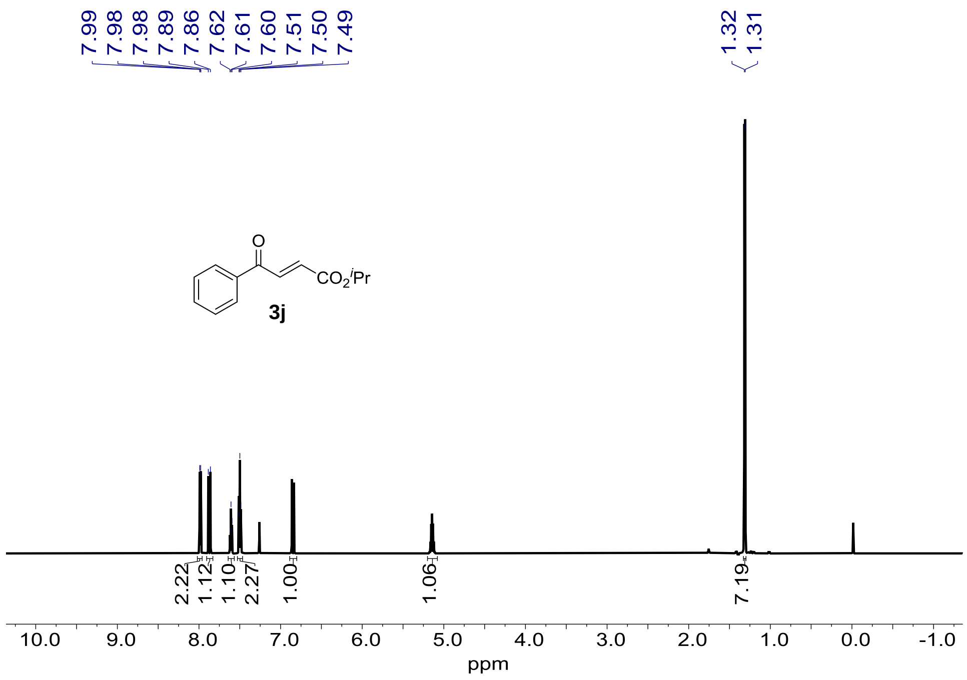

Supplementary Fig. 56. ^{1}H NMR (600 MHz) spectrum of **3j** measured in $CDCl_3$ at 25 °C.

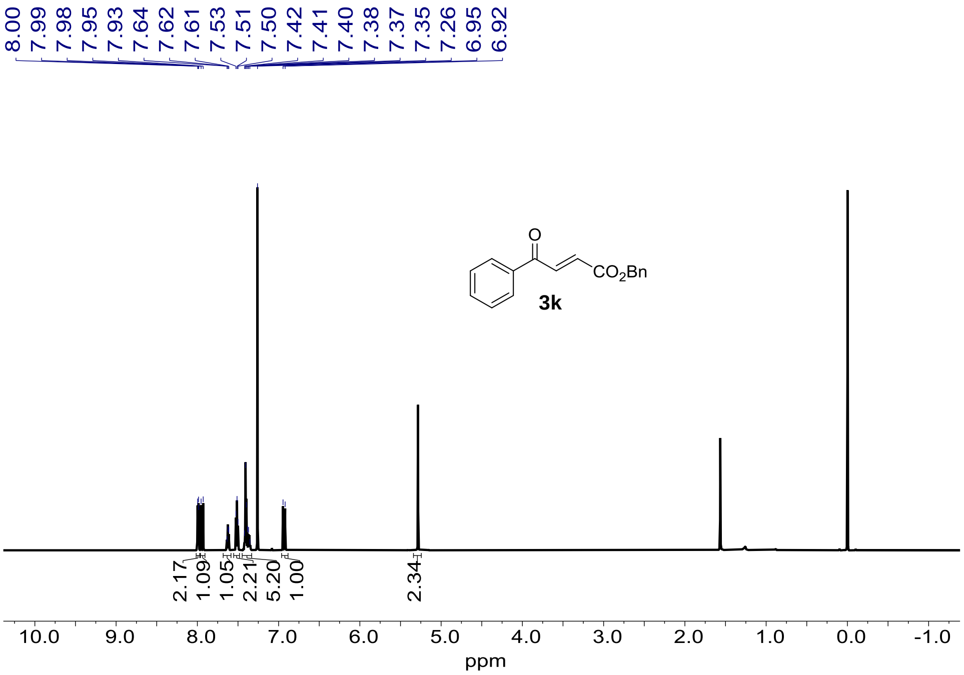

Supplementary Fig. 57. ^{1}H NMR (600 MHz) spectrum of **3k** measured in $CDCl_3$ at 25 °C.

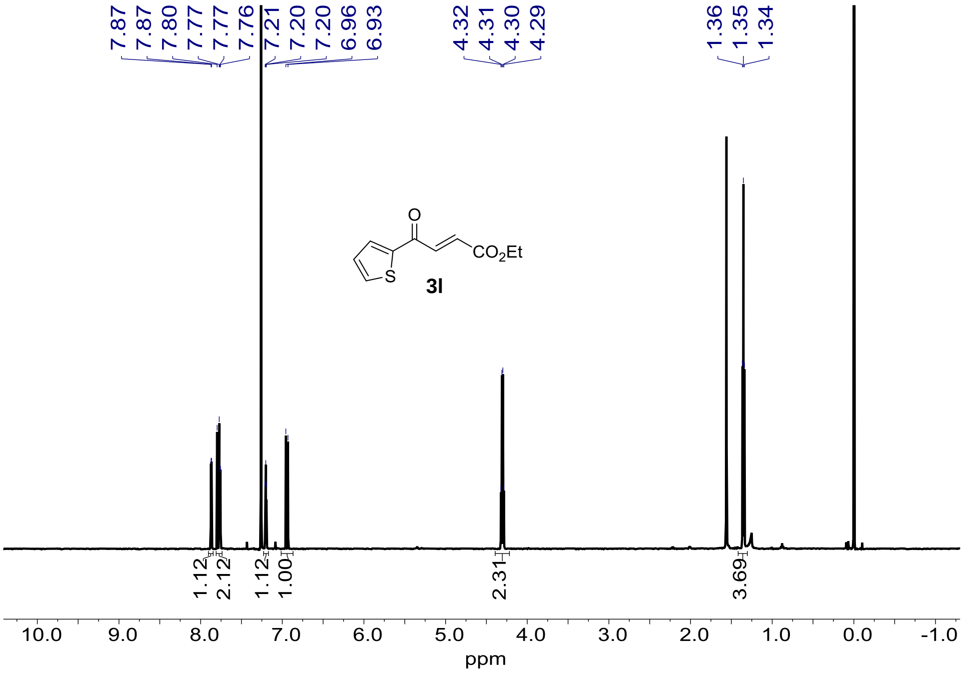

Supplementary Fig. 58. ^{1}H NMR (600 MHz) spectrum of **3l** measured in $CDCl_3$ at 25 °C.

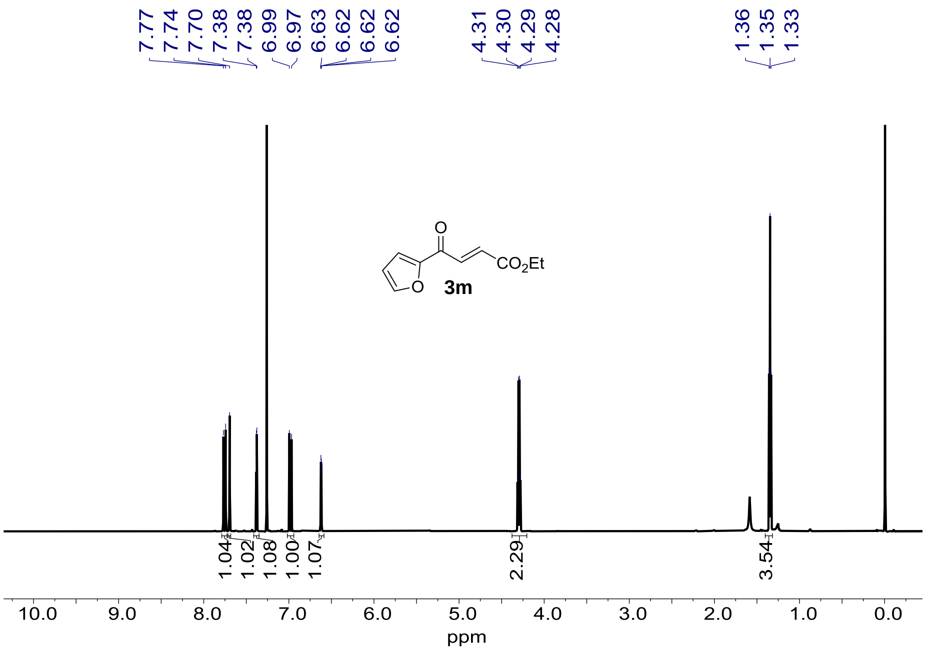

Supplementary Fig. 59. ^{1}H NMR (600 MHz) spectrum of **3m** measured in $CDCl_3$ at 25 °C.

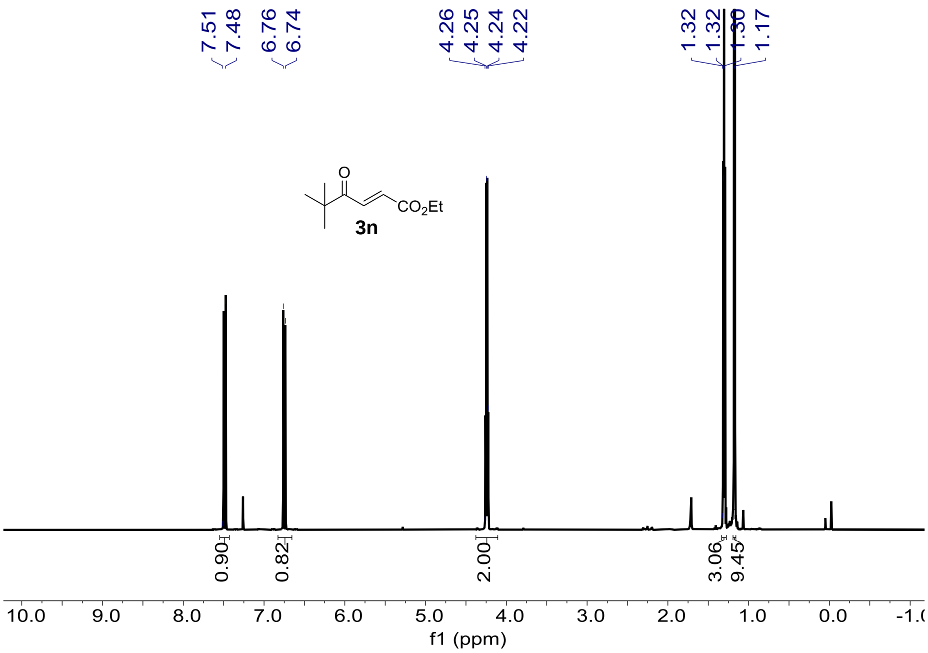

Supplementary Fig. 60. ^{1}H NMR (600 MHz) spectrum of **3n** measured in $CDCl_3$ at 25 °C.

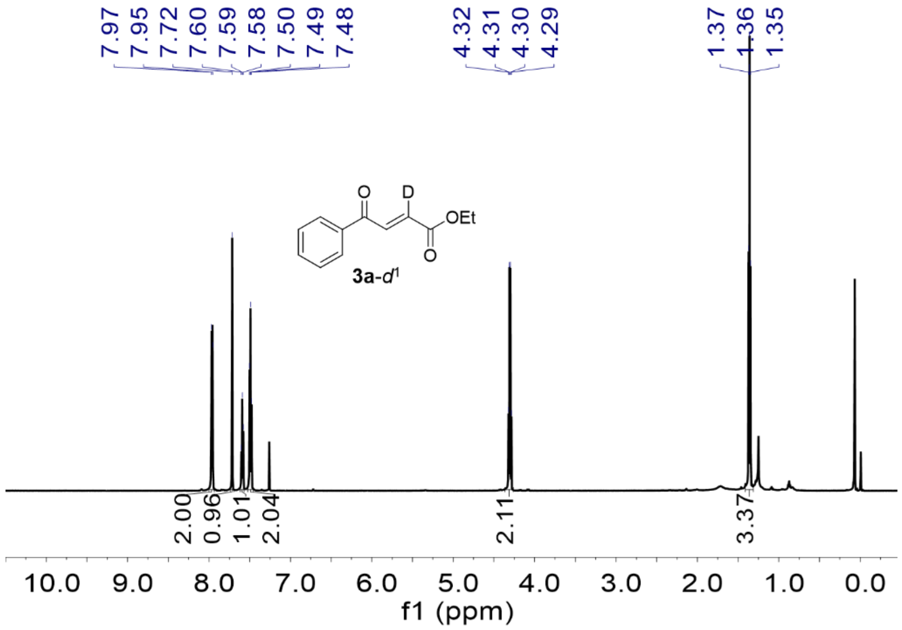

Supplementary Fig. 61. ^{1}H NMR (600 MHz) spectrum of **3a-*d*^{1}** measured in $CDCl_3$ at 25 °C.

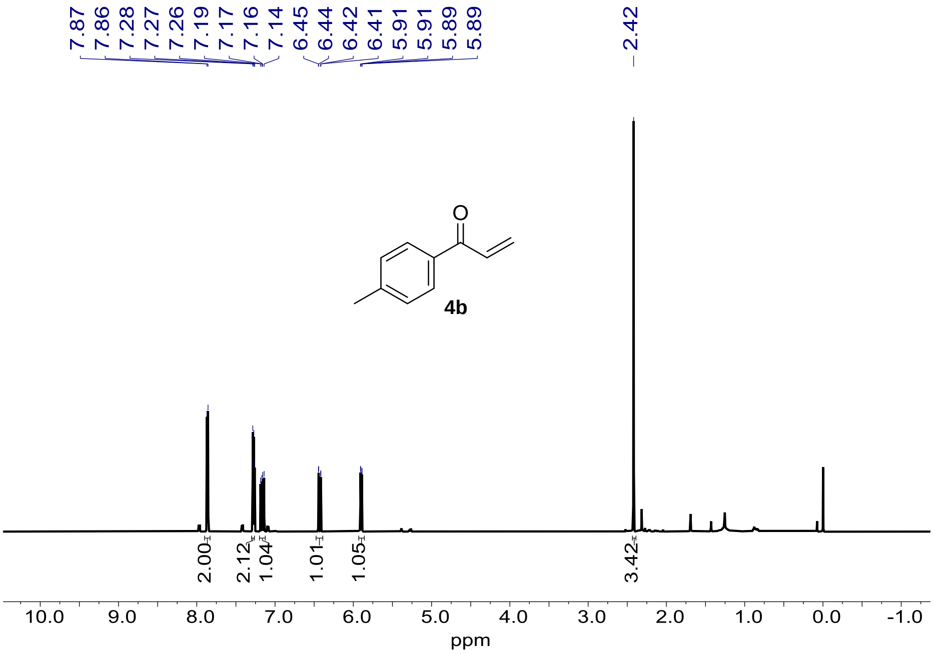

Supplementary Fig. 62. ^{1}H NMR (600 MHz) spectrum of **4b** measured in $CDCl_3$ at 25 °C.

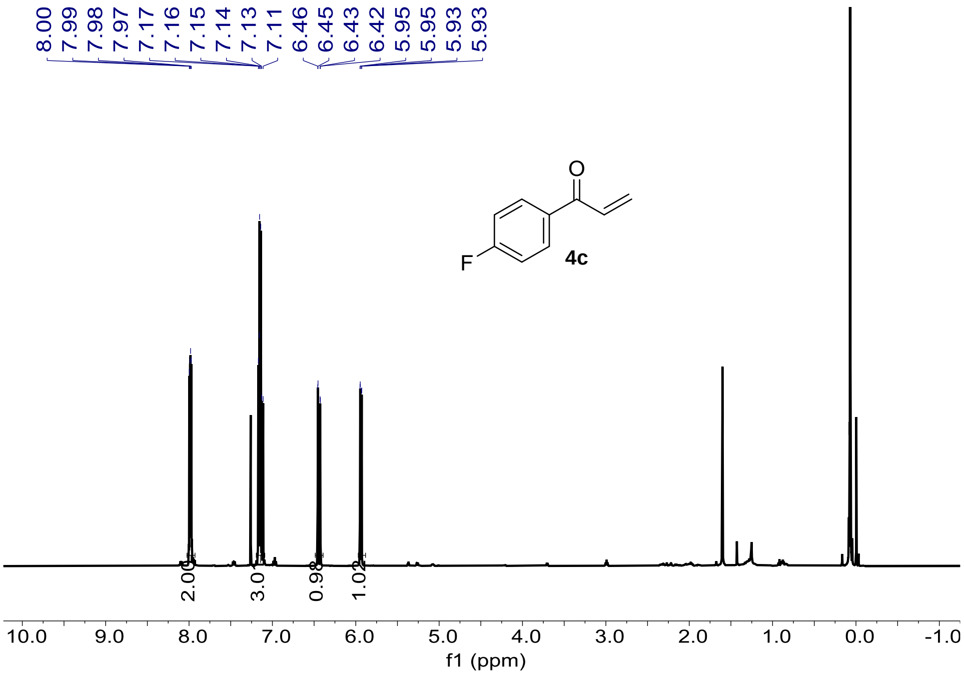

Supplementary Fig. 63. ^{1}H NMR (600 MHz) spectrum of **4c** measured in $CDCl_3$ at 25 °C.

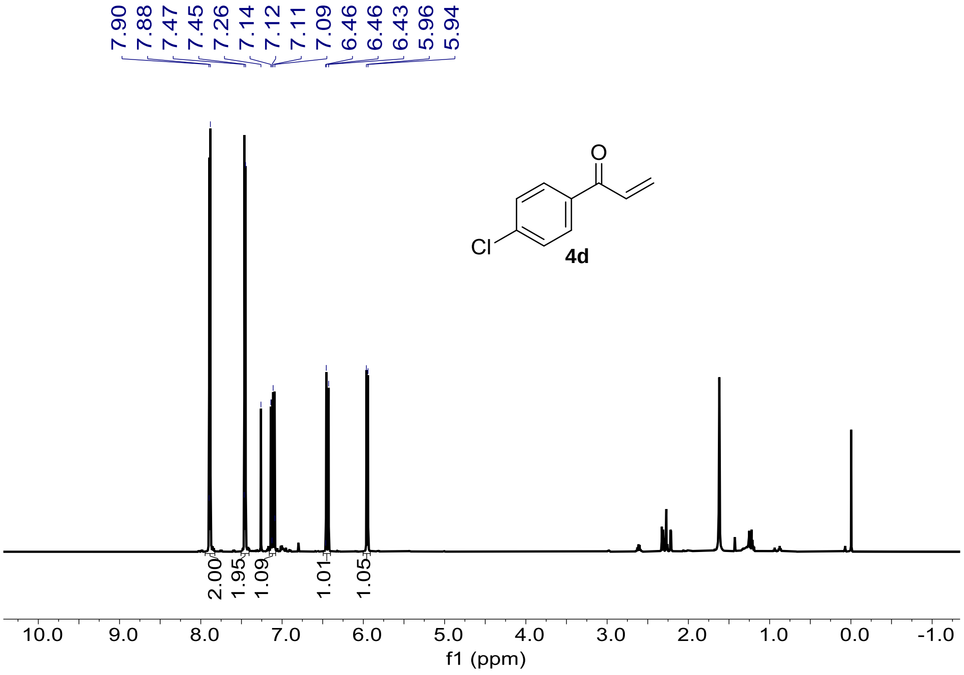

Supplementary Fig. 64. ^{1}H NMR (600 MHz) spectrum of **4d** measured in $CDCl_3$ at 25 °C.

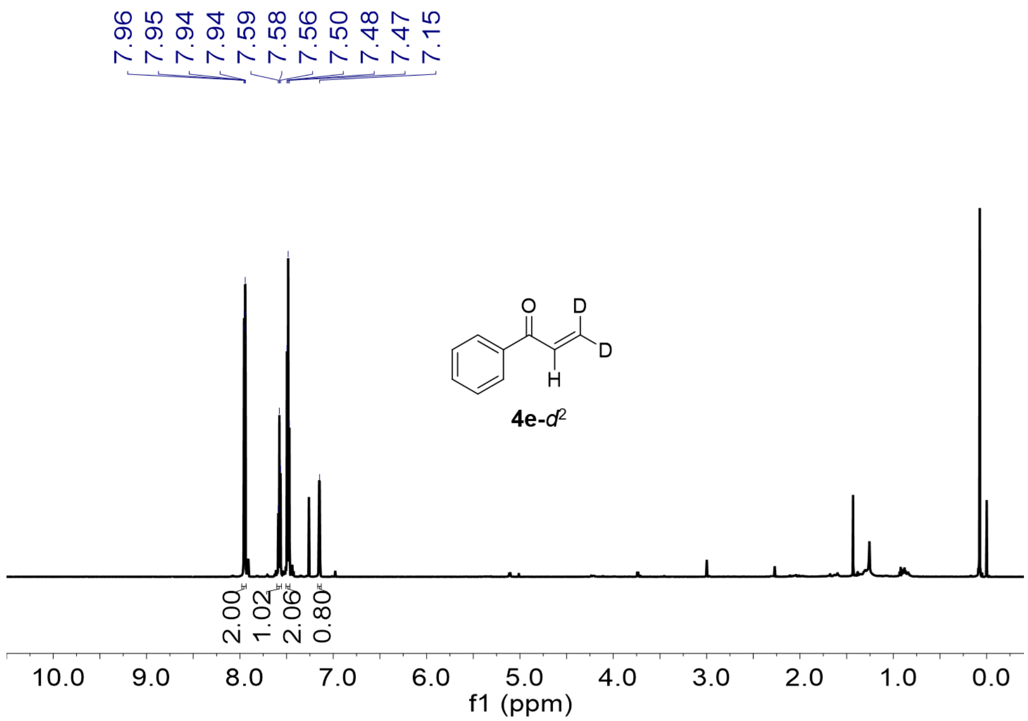

Supplementary Fig. 65. ^{1}H NMR (600 MHz) spectrum of **4e**-d^2 measured in $CDCl_3$ at 25 °C.

HPLC curves of the products.

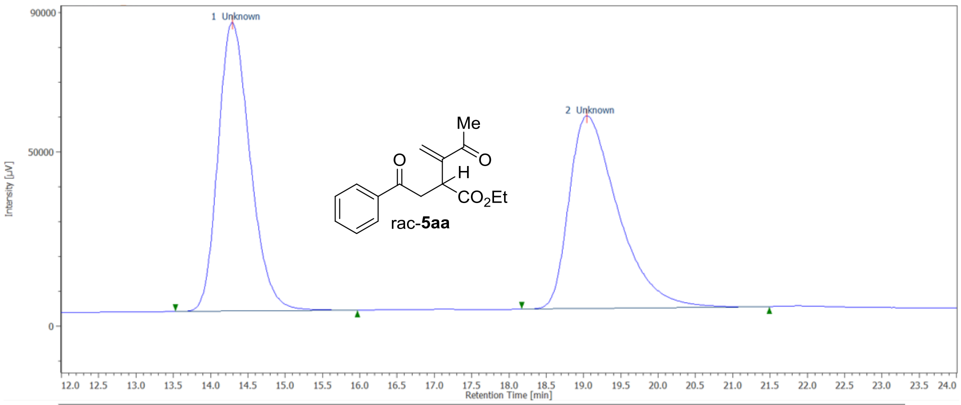

#	Peak Name	tR	Area	Height	Area%	Height%	Peak Start	Peak End	Base Start	Base End	Peak Mark
1	Unknown	14.292	2468604	82609	49.507	59.956	13.525	15.967	13.525	15.967	Manual
2	Unknown	19.042	2517749	55174	50.493	40.044	18.167	21.483	18.167	21.483	Manual

Supplementary Fig. 66. HPLC curve of racemic **5aa** (Chiralpak OZ-H; *n*-hexane/*i*-PrOH = 85/15 (v/v); 1.00 mL/min; 220 nm; 25 °C).

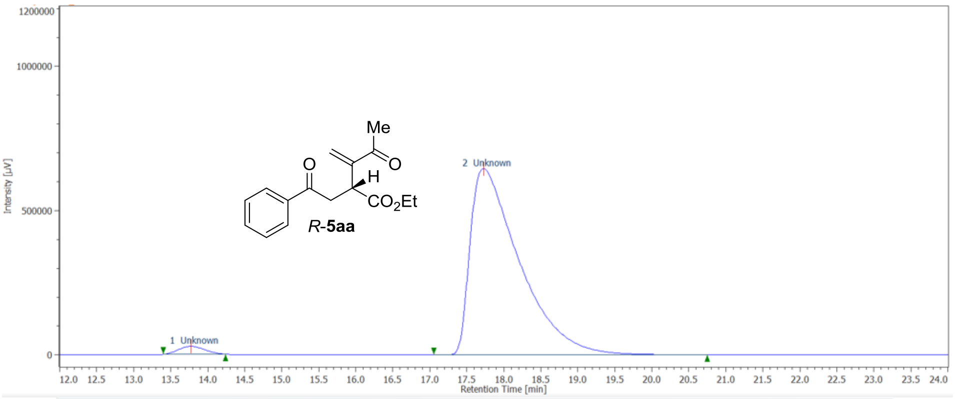

#	Peak Name	tR	Area	Height	Area%	Height%	Peak Start	Peak End	Base Start	Base End	Peak Mark
1	Unknown	13.775	674156	27276	2.211	4.056	13.400	14.242	13.400	14.242	Manual
2	Unknown	17.725	29817237	645189	97.789	95.944	17.050	20.742	17.050	20.742	Manual

Supplementary Fig. 67. HPLC curve of *R*-**5aa** catalyzed by poly(**1s**$_{50}$-*b*-**2**$_{100}$) (Chiralpak OZ-H; *n*-hexane/*i*-PrOH = 85/15 (v/v); 1.00 mL/min; 220 nm; 25 °C).

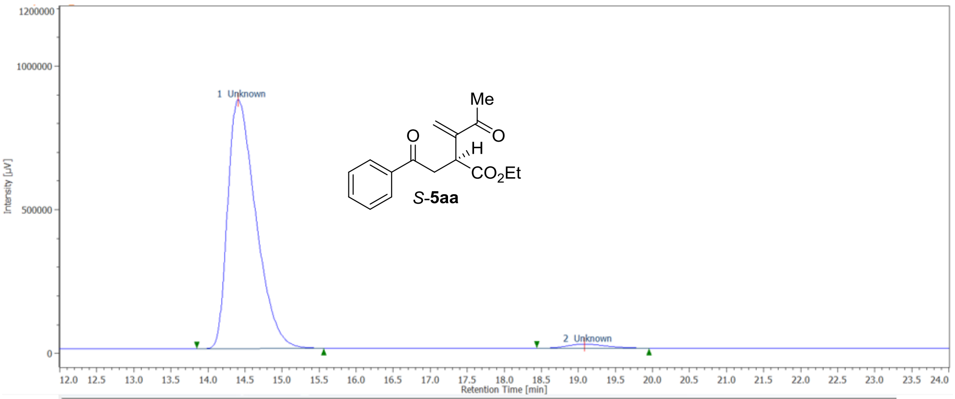

#	Peak Name	tR	Area	Height	Area%	Height%	Peak Start	Peak End	Base Start	Base End	Peak Mark
1	Unknown	14.408	22857341	865634	97.762	98.378	13.850	15.558	13.850	15.558	Manual
2	Unknown	19.075	523154	14269	2.238	1.622	18.433	19.950	18.433	19.950	Manual

Supplementary Fig. 68. HPLC curve of *S*-**5aa** catalyzed by poly(**1r**$_{50}$-*b*-**2**$_{100}$) (Chiralpak OZ-H; *n*-hexane/*i*-PrOH = 85/15 (v/v); 1.00 mL/min; 254 nm; 25 °C).

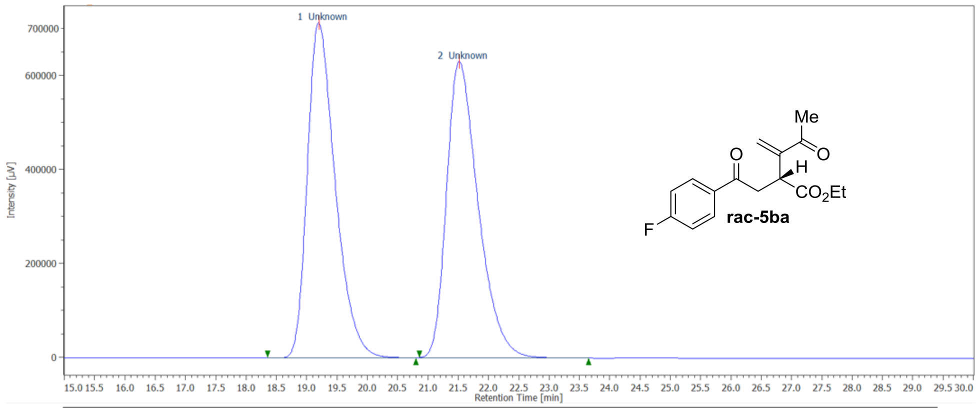

#	Peak Name	tR	Area	Height	Area%	Height%	Peak Start	Peak End	Base Start	Base End	Peak Mark
1	Unknown	19.200	22834738	713105	50.018	53.114	18.350	20.800	18.350	20.800	Manual
2	Unknown	21.517	22818239	629493	49.982	46.886	20.858	23.650	20.858	23.650	Manual

Supplementary Fig. 69. HPLC curve of racemic **5ba** (Chiralpak AD-H; *n*-hexane/*i*-PrOH = 90/10 (v/v); 1.00 mL/min; 220 nm; 25 °C).

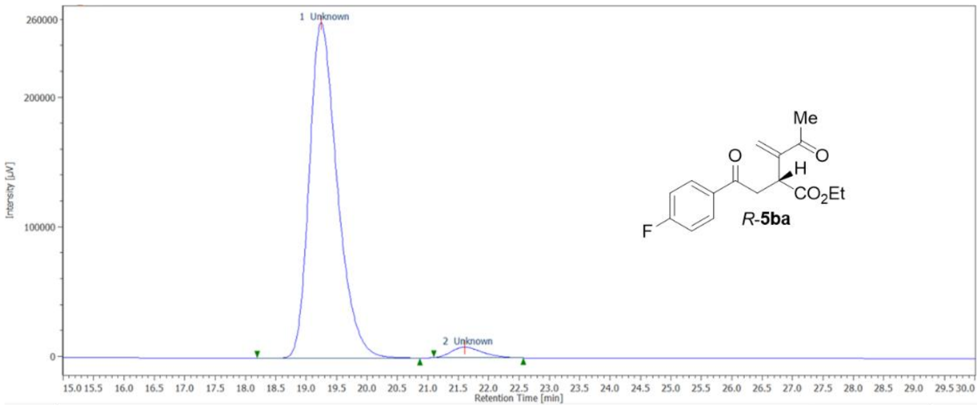

#	Peak Name	tR	Area	Height	Area%	Height%	Peak Start	Peak End	Base Start	Base End	Peak Mark
1	Unknown	19.242	8258045	258431	96.632	96.938	18.192	20.867	18.192	20.867	Manual
2	Unknown	21.600	287805	8164	3.368	3.062	21.100	22.567	21.100	22.567	Manual

Supplementary Fig. 70. HPLC curve of *R*-**5ba** catalyzed by poly(**1s**$_{50}$-*b*-**2**$_{100}$) (Chiralpak AD-H; *n*-hexane/*i*-PrOH = 90/10 (v/v); 1.00 mL/min; 220 nm; 25 °C).

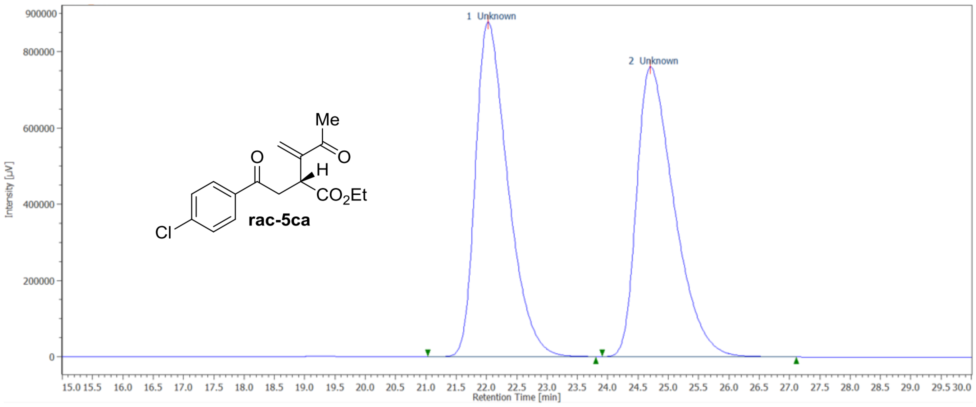

#	Peak Name	tR	Area	Height	Area%	Height%	Peak Start	Peak End	Base Start	Base End	Peak Mark
1	Unknown	22.025	32602516	876709	49.955	53.589	21.025	23.800	21.025	23.800	Manual
2	Unknown	24.700	32661589	759289	50.045	46.411	23.908	27.108	23.908	27.108	Manual

Supplementary Fig. 71. HPLC curve of racemic **5ca** (Chiralpak AD-H; *n*-hexane/*i*-PrOH = 90/10 (v/v); 1.00 mL/min; 220 nm; 25 °C).

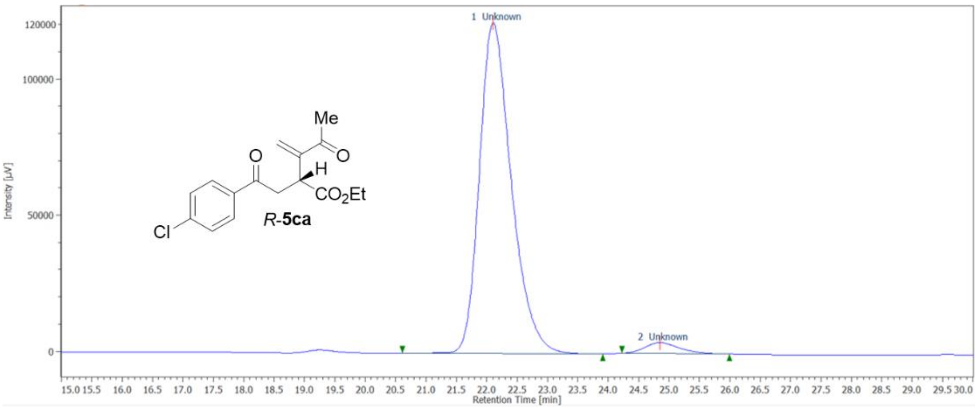

#	Peak Name	tR	Area	Height	Area%	Height%	Peak Start	Peak End	Base Start	Base End	Peak Mark
1	Unknown	22.100	4427712	121346	96.579	96.823	20.608	23.900	20.608	23.900	Manual
2	Unknown	24.842	156822	3982	3.421	3.177	24.217	25.983	24.217	25.983	Manual

Supplementary Fig. 72. HPLC curve of *R*-**5ca** catalyzed by poly(**1s**$_{50}$-*b*-**2**$_{100}$) (Chiralpak AD-H; *n*-hexane/*i*-PrOH = 90/10 (v/v); 1.00 mL/min; 220 nm; 25 °C).

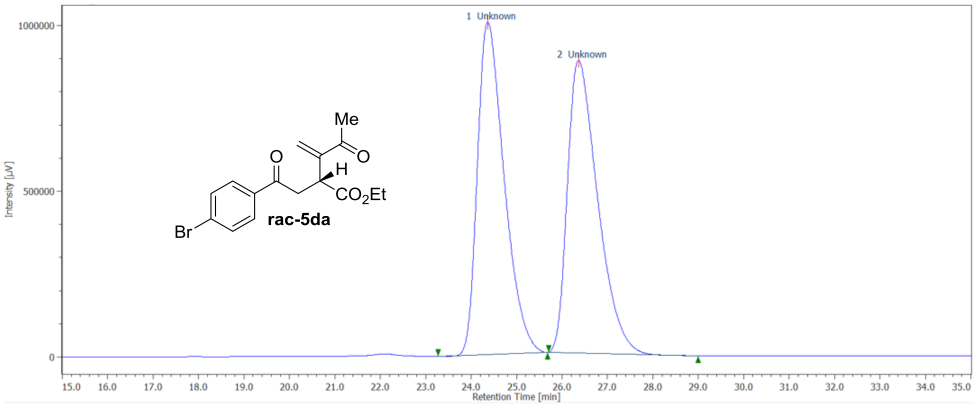

#	Peak Name	tR	Area	Height	Area%	Height%	Peak Start	Peak End	Base Start	Base End	Peak Mark
1	Unknown	24.358	40995179	1001176	50.168	53.193	23.267	25.675	23.267	25.675	Manual
2	Unknown	26.358	40720581	880988	49.832	46.807	25.700	28.983	25.700	28.983	Manual

Supplementary Fig. 73. HPLC curve of racemic **5da** (Chiralpak AD-H; *n*-hexane/*i*-PrOH = 90/10 (v/v); 1.00 mL/min; 220 nm; 25 °C).

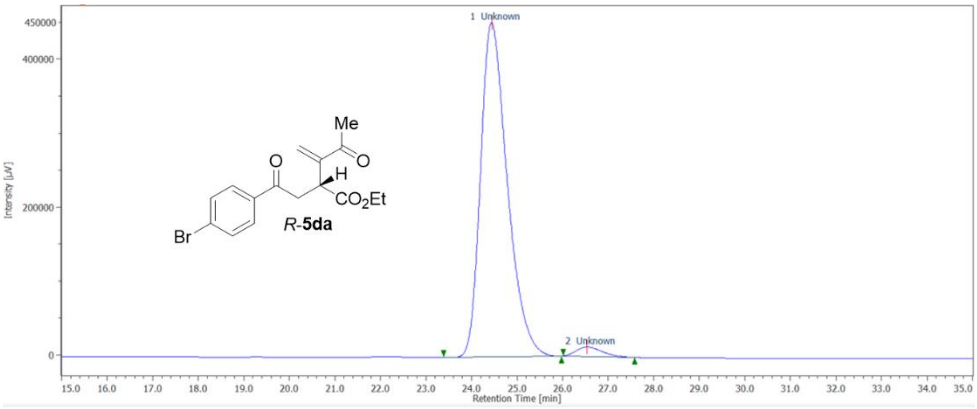

#	Peak Name	tR	Area	Height	Area%	Height%	Peak Start	Peak End	Base Start	Base End	Peak Mark
1	Unknown	24.433	18408582	451833	97.330	97.249	23.383	25.967	23.383	25.967	Manual
2	Unknown	26.533	504985	12781	2.670	2.751	26.008	27.575	26.008	27.575	Manual

Supplementary Fig. 74. HPLC curve of *R*-**5da** catalyzed by poly(**1s**$_{50}$-*b*-**2**$_{100}$) (Chiralpak AD-H; *n*-hexane/*i*-PrOH = 90/10 (v/v); 1.00 mL/min; 220 nm; 25 °C).

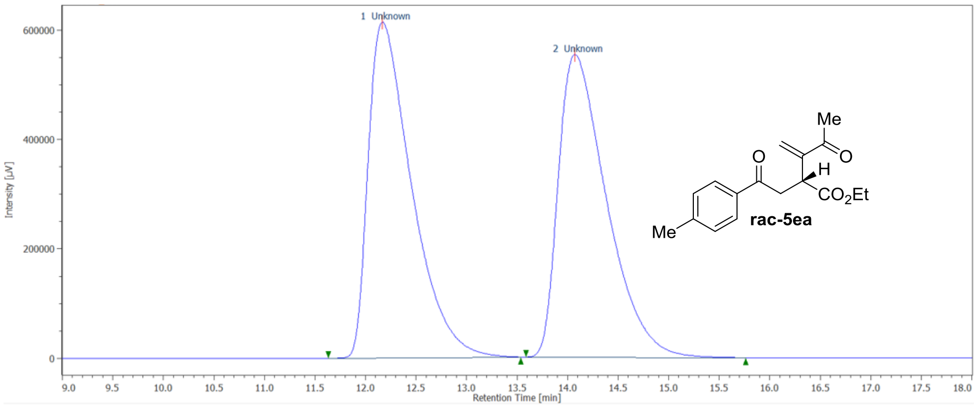

#	Peak Name	tR	Area	Height	Area%	Height%	Peak Start	Peak End	Base Start	Base End	Peak Mark
1	Unknown	12.167	18279468	612551	49.929	52.591	11.633	13.533	11.633	13.533	Manual
2	Unknown	14.067	18331335	552192	50.071	47.409	13.583	15.758	13.583	15.758	Manual

Supplementary Fig. 75. HPLC curve of racemic **5ea** (Chiralpak OD-H; *n*-hexane/*i*-PrOH = 90/10 (v/v); 1.00 mL/min; 220 nm; 25 °C).

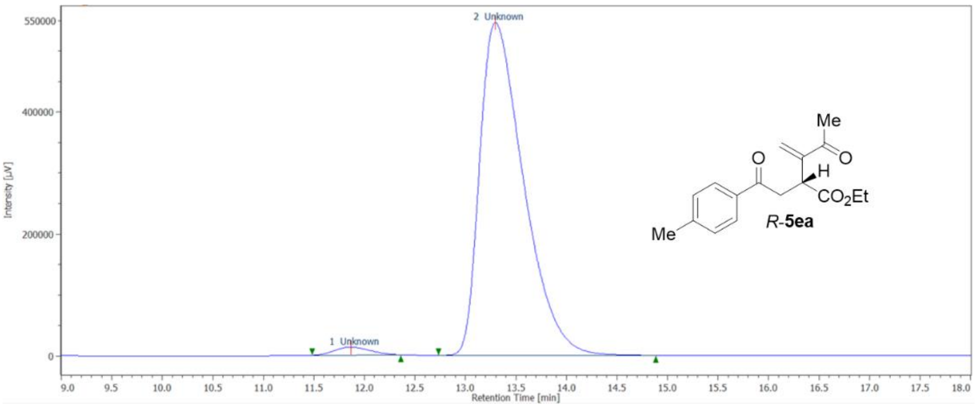

#	Peak Name	tR	Area	Height	Area%	Height%	Peak Start	Peak End	Base Start	Base End	Peak Mark
1	Unknown	11.867	321598	13162	1.919	2.357	11.483	12.358	11.483	12.358	Manual
2	Unknown	13.292	16438944	545297	98.081	97.643	12.733	14.883	12.733	14.883	Manual

Supplementary Fig. 76. HPLC curve of *R*-**5ea** catalyzed by poly(**1s**$_{50}$-*b*-**2**$_{100}$) (Chiralpak AD-H; *n*-hexane/*i*-PrOH = 90/10 (v/v); 1.00 mL/min; 220 nm; 25 °C).

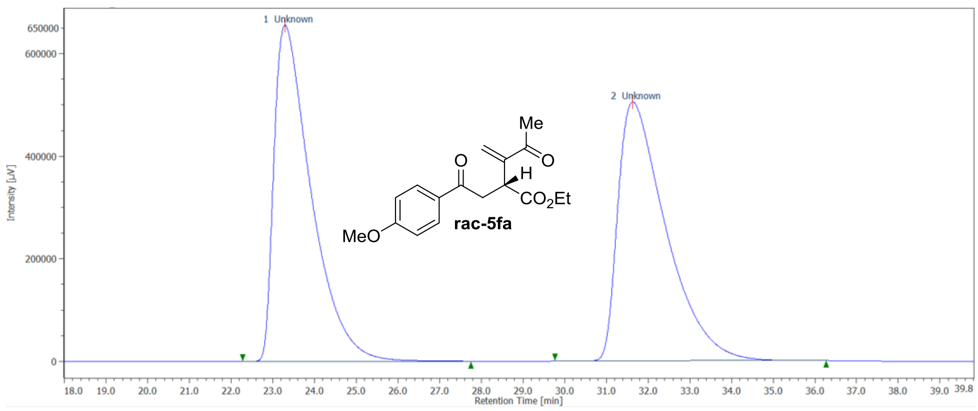

#	Peak Name	tR	Area	Height	Area%	Height%	Peak Start	Peak End	Base Start	Base End	Peak Mark
1	Unknown	23.292	40483916	654916	50.006	56.490	22.275	27.750	22.275	27.750	Manual
2	Unknown	31.625	40474544	504423	49.994	43.510	29.758	36.258	29.758	36.258	Manual

Supplementary Fig. 77. HPLC curve of racemic **5fa** (Chiralpak OD-H; *n*-hexane/*i*-PrOH = 90/10 (v/v); 1.00 mL/min; 220 nm; 25 °C).

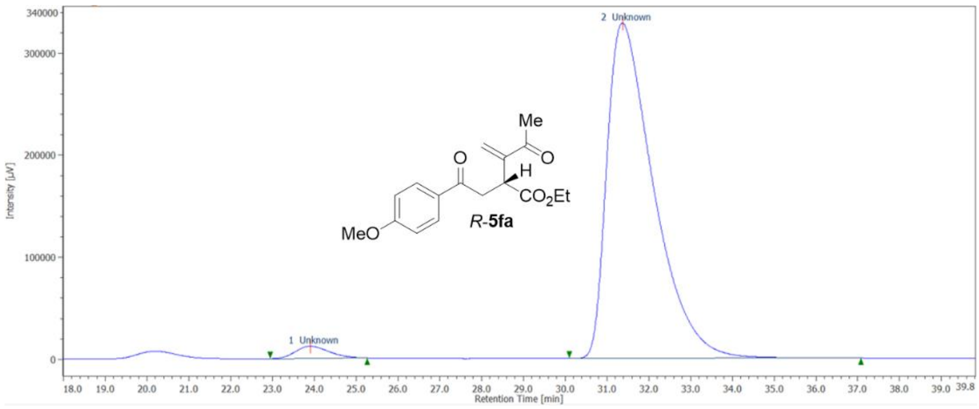

#	Peak Name	tR	Area	Height	Area%	Height%	Peak Start	Peak End	Base Start	Base End	Peak Mark
1	Unknown	23.900	666350	11917	2.553	3.506	22.942	25.258	22.942	25.258	Manual
2	Unknown	31.367	25437878	327968	97.447	96.494	30.092	37.067	30.092	37.067	Manual

Supplementary Fig. 78. HPLC curve of *R*-**5fa** catalyzed by poly(**1s**$_{50}$-*b*-**2**$_{100}$) (Chiralpak OD-H; *n*-hexane/*i*-PrOH = 90/10 (v/v); 1.00 mL/min; 220 nm; 25 °C).

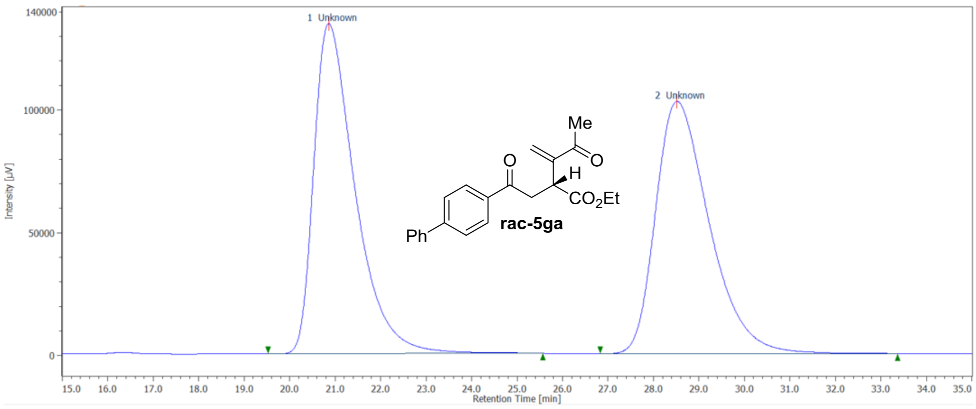

#	Peak Name	tR	Area	Height	Area%	Height%	Peak Start	Peak End	Base Start	Base End	Peak Mark
1	Unknown	20.858	8463146	134233	50.182	56.690	19.525	25.558	19.525	25.558	Manual
2	Unknown	28.508	8401862	102552	49.818	43.310	26.825	33.358	26.825	33.358	Manual

Supplementary Fig. 79. HPLC curve of racemic **5ga** (Chiralpak OZ-H; *n*-hexane/*i*-PrOH = 80/20 (v/v); 1.00 mL/min; 220 nm; 25 °C).

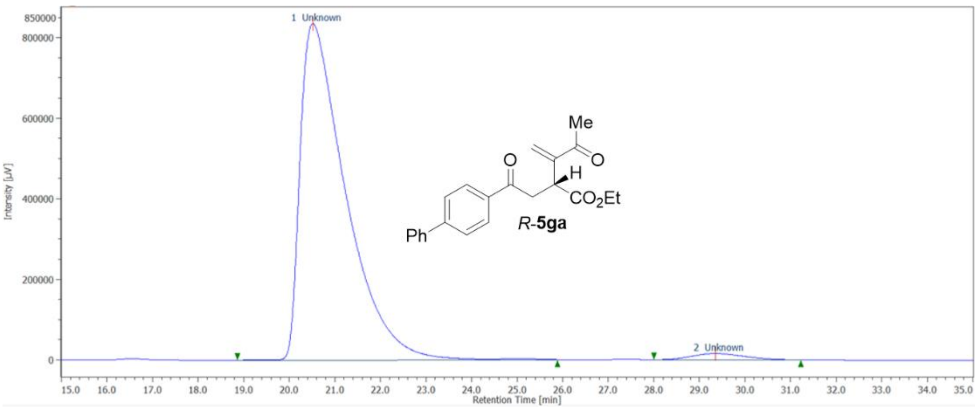

#	Peak Name	tR	Area	Height	Area%	Height%	Peak Start	Peak End	Base Start	Base End	Peak Mark
1	Unknown	20.517	57716346	834129	97.930	98.196	18.858	25.883	18.858	25.883	Manual
2	Unknown	29.342	1219895	15327	2.070	1.804	27.992	31.217	27.992	31.217	Manual

Supplementary Fig. 80. HPLC curve of *R*-**5ga** catalyzed by poly($\mathbf{1s}_{50}$-*b*-$\mathbf{2}_{100}$) (Chiralpak OZ-H; *n*-hexane/*i*-PrOH = 80/20 (v/v); 1.00 mL/min; 220 nm; 25 °C).

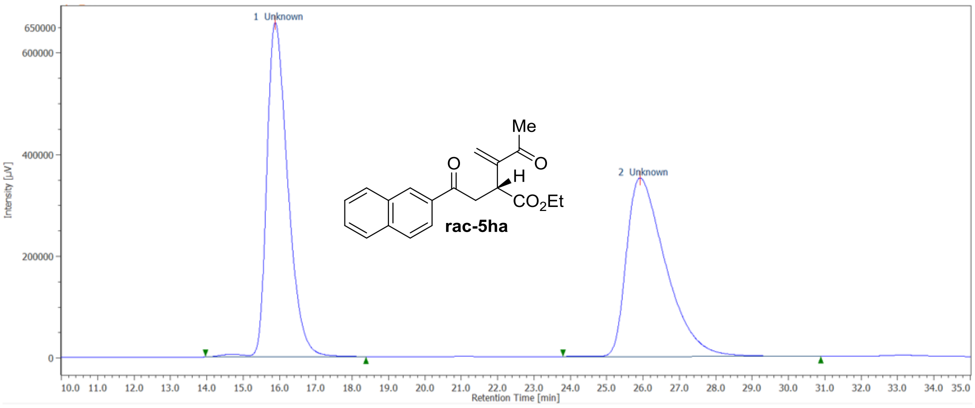

#	Peak Name	tR	Area	Height	Area%	Height%	Peak Start	Peak End	Base Start	Base End	Peak Mark
1	Unknown	15.883	26344162	656544	50.054	65.188	13.967	18.375	13.967	18.375	Manual
2	Unknown	25.908	26287008	350605	49.946	34.812	23.792	30.875	23.792	30.875	Manual

Supplementary Fig. 81. HPLC curve of racemic **5ha** (Chiralpak OZ-H; *n*-hexane/*i*-PrOH = 80/20 (v/v); 1.00 mL/min; 220 nm; 25 °C).

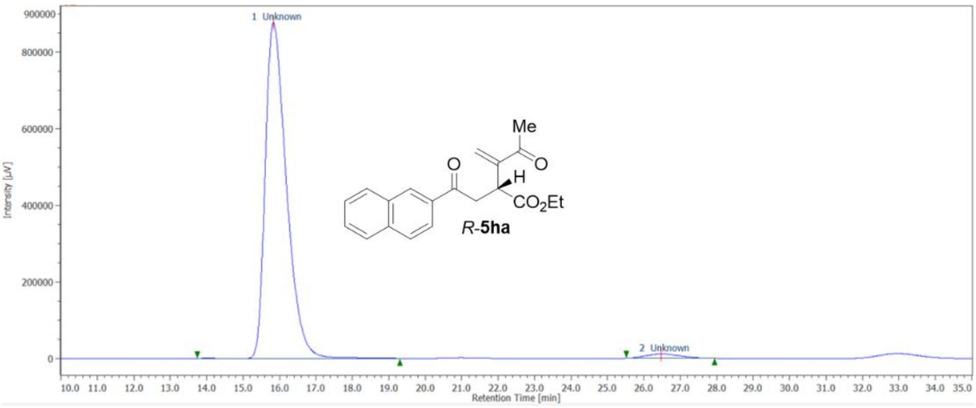

#	Peak Name	tR	Area	Height	Area%	Height%	Peak Start	Peak End	Base Start	Base End	Peak Mark
1	Unknown	15.833	35258741	875597	97.953	98.748	13.742	19.300	13.742	19.300	Manual
2	Unknown	26.467	736737	11105	2.047	1.252	25.508	27.933	25.508	27.933	Manual

Supplementary Fig. 82. HPLC curve of *R*-**5ha** catalyzed by poly(**1s**$_{50}$-*b*-**2**$_{100}$) (Chiralpak OZ-H; *n*-hexane/*i*-PrOH = 80/20 (v/v); 1.00 mL/min; 220 nm; 25 °C).

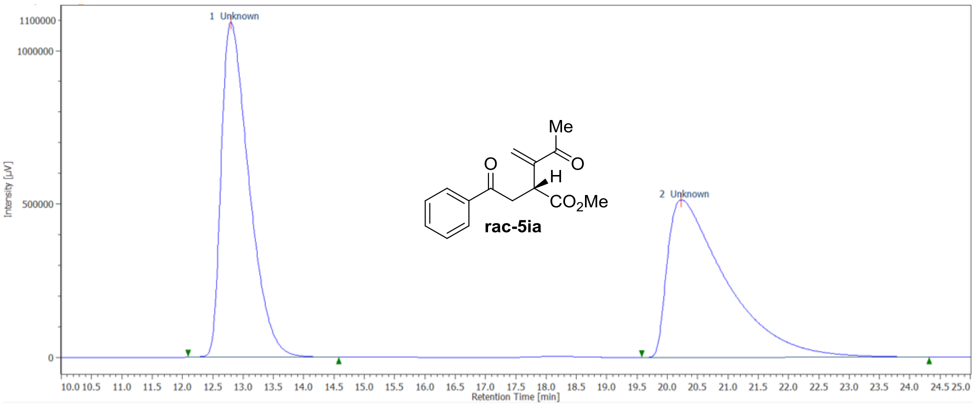

#	Peak Name	tR	Area	Height	Area%	Height%	Peak Start	Peak End	Base Start	Base End	Peak Mark
1	Unknown	12.800	34661055	1091080	49.999	68.023	12.092	14.575	12.092	14.575	Manual
2	Unknown	20.225	34663022	512895	50.001	31.977	19.575	24.308	19.575	24.308	Manual

Supplementary Fig. 83. HPLC curve of racemic **5ia** (Chiralpak OZ-H; *n*-hexane/*i*-PrOH = 70/30 (v/v); 1.00 mL/min; 220 nm; 25 °C).

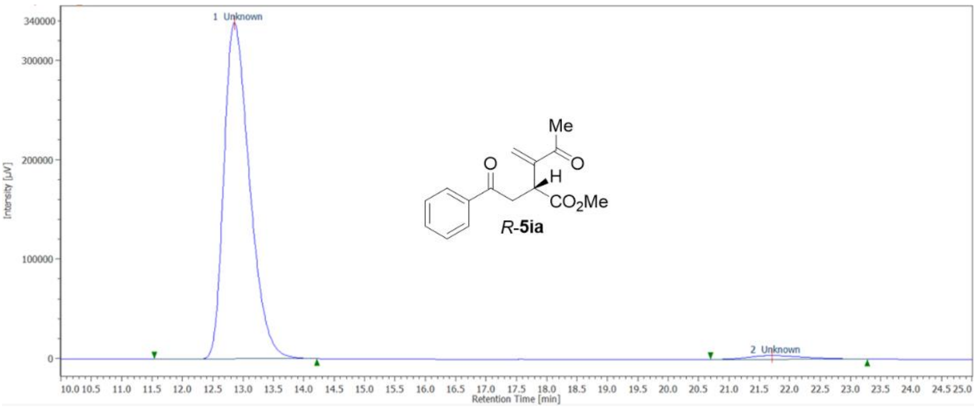

#	Peak Name	tR	Area	Height	Area%	Height%	Peak Start	Peak End	Base Start	Base End	Peak Mark
1	Unknown	12.858	9897402	337895	97.632	98.912	11.542	14.217	11.542	14.217	Manual
2	Unknown	21.708	240060	3715	2.368	1.088	20.692	23.275	20.692	23.275	Manual

Supplementary Fig. 84. HPLC curve of *R*-**5ia** catalyzed by poly($\mathbf{1s}_{50}$-*b*-$\mathbf{2}_{100}$) (Chiralpak OZ-H; *n*-hexane/*i*-PrOH = 70/30 (v/v); 1.00 mL/min; 220 nm; 25 °C).

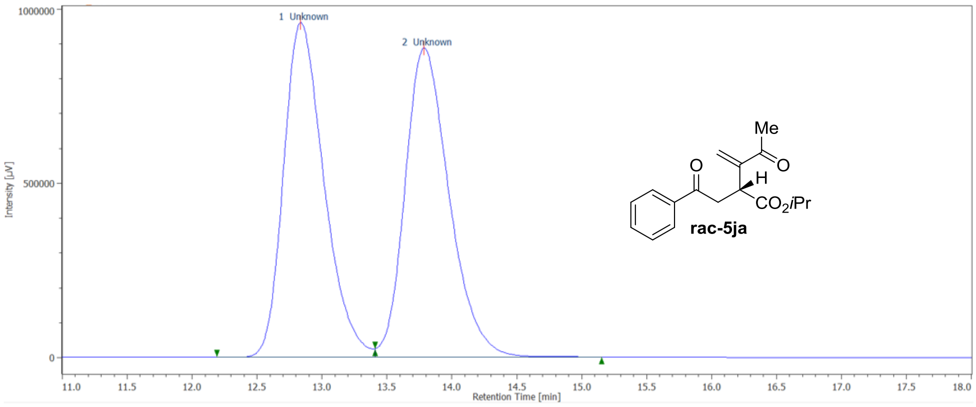

#	Peak Name	tR	Area	Height	Area%	Height%	Peak Start	Peak End	Base Start	Base End	Peak Mark
1	Unknown	12.833	20732387	958506	49.810	51.975	12.192	13.408	12.192	15.150	Manual
2	Unknown	13.783	20890292	885678	50.190	48.025	13.408	15.150	12.192	15.150	Manual

Supplementary Fig. 85. HPLC curve of racemic **5ja** (Chiralpak AD-H; *n*-hexane/*i*-PrOH = 90/10 (v/v); 1.00 mL/min; 220 nm; 25 °C).

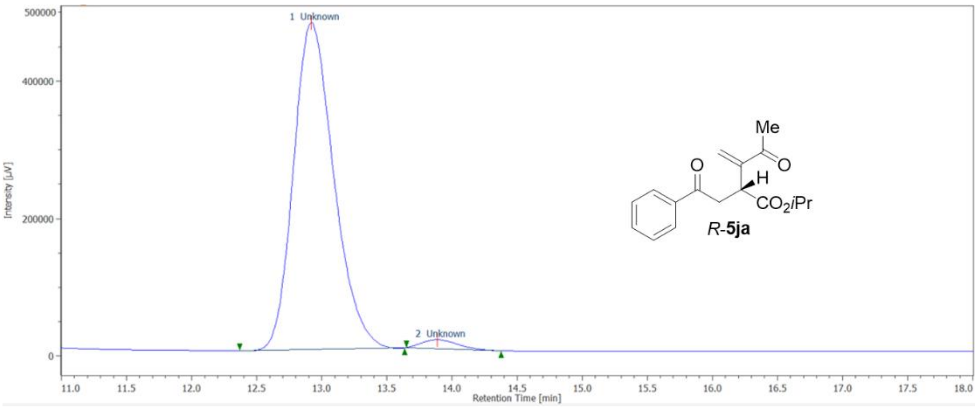

#	Peak Name	tR	Area	Height	Area%	Height%	Peak Start	Peak End	Base Start	Base End	Peak Mark
1	Unknown	12.917	10214292	474740	97.672	97.389	12.367	13.633	12.367	13.633	Manual
2	Unknown	13.883	243410	12725	2.328	2.611	13.650	14.375	13.650	14.375	Manual

Supplementary Fig. 86. HPLC curve of *R*-**5ja** catalyzed by poly(**1s**$_{50}$-*b*-**2**$_{100}$) (Chiralpak AD-H; *n*-hexane/*i*-PrOH = 90/10 (v/v); 1.00 mL/min; 220 nm; 25 °C).

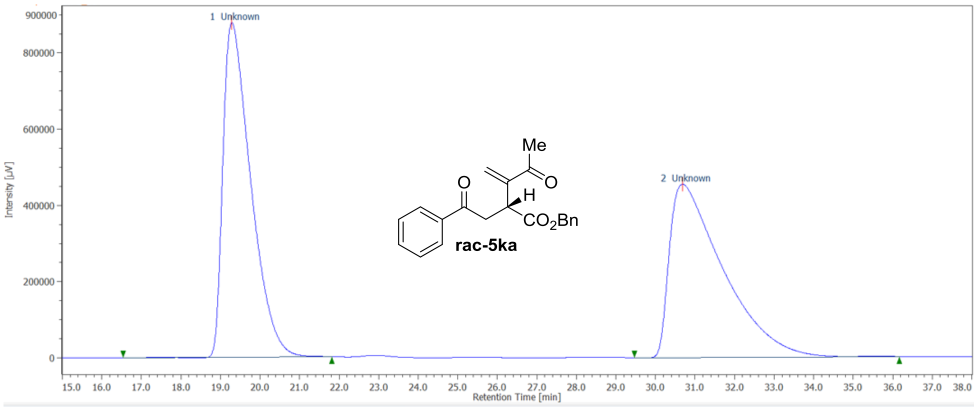

#	Peak Name	tR	Area	Height	Area%	Height%	Peak Start	Peak End	Base Start	Base End	Peak Mark
1	Unknown	19.283	42793440	876796	50.052	65.891	16.542	21.808	16.542	21.808	Manual
2	Unknown	30.675	42704708	453879	49.948	34.109	29.458	36.150	29.458	36.150	Manual

Supplementary Fig. 87. HPLC curve of racemic **5ka** (Chiralpak OZ-H; *n*-hexane/*i*-PrOH = 85/15 (v/v); 1.00 mL/min; 220 nm; 25 °C).

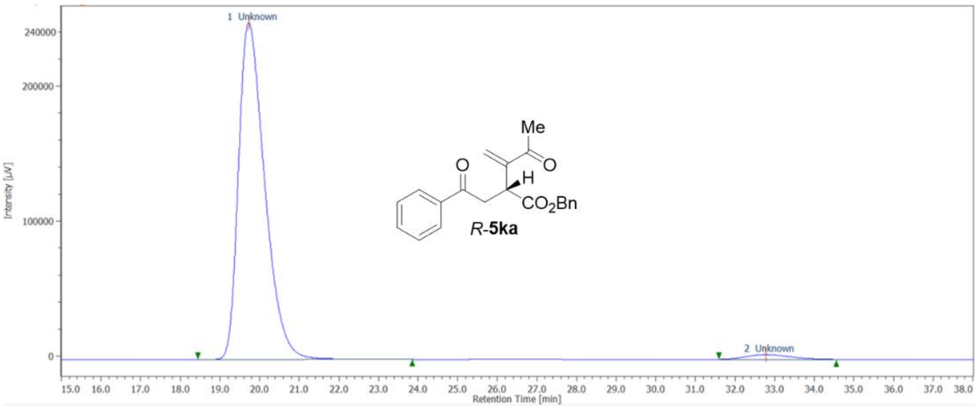

#	Peak Name	tR	Area	Height	Area%	Height%	Peak Start	Peak End	Base Start	Base End	Peak Mark
1	Unknown	19.725	11436363	249119	97.711	98.615	18.442	23.850	18.442	23.850	Manual
2	Unknown	32.767	267884	3498	2.289	1.385	31.583	34.542	31.583	34.542	Manual

Supplementary Fig. 88. HPLC curve of *R*-**5ka** catalyzed by poly(**1s**$_{50}$-*b*-**2**$_{100}$) (Chiralpak OZ-H; *n*-hexane/*i*-PrOH = 85/15 (v/v); 1.00 mL/min; 220 nm; 25 °C).

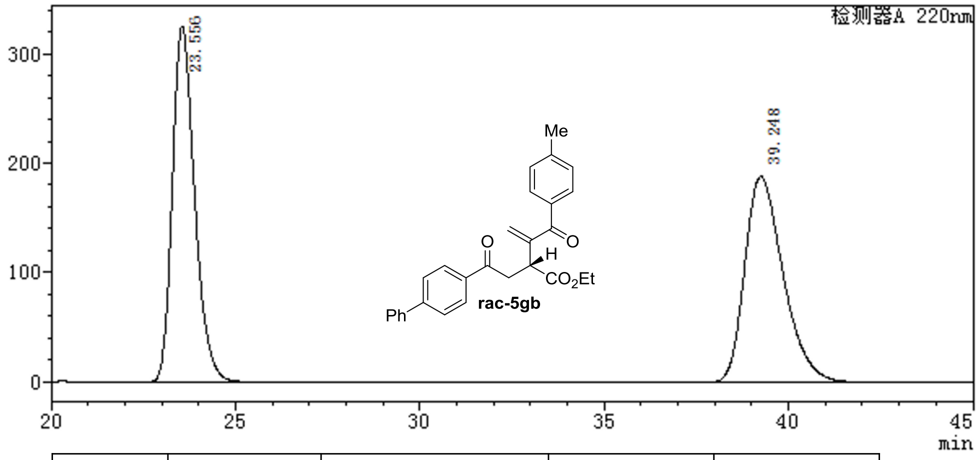

Peak	Ret.Time<br>min	Area<br>mV*min(×10³)	Height<br>mV(×10³)	Rel.Area<br>%
1	23.556	13654.089	325.368	49.970
2	39.248	13670.246	188.458	50.030

Supplementary Fig. 89. HPLC curve of racemic **5gb** (Chiralpak AD-H; *n*-hexane/*i*-PrOH = 70/30 (v/v); 1.00 mL/min; 220 nm; 25 °C).

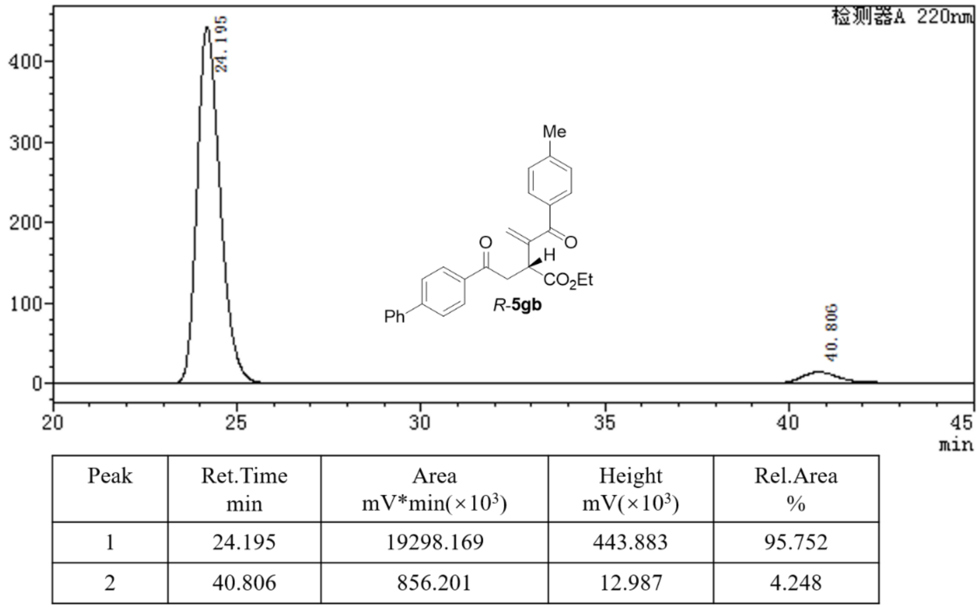

Peak	Ret.Time<br>min	Area<br>mV*min(×10³)	Height<br>mV(×10³)	Rel.Area<br>%
1	24.195	19298.169	443.883	95.752
2	40.806	856.201	12.987	4.248

Supplementary Fig. 90. HPLC curve of *R*-**5gb** catalyzed by poly($\mathbf{1s}_{50}$-*b*-$\mathbf{2}_{100}$) (Chiralpak AD-H; *n*-hexane/*i*-PrOH = 70/30 (v/v); 1.00 mL/min; 220 nm; 25 °C).

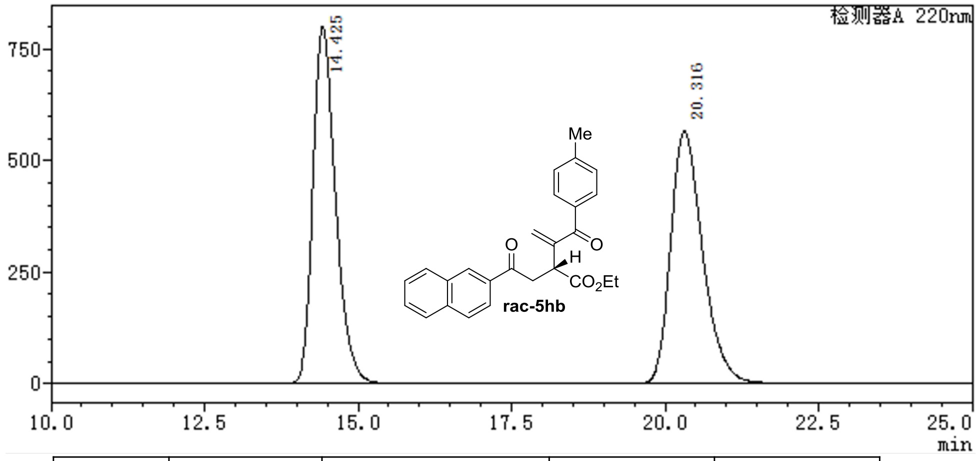

Peak	Ret.Time min	Area mV*min(×10^3)	Height mV(×10^3)	Rel.Area %
1	14.425	20574.447	800.918	49.918
2	20.316	20641.721	565.483	50.082

Supplementary Fig. 91. HPLC curve of racemic **5hb** (Chiralpak AD-H; *n*-hexane/*i*-PrOH = 70/30 (v/v); 1.00 mL/min; 220 nm; 25 °C).

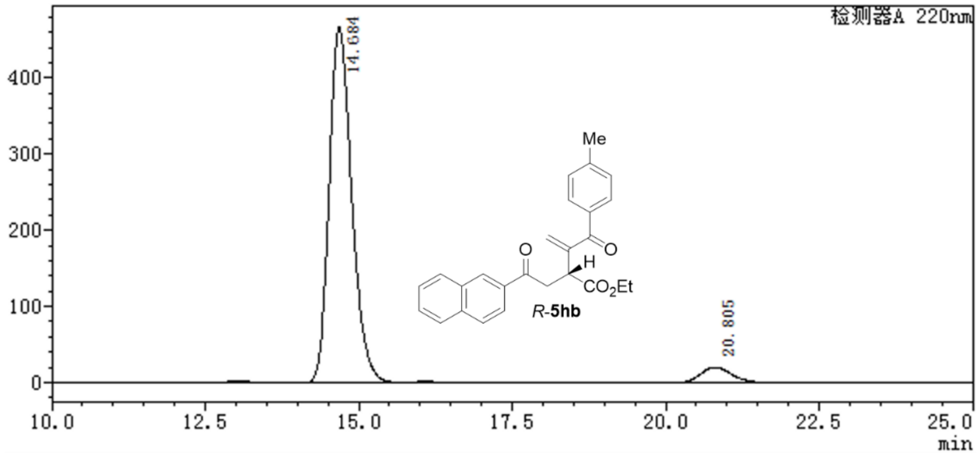

Peak	Ret.Time min	Area mV*min(×10^3)	Height mV(×10^3)	Rel.Area %
1	14.684	12022.738	467.607	95.090
2	20.805	620.858	19.006	4.910

Supplementary Fig. 92. HPLC curve of *R*-**5hb** catalyzed by poly(**1s**$_{50}$-*b*-**2**$_{100}$) (Chiralpak AD-H; *n*-hexane/*i*-PrOH = 70/30 (v/v); 1.00 mL/min; 220 nm; 25 °C).

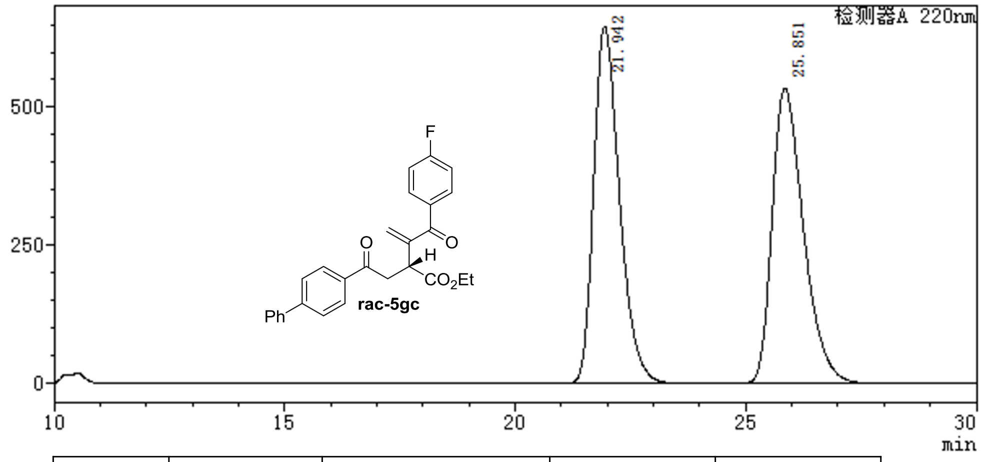

Peak	Ret.Time min	Area mV*min($\times10^3$)	Height mV($\times10^3$)	Rel.Area %
1	21.942	25270.432	646.659	49.952
2	25.851	25319.298	534.089	50.048

Supplementary Fig. 93. HPLC curve of racemic **5gc** (Chiralpak AD-H; *n*-hexane/*i*-PrOH = 70/30 (v/v); 1.00 mL/min; 220 nm; 25 °C).

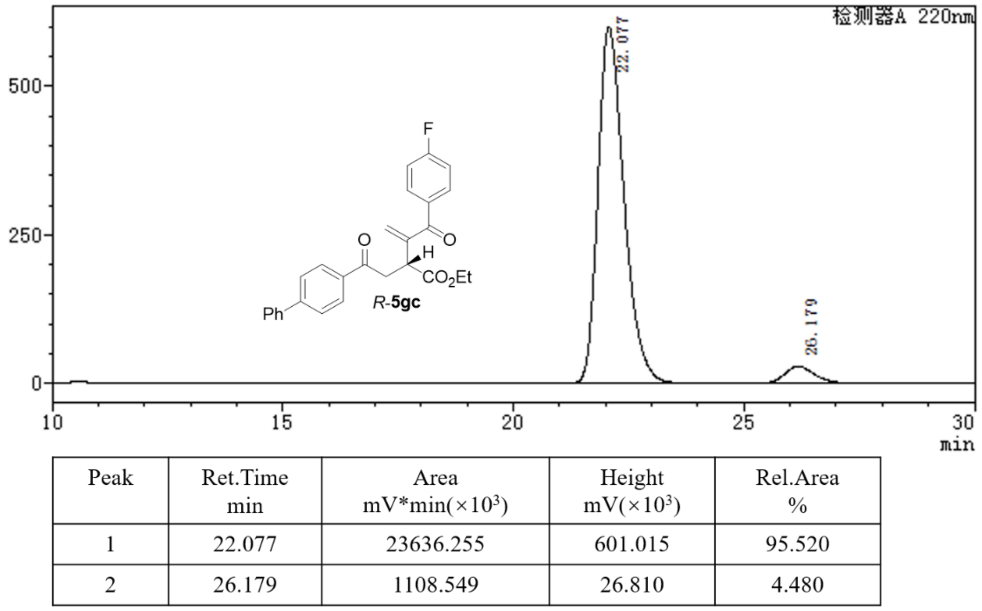

Peak	Ret.Time min	Area mV*min($\times10^3$)	Height mV($\times10^3$)	Rel.Area %
1	22.077	23636.255	601.015	95.520
2	26.179	1108.549	26.810	4.480

Supplementary Fig. 94. HPLC curve of *R*-**5gc** catalyzed by poly(**1s**$_{50}$-*b*-**2**$_{100}$) (Chiralpak AD-H; *n*-hexane/*i*-PrOH = 70/30 (v/v); 1.00 mL/min; 220 nm; 25 °C).

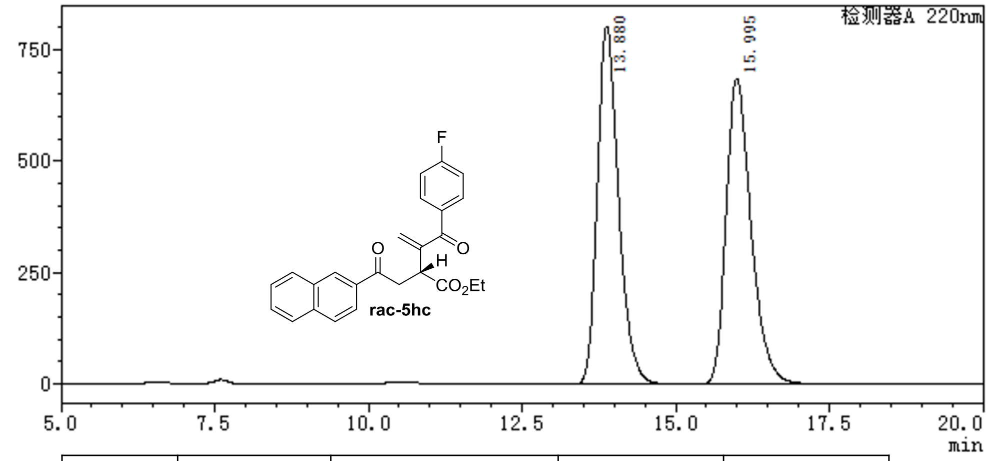

Peak	Ret.Time<br>min	Area<br>mV*min($\times 10^3$)	Height<br>mV($\times 10^3$)	Rel.Area<br>%
1	13.880	19329.168	803.325	49.934
2	15.995	19379.881	686.123	50.066

Supplementary Fig. 95. HPLC curve of racemic **5hc** (Chiralpak AD-H; *n*-hexane/*i*-PrOH = 70/30 (v/v); 1.00 mL/min; 220 nm; 25 °C).

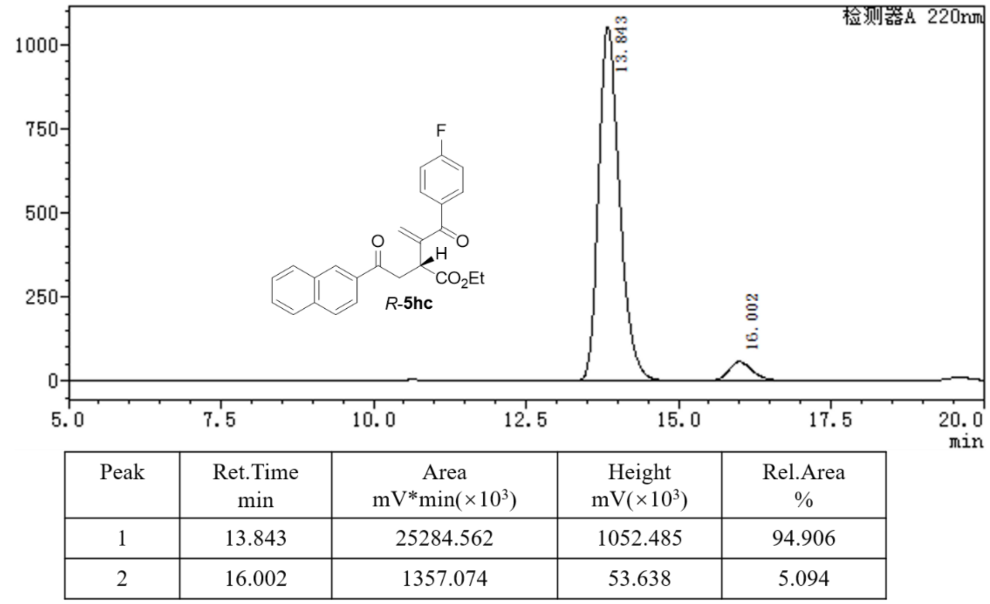

Peak	Ret.Time<br>min	Area<br>mV*min($\times 10^3$)	Height<br>mV($\times 10^3$)	Rel.Area<br>%
1	13.843	25284.562	1052.485	94.906
2	16.002	1357.074	53.638	5.094

Supplementary Fig. 96. HPLC curve of *R*-**5hc** catalyzed by poly(**1s**$_{50}$-*b*-**2**$_{100}$) (Chiralpak AD-H; *n*-hexane/*i*-PrOH = 70/30 (v/v); 1.00 mL/min; 220 nm; 25 °C).

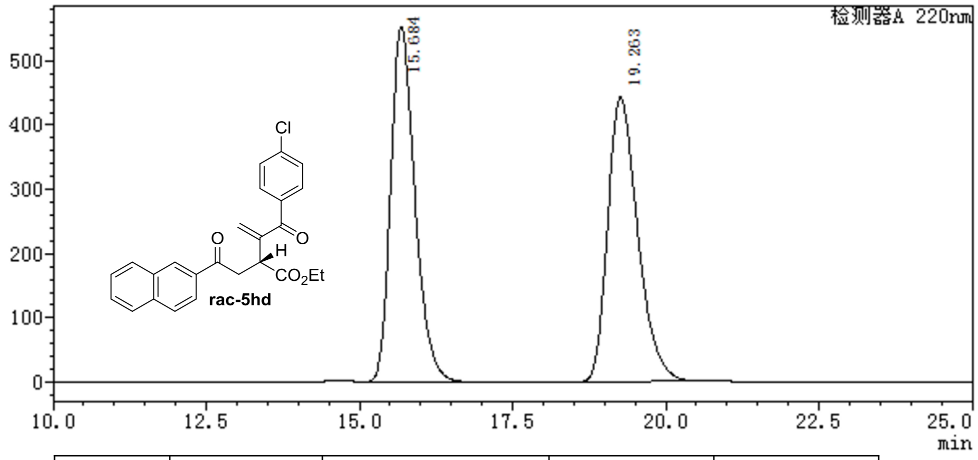

Peak	Ret.Time min	Area mV*min(×10³)	Height mV(×10³)	Rel.Area %
1	15.684	15208.774	552.858	49.951
2	19.263	15238.323	442.855	50.049

Supplementary Fig. 97. HPLC curve of racemic **5hd** (Chiralpak AD-H; *n*-hexane/*i*-PrOH = 70/30 (v/v); 1.00 mL/min; 220 nm; 25 °C).

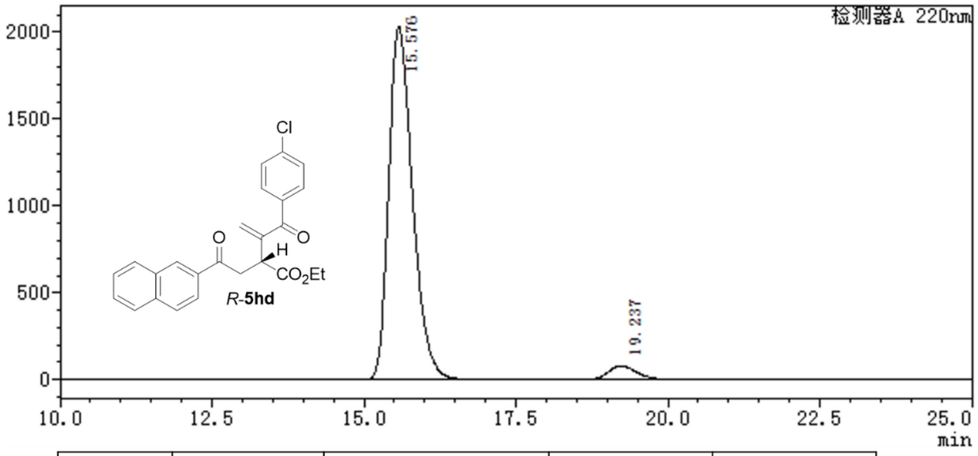

Peak	Ret.Time min	Area mV*min(×10³)	Height mV(×10³)	Rel.Area %
1	15.576	55431.855	2034.465	96.248
2	19.237	2161.050	72.799	3.752

Supplementary Fig. 98. HPLC curve of *R*-**5hd** catalyzed by poly(**1s**$_{50}$-*b*-**2**$_{100}$) (Chiralpak AD-H; *n*-hexane/*i*-PrOH = 70/30 (v/v); 1.00 mL/min; 220 nm; 25 °C).

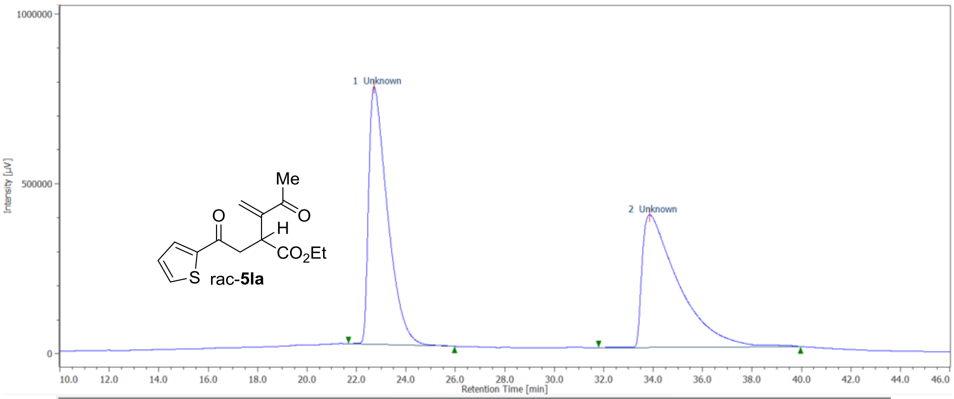

#	Peak Name	tR	Area	Height	Area%	Height%	Peak Start	Peak End	Base Start	Base End	Peak Mark
1	Unknown	22.708	40176528	756769	50.147	65.993	21.675	25.950	21.675	25.950	Manual
2	Unknown	33.850	39941313	389972	49.853	34.007	31.792	39.942	31.792	39.942	Manual

Supplementary Fig. 99. HPLC curve of *rac*-**5la** catalyzed by poly($\mathbf{1s}_{50}$-*b*-$\mathbf{2}_{100}$) (Chiralpak OZ-H; *n*-hexane/*i*-PrOH = 85/15 (v/v); 1.00 mL/min; 220 nm; 25 °C).

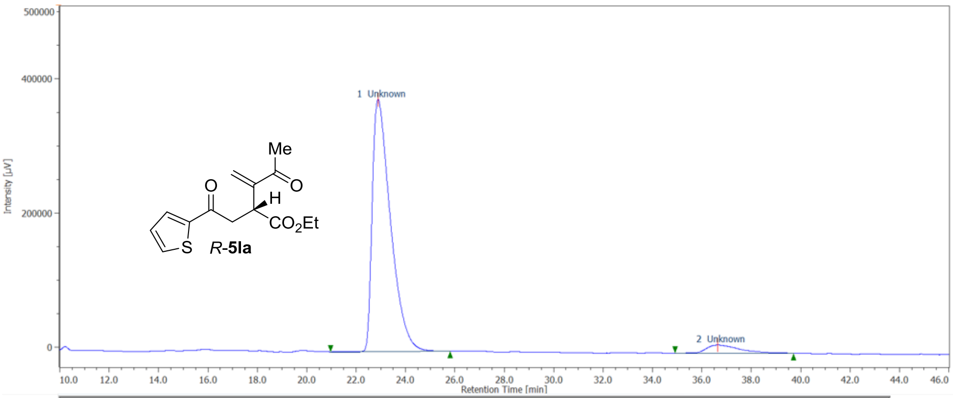

#	Peak Name	tR	Area	Height	Area%	Height%	Peak Start	Peak End	Base Start	Base End	Peak Mark
1	Unknown	22.883	19283043	375521	94.189	96.797	20.950	25.808	20.950	25.808	Manual
2	Unknown	36.625	1189685	12425	5.811	3.203	34.900	39.700	34.900	39.700	Manual

Supplementary Fig. 100. HPLC curve of *R*-**5la** catalyzed by poly($\mathbf{1s}_{50}$-*b*-$\mathbf{2}_{100}$) (Chiralpak OZ-H; *n*-hexane/*i*-PrOH = 85/15 (v/v); 1.00 mL/min; 220 nm; 25 °C).

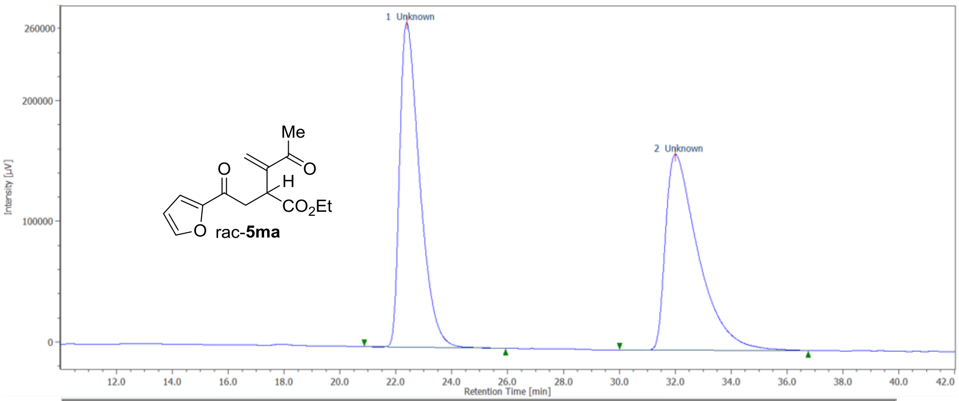

#	Peak Name	tR	Area	Height	Area%	Height%	Peak Start	Peak End	Base Start	Base End	Peak Mark
1	Unknown	22.383	13378669	268844	50.306	62.386	20.867	25.908	20.867	25.908	Manual
2	Unknown	31.992	13216039	162091	49.694	37.614	29.992	36.750	29.992	36.750	Manual

Supplementary Fig. 101. HPLC curve of *rac*-**5ma** catalyzed by poly($\mathbf{1s}_{50}$-*b*-$\mathbf{2}_{100}$) (Chiralpak OZ-H; *n*-hexane/*i*-PrOH = 80/20 (v/v); 1.00 mL/min; 220 nm; 25 °C).

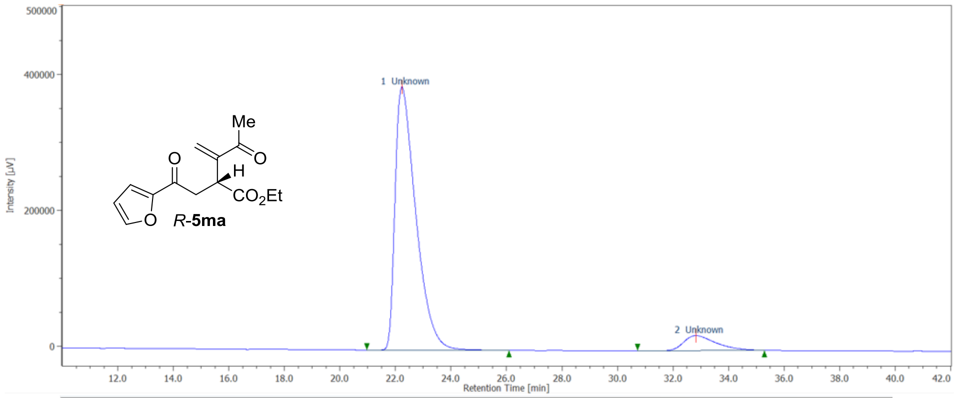

#	Peak Name	tR	Area	Height	Area%	Height%	Peak Start	Peak End	Base Start	Base End	Peak Mark
1	Unknown	22.225	20084064	386807	91.906	94.617	20.975	26.075	20.975	26.075	Manual
2	Unknown	32.800	1768781	22008	8.094	5.383	30.717	35.267	30.717	35.267	Manual

Supplementary Fig. 102. HPLC curve of *R*-**5ma** catalyzed by poly($\mathbf{1s}_{50}$-*b*-$\mathbf{2}_{100}$) (Chiralpak OZ-H; *n*-hexane/*i*-PrOH = 80/20 (v/v); 1.00 mL/min; 220 nm; 25 °C).

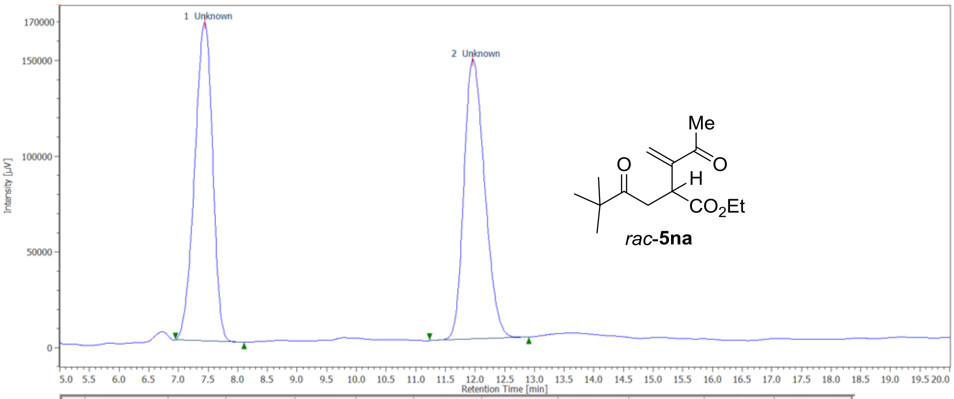

#	Peak Name	tR	Area	Height	Area%	Height%	Peak Start	Peak End	Base Start	Base End	Peak Mark
1	Unknown	7.442	3492174	166201	49.557	53.264	6.950	8.108	6.950	8.108	Manual
2	Unknown	11.958	3554588	145831	50.443	46.736	11.233	12.900	11.233	12.900	Manual

Supplementary Fig. 103. HPLC curve of *rac*-**5na** catalyzed by poly(**1s**$_{50}$-*b*-**2**$_{100}$) (Chiralpak OZ-H; *n*-hexane/*i*-PrOH = 85/15 (v/v); 1.00 mL/min; 220 nm; 25 °C).

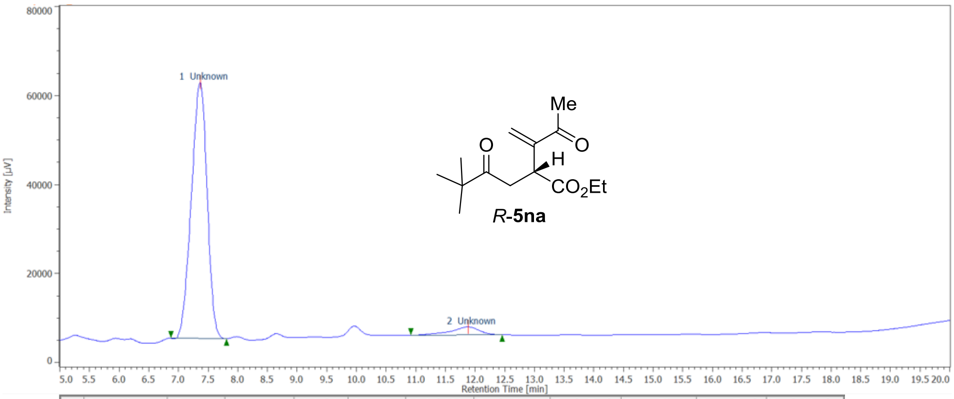

#	Peak Name	tR	Area	Height	Area%	Height%	Peak Start	Peak End	Base Start	Base End	Peak Mark
1	Unknown	7.367	1061507	57513	94.673	96.963	6.875	7.808	6.875	7.808	Manual
2	Unknown	11.875	59723	1802	5.327	3.037	10.917	12.450	10.917	12.450	Manual

Supplementary Fig. 104. HPLC curve of *rac*-**5na** catalyzed by poly(**1s**$_{50}$-*b*-**2**$_{100}$) (Chiralpak OZ-H; *n*-hexane/*i*-PrOH = 85/15 (v/v); 1.00 mL/min; 220 nm; 25 °C).

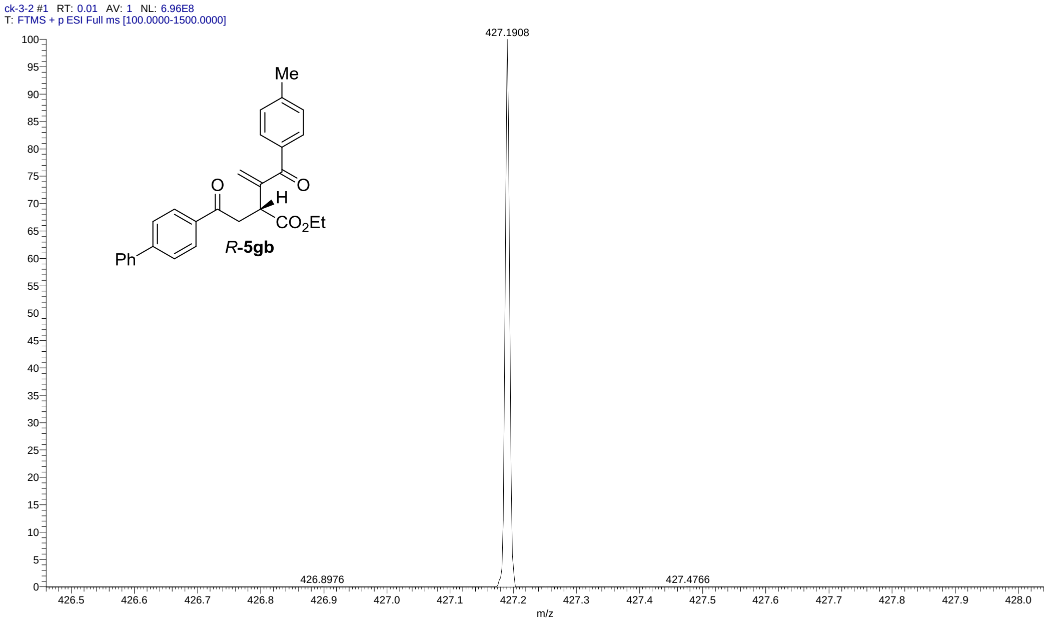

Supplementary Fig. 105. HRMS (ESI-FT) spectrum of the *R*-**5gb**.

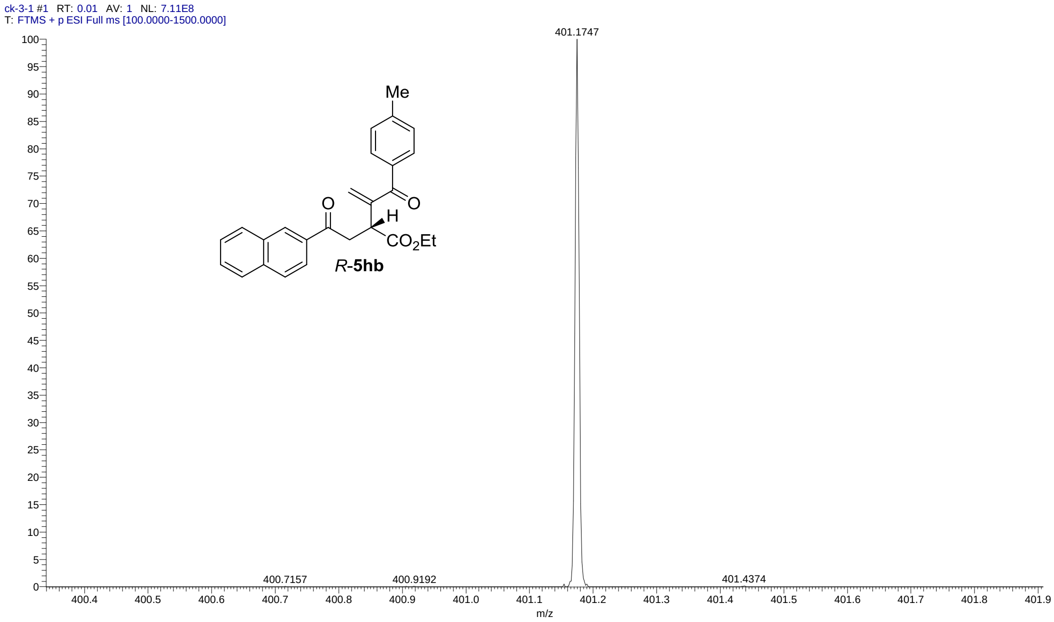

Supplementary Fig. 106. HRMS (ESI-FT) spectrum of the *R*-**5hb**.

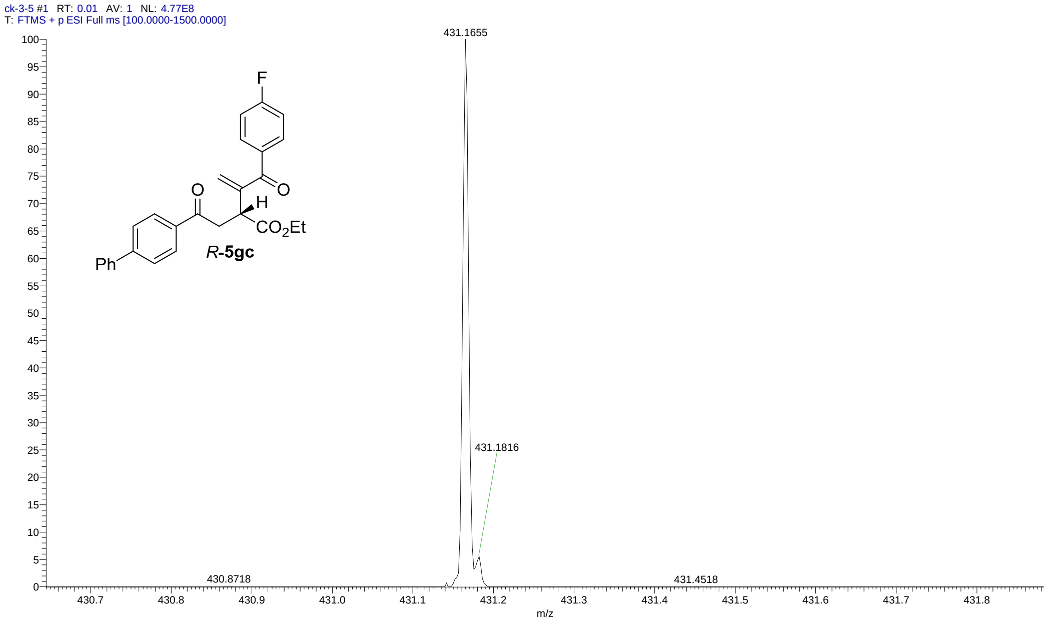

Supplementary Fig. 107. HRMS (ESI-FT) spectrum of the *R*-**5gc**.

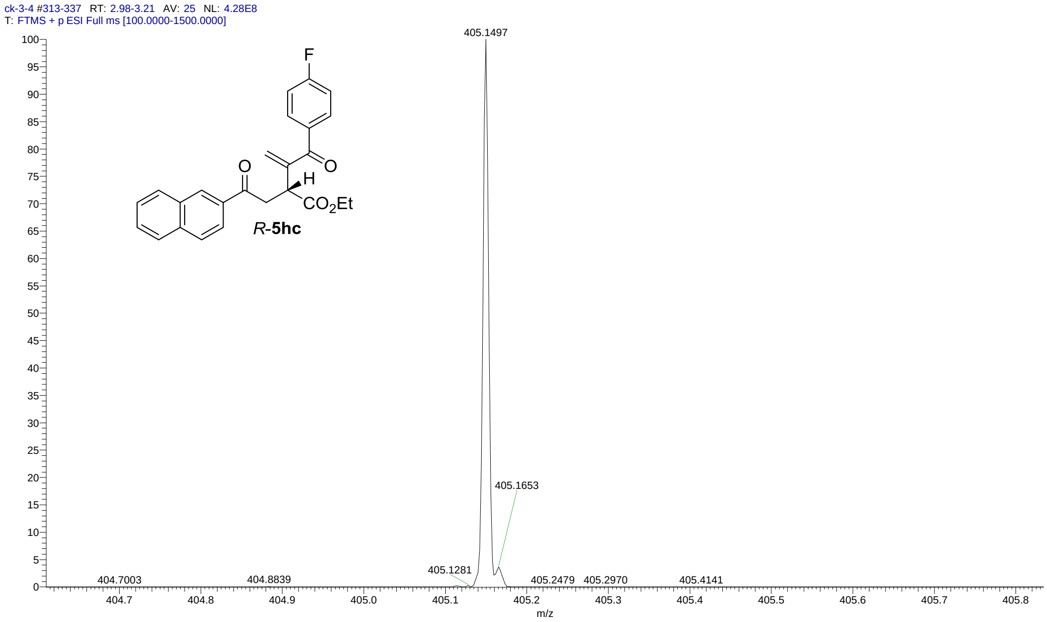

Supplementary Fig. 108. HRMS (ESI-FT) spectrum of the *R*-**5hc**.

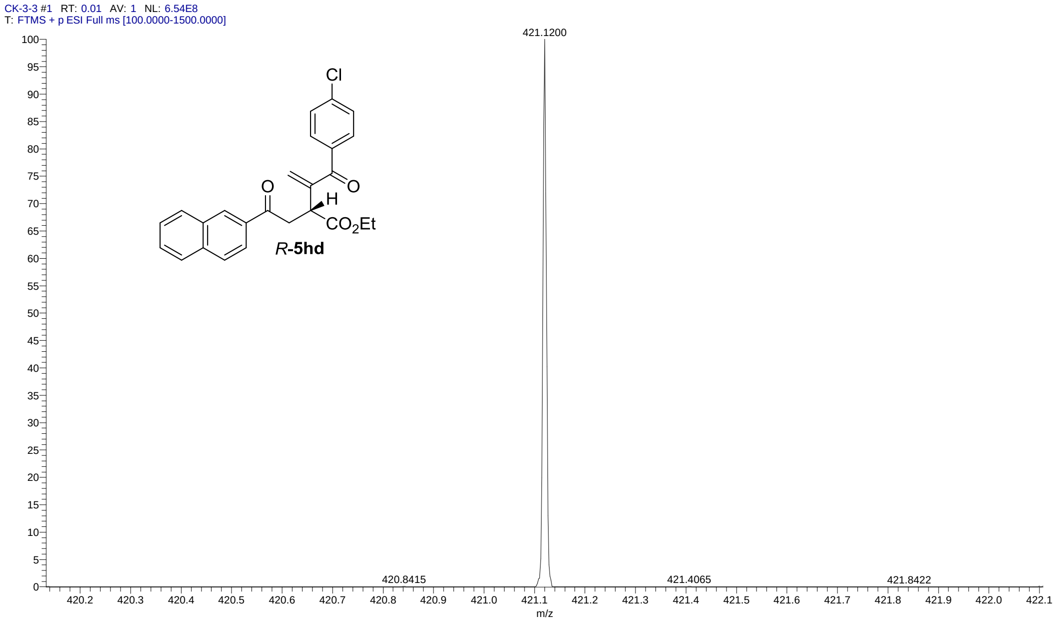

Supplementary Fig. 109. HRMS (ESI-FT) spectrum of the *R*-**5hd**.

References

1. Chen, J.-L.; Yang, L.; Wang, Q.; Jiang, Z.-Q.; Liu, N.; Yin, J.; Ding, Y.-S.; Wu, Z.-Q. Helix-sense-selective and enantiomer-selective living polymerization of phenyl isocyanide induced by reusable chiral lactide using achiral palladium initiator. *Macromolecules* **48**, 7737–7746 (2015).

2. Wang, Q.; Chu, B-F.; Chu, J.-H.; Liu, N.; Wu, Z.-Q. Facile synthesis of optically active and thermoresponsive star block copolymers carrying helical polyisocyanide arms and their thermo-triggered chiral resolution ability. A*CS Macro Lett*. **7**, 127−131 (2018).

3. Zhou, L.; Shen, L.; Huang, J.; Liu, N.; Zhu, Y.-Y.; Wu, Z.-Q. Optically active helical polyisocyanides bearing chiral phosphine pendants: facile synthesis and application in enantioselective rauhut-currier reaction. *Chinese J. Polym. Sci.* **36**, 163−170 (2018).

4. Lu, H.-H.; Wang, X.-F.; Yao, C.-J.; Zhang, J.-M.; Wu, H.; Xiao, W.-J. Highly enantioselective organocatalytic michael addition of nitroalkanes to 4-oxo-enoates. *Chem. Commun.* **28**, 4251−4253 (2009).

5. Zhou, W.; Su, X.; Tao, M.-N.; Zhu, C.-Z.; Zhao, Q.-J.; Zhang, J.-L. Chiral sulfinamide bisphosphine catalysts: design, synthesis, and application in highly enantioselective intermolecular cross-rauhut–currier reactions. *Angew. Chem. Int. Ed.* **54**, 14853−14857 (2015).

6. Creary, X. Reaction of Organometallic Reagents with Ethyl Trifluoroacetate and Diethyl Oxalate. Formation of Trifluoromethyl Ketones and α-Keto Esters vis Stable

Tetrahedral Adducts. *J. Org. Chem.* **52**, 5030−5032 (1987).
